# AlleleMiner: a long-read pipeline for gene-wise *de novo* allele phasing and variant detection in diploid citrus cultivars

**DOI:** 10.1093/dnares/dsag004

**Published:** 2026-03-03

**Authors:** Yukinari Kiryu, Yoshihiro Kawahara, Tomoko Endo, Tokumasa Horiike, Kenta Shirasawa, Sachiko Isobe, Takehiko Shimada, Hiroshi Fujii

**Affiliations:** Graduate School of Integrated Science and Technology, Shizuoka University, Shizuoka 422-8529, Japan; National Agriculture and Food Research Organization Advanced Analysis Center, Tsukuba 305-8604, Japan; National Agriculture and Food Research Organization Institute of Fruit and Tea Tree Science, Tsukuba 305-8505, Japan; Graduate School of Integrated Science and Technology, Shizuoka University, Shizuoka 422-8529, Japan; Kazusa DNA Research Institute, Kisarazu 292-0818, Japan; Kazusa DNA Research Institute, Kisarazu 292-0818, Japan; National Agriculture and Food Research Organization Institute of Fruit and Tea Tree Science, Tsukuba 305-8505, Japan; Faculty of Agriculture, Shizuoka University, Shizuoka 422-8529, Japan

**Keywords:** allele phasing, PacBio HiFi sequencing, *de novo* assembly, heterozygous crops, citrus genomics

## Abstract

Allelic variation is a critical determinant of agronomic traits in heterozygous crops. Most existing approaches define variation as reference-anchored differences, such as SNPs or structural variants, confining allelic diversity to variant feature coordinates. Here, we present AlleleMiner, a Python-based pipeline that phases diploid gene sequences directly from PacBio HiFi reads. Rather than relying on reference-based coordinate systems for allele representation, AlleleMiner uses the reference genome solely to identify target gene region sequences and performs *de novo* assembly of read sets at each locus, minimizing reference dependence and reconstructing phased allele sequences. Across 18 citrus cultivars, the pipeline achieved an average phasing output of 91.5% of 1,409 single-copy genes, with coverage achieving. Coverage analyses using both real and simulated datasets indicated that a ∼30× HiFi depth is preferable for the stable recovery of heterozygous alleles, reducing potential allele dropout. Validation using pedigree information showed allele transmission patterns with known relationships. Using simulated haplotype data and the *Citrus clementina* assembly v1.0, AlleleMiner achieved complete-match reconstruction for both alleles at approximately 70% of loci. By enabling reference-minimized gene-level allele discovery, AlleleMiner provides a scalable framework for constructing allele databases and advancing marker-assisted and genomic selection in complex crops.

## 1. Introduction

Long-read sequencing technologies have made it possible to generate phased genome assemblies that distinguish parental haplotypes, enabling comprehensive allele mining and systematic detection and comparison of allelic variants at specific gene loci. In heterozygous crops, such as citrus, identifying alleles linked to desirable traits remains a core challenge in advancing marker-assisted breeding. Previous studies on citrus carotenoids have demonstrated that metabolite accumulation depends on the coordinated expression of multiple biosynthetic and catabolic genes.^1,2^ For instance, in Satsuma mandarin (*Citrus unshiu*), β-cryptoxanthin content is determined not by a single gene but by the combined activity of *PSY*, *HYb*, *ZEP*, and *NCED*.^3,4^ Sugiyama et al.^5,6^ further reported allele-specific transcriptional variations in *PSY* and *ZEP*. Fujii et al.^7^ analysed these genes in 13 founders derived from Japanese breeding cultivars and identified distinct *PSY–ZEP* allele combinations that either enhanced or suppressed β-cryptoxanthin accumulation.

Beyond carotenoids, allelic variation underlies numerous agronomic traits in other fruit trees: Muscat aroma through *VvDXS* in grape^8,9^; powdery mildew resistance via *Run1*^10^ and *Ren1*^11^ ; fruit softening through *MdACO1* and *MdPG1* in apple^12^; and resistance to scab and fire blight through *Rvi6*^13^ and *FB_MR5*.^14^ Collectively, these findings highlight the broad influence of accurate allele identification on fruit quality and disease resistance.

However, most existing allele discovery approaches still rely on mapping reads to a single reference genome, where genetic variation is primarily represented as reference-anchored differences, such as single-nucleotide polymorphisms (SNPs), insertions and deletions (indels), or structural variants. Although this variant calling-based framework is effective for population-scale analyses, it constrains allelic diversity to collections of coordinate-dependent variant features and does not directly provide independent allele sequences that can be used as complete genetic units. This limitation is pronounced in highly heterozygous, clonally propagated crops,^15,16^ underscoring the need for gene-centred approaches that minimize reference dependence not only in detection but also in the representation of allelic variation.

Recent large-scale phasing studies in humans have demonstrated that algorithmic advances can now achieve near-perfect haplotype resolution across millions of samples, enabling parent-of-origin analyses.^17^ In parallel, new RNA-based long-read pipelines allow direct haplotype phasing and allele-specific expression analysis,^18^ and telomere-to-telomere reference maps are providing globally diverse haplotype panels for improved imputation and phasing accuracy.^19^ These developments highlight the broader momentum towards reference-independent high-fidelity phasing frameworks across species.

Despite these advances, most existing approaches focus on population-scale or genome-wide haplotyping, whereas comparatively few tools address the gene-wise reconstruction of phased allele sequences in plant genomes. Although long-read sequencing data are increasingly available, automated pipelines that perform locus-specific phasing and allele reconstruction in a scalable and reference-minimized manner remain scarce. Here, we present AlleleMiner, a Python-based pipeline designed for the gene-level phasing of diploid genomes. AlleleMiner uses the reference genome only to define target gene region sequences (TGRS), extracts long reads spanning each locus, and reconstructs alleles through *de novo* assembly, reducing reference bias while retaining locus specificity. By reconstructing alleles, AlleleMiner enables the identification and tracking of functional alleles within founder-derived gene pools, supporting marker-assisted selection of optimal allele combinations in perennial fruit tree breeding programmes.

We applied AlleleMiner to 18 citrus cultivars comprising 1,409 target genes, evaluated its performance across a range of sequencing coverages, and assessed allele reconstruction accuracy using simulated haplotype data and homologous loci retrieved from the clementine genome v1.0^20^ as a surrogate parental reference. In addition, we examined allele transmission consistency using pedigree information, demonstrated the detection of large heterozygous structural insertions, and tested the robustness of the pipeline on a duplicated gene family. Together, these analyses establish AlleleMiner as a practical framework for reference-minimized, gene-centred allele discovery in heterozygous crops.

## 2. Materials and methods

### 2.1. Software implementation

AlleleMiner was developed in Python 3 for Linux/UNIX environments. The pipeline integrates multiple third-party tools: SeqKit (ver. 2.2.0)^21^ for FASTA/FASTQ manipulation; GFF3toolkit (ver. 2.1.0)^22^ for extracting gene sequences corresponding to annotated regions from the GFF3 annotation and the reference genome FASTA files (ref-GFF3 and ref-genome); Minimap2 (ver. 2.29-r1283)^23^ for long-read mapping; and two *de novo* assemblers, Hifiasm (ver. 0.25.0-r726)^24^ and Flye (ver. 2.9.5-b1801),^25^ for PacBio HiFi assemblies.

**Fig. 1.**
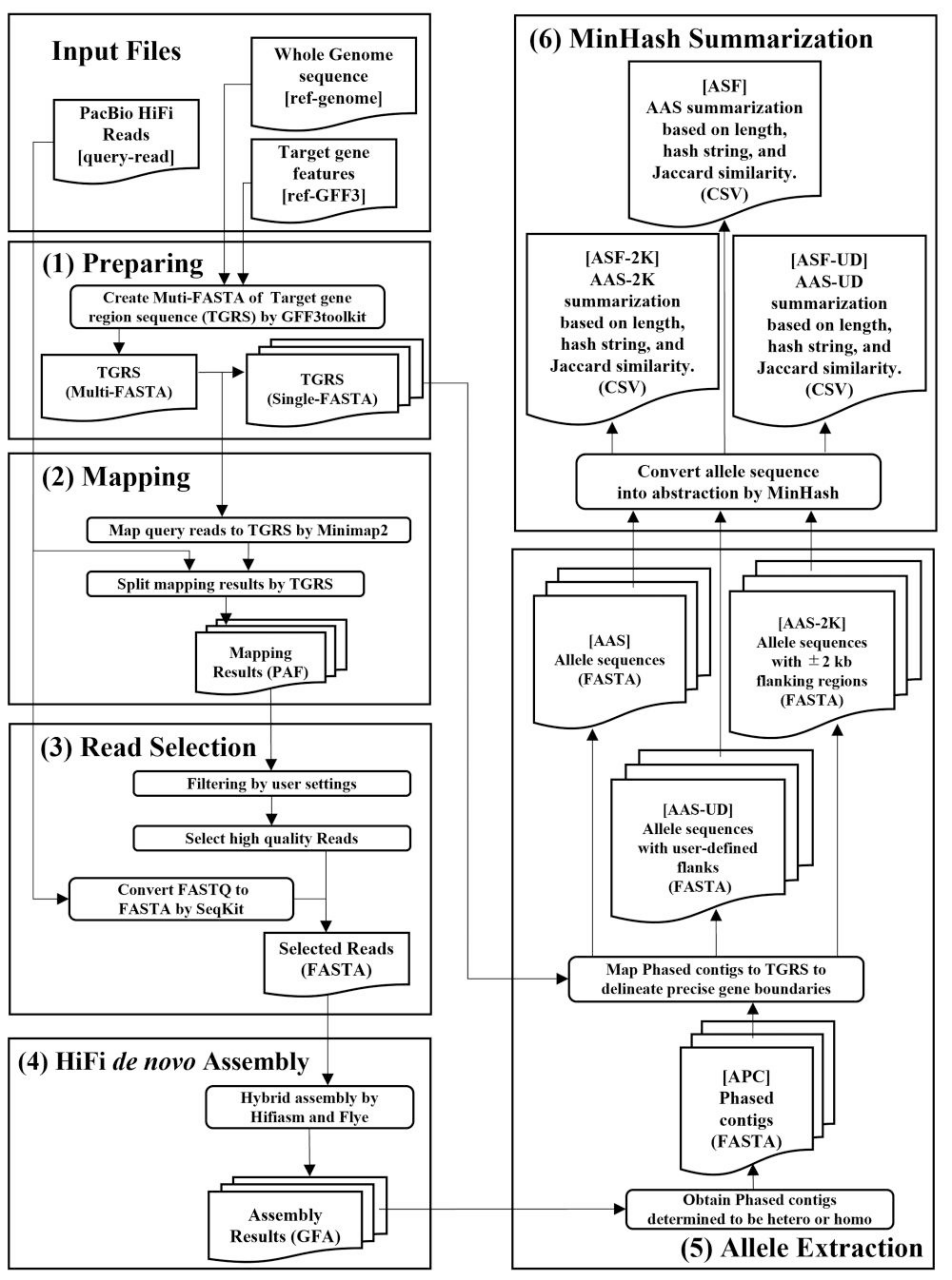
Workflow of the AlleleMiner algorithm. Abbreviations: APC, AlleleMiner-Phased Contigs; AAS, AlleleMiner-Allele Sequences; AAS-2K, AAS with ±2 kbp flanking regions; AAS-UD, AAS with User-Defined flanking regions; ASF, AlleleMiner-Summary Files; ASF-2K, ASF with ±2 kbp flanking regions; ASF-UD, ASF with User-Defined flanking regions.

In addition, Python libraries such as Biopython (ver. 1.85)^26^ and Datasketch (ver. 1.6.5; https://github.com/ekzhu/datasketch) were used for sequence manipulation and MinHash-based similarity estimation, respectively. AlleleMiner requires three input files: (i) PacBio HiFi reads (query-reads), (ii) the reference whole-genome sequence (ref-genome), and (iii) a gene annotation file in GFF3 format specifying target genes (ref-GFF3) (Fig. 1). A Python wrapper controls the entire workflow and outputs both phased contigs and allele-specific sequences.

### 2.2. Workflow

**Fig. 2.**
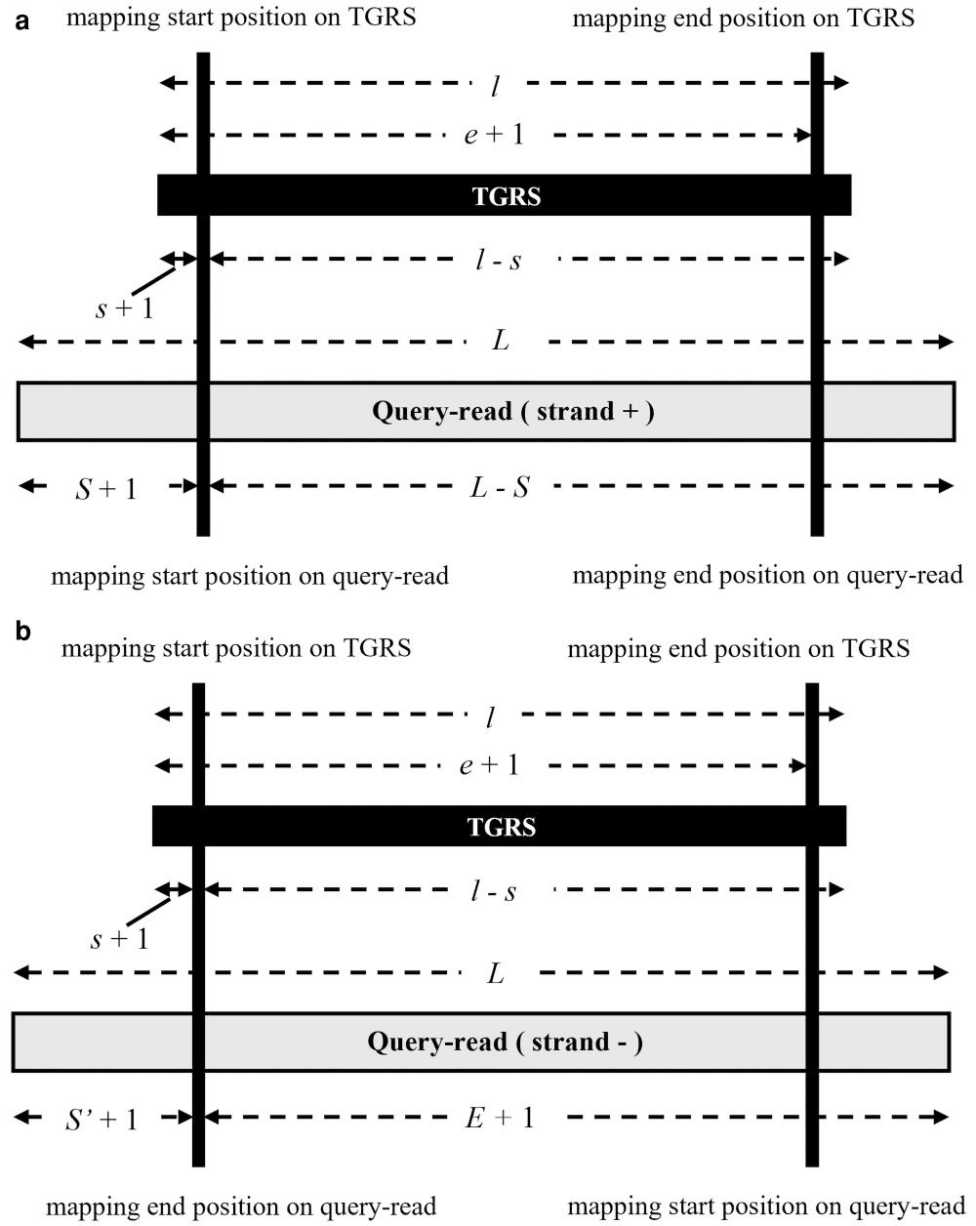
Selection of reads spanning the entire TGRS region. “Spanning” is defined as reads whose 5′ end lies upstream of the TGRS start and whose 3′ end lies downstream of the TGRS end. Shown are: A: For query reads mapped to the TGRS on the + strand, let *L* be the query read length, *l* the TGRS length, *S* the mapping start position on the query read, *s* the mapping start position on the TGRS, and *e* the mapping end position on the TGRS. Query reads satisfying [(*e* + 1)−(*s* + 1) > *l* × 0.95] ∧ [*L* > *l*] ∧ [*S* + 1 > *s* + 1] ∧ [*L* − *S* > *l* − *s*] were selected. B: For query reads mapped on the – strand, let *E* be the mapping end position on the query read and *S*′ the inverted mapping start position (*S*′ = *L* − *E*−1). Query reads satisfying [(*e* + 1)−(*s* + 1) > *l* × 0.95] ∧ [*L* > *l*] ∧ [*S′* + 1 > *s* + 1] ∧ [*E* + 1 > *l* − *s*] were selected. For both A and B, only reads with a Minimap2 mapping score of ≥60 were considered. The addition of “+1” to each coordinate accounts for the 0-based coordinate system used in Minimap2 output. TGRS, target gene region sequence.

AlleleMiner operates through six sequential steps (Fig. 1). First, target gene region sequences (TGRS) are extracted from the reference genome based on positions defined in the GFF3 annotation file, and a separate FASTA file is generated for each gene. Second, query reads are mapped to each TGRS using Minimap2. Third, mapped reads are filtered by mapping quality (≥60) and coverage, and only those spanning at least 95% of the TGRS are retained. Strand-specific criteria are applied to ensure that each selected read fully covers the gene region (Fig. 2). Fourth, selected reads are assembled using Hifiasm; if no contigs are produced, Flye is used as a fallback assembler. Two contigs indicate a heterozygous locus, while a single contig denotes homozygosity. Fifth, we aligned as AlleleMiner-Phased Cotigs (APC) back to the TGRS to delineate precise gene boundaries. Then pipeline outputs sequence as AlleleMiner-Allele Sequences (AAS), AAS with ±2 kbp flanking regions (AAS-2K), and AAS with User-Defined flanking regions (AAS-UD). Finally, for each allele, zygosity, sequence length, Jaccard similarity, and MinHash values are computed and stored in comma-separated summary files: AlleleMiner-Summary Files (ASF), ASF with ±2 kbp flanking regions (ASF-2K) and ASF with User-Defined flanking regions (ASF-UD).

### 2.3. Datasets

#### 2.3.1. PacBio HiFi reads

High-fidelity (HiFi) sequencing datasets were collected for 18 citrus cultivars: Satsuma mandarin (*C. unshiu*) (STS), Kishu mandarin (*C. kinokuni*) (KSH), Kunenbo mandarin (*C. nobilis* Lour. var. *knep* Tanaka) (KNN), pummelo (*C. maxima*) (PMM), Hassaku (*C. hassaku*) (HSS), grapefruit (*C. paradisi*) (GRP), sweet orange (*C. sinensis*) (SWT), Hyuganatsu (*C. tamurana* hort. ex. Tanaka) (HYG), Iyokan (*C. iyo* hort. ex. Tanaka) (IYK), Ponkan (*C. reticulata*) (PNK), Dancy tangerin (*C. tangerina* hort. ex. Tanaka) (DNC), King mandarin (*C. nobilis*) (KNG), Murcott tangor (MRC), Mediterranean mandarin (*C. deliciosa*) (MDT), Mukaku Kishu (a seedless mutant of KSH) (MKK), lemon (*C. limon*) (LMN), citron (*C. medica*) (CTR), and oval kumquat (*Fortunella margarita*) (OVL). In this study, we use the term “cultivar” for the analysed accessions. Species names are reported according to the Tanaka^27^ classification, which is still widely used in the citrus community and in public repositories such as NCBI and DDBJ, although many cultivars are now recognized as interspecific hybrids based on genome analyses.

Of these, 14 cultivars were downloaded from BioProject PRJDB15866,^28^ whereas the remaining four (ie, MKK, LMN, CTR, and OVL) were sequenced separately following the protocol of Isobe et al. (2023). The HiFi reads exhibited mean lengths of roughly 10 to 25 kbp and average quality scores exceeding Q20, generating 4.1 to 15.4 Gbp of data per sample (see Watanabe et al. 2025^29^ and Table S1). All datasets are publicly accessible under BioProject PRJDB15974.

#### 2.3.2. Satsuma mandarin genome as reference

The Satsuma mandarin genome (CUNunp r1.0)^28^ was retrieved from MiGD (https://mikan.dna.affrc.go.jp) and used as the reference genome (ref-genome_STS). A corresponding GFF3 annotation file containing 1,409 single-copy gene positions was created using the BUSCO embryophyta_odb10 database (https://busco-data.ezlab.org/v5/data/lineages/) by identifying orthologous genes shared between the reference assembly and the BUSCO lineage dataset (ref-GFF3_STS_BUSCO).

#### 2.3.3. *Citrus clementina*, assembly v1.0 as reference

We used the *Citrus clementina* (CLM) genome assembly v1.0^20^ (hereafter Ccl v1.0), obtained from Phytozome, as the standard citrus reference genome (ref-genome_CLM). By identifying orthologous genes shared between Ccl v1.0 and the BUSCO Embryophyta lineage dataset (embryophyta_odb10), we generated a GFF3 annotation file (ref-GFF3_CLM_BUSCO) containing 1,159 single-copy gene positions and the corresponding FASTA-format gene sequence set (CLM_BUSCO_GENES).

### 2.4. Generation of simulated data for simulation

#### 2.4.1. Generation of simulated haplotype sequences

To evaluate the reliability of AlleleMiner using simulated sequences, we generated simulated sequences, HAP_AL and HAP_AH. We created two haplotype sequence sets: HAP_BL, which incorporates low-frequency variants into HAP_AL, and HAP_BH, which incorporates high-frequency variants into HAP_AH. GFF3 files (GFF3_AL, GFF3_AH, GFF3_BL, and GFF3_BH) were created to contain 1,000 genes each, with TGRS specified. The gene sequence length was randomly set between 1,000 and 5,000 bp. TGRS sequence sets (ALLELE_AL, ALLELE_AH, ALLELE_BL, and ALLELE_BH) were created. Identical gene IDs were assigned to the same locus to indicate the relationship between alleles. A custom Python program was used to generate these data.

The low- and high-frequency variant models refer to simulated haplotype models in which variants are introduced at low and high frequencies, respectively, into each haplotype sequence. For the low-frequency variant model, we referenced the literature on rice, and for the high-frequency variant model, we referenced the literature on citrus. However, the purpose of this analysis was not to accurately simulate the variability of rice and citrus but to evaluate the allele reproducibility of AlleleMiner in low-frequency variants and high-frequency variant models using simulated genomes. Furthermore, the rationale for the parameters required to generate realistic models has not been detailed in the literature. Therefore, some arbitrariness sets the parameters to generate models while maintaining biological plausibility.

To generate the low-frequency variants model, we referenced the variant rate of Japonica rice, known for its low genomic diversity.^30–32^ The SNP variant rate for HAP_BL was set to 1.5 SNPs/kbp and the structural variation (SV) rate was set to 0.1 SV/kbp. Furthermore, the SV occurrence rate was set at 80% for SVs of 1 to 10 bp, 15% for 11 to 50 bp, 3% for 51 to 100 bp, 1% for 101 to 200 bp, 0.5% for 201 to 500 bp, 0.3% for 501 to 1,000 bp, 0.15% for 1,001 to 5,000 bp, 0.04% for 5,001 to 10,000 bp, and 0.01% for 10,001 to 50,000 bp.

To generate a high-frequency variant model, we referenced the variant rates in citrus, which showed strong heterozygosity.^33,34^ In HAP_BH, the SNP frequency was set to 17 SNPs/kbp, and the SV rate was set to 1.8 SV/kbp For the SV size distribution, we applied 69% of the 1.8 SV/kbp rate for SVs of 1 to 10 bp, 10% for 11 to 50 bp, 6% for 51 to 100 bp, 2.75% for 101 to 200 bp, 3.6% for 201 to 500 bp, 3% for 501 to 1,000 bp, 3.65% for 1,001 to 5,000 bp, 1% for 5,001 to 10,000 bp, and 0.01% for 10,001 to 50,000 bp.

#### 2.4.2. Generation of simulated reads

Using PBSIM,^35^ simulated HiFi reads named READ_L and READ_H were generated for the HAP_AL and HAP_BL sets, and for the HAP_AH and HAP_BH sets, respectively. Six coverage conditions were set: 10×, 20×, 30×, 42×, 50×, and 60×. The 42× setting was based on the coverage of the actual STS data. The parameters for the read length distribution were set based on actual long read data from STS, with length-mean set to 21,000 bp, length-min to 10,000 bp, length-max to 30,000 bp, and length-sd to 2,000 bp.

### 2.5. AlleleMiner analysis of 18 cultivars

AlleleMiner analysis was performed on the 18 cultivars described in Section 2.3.1. PacBio HiFi reads from each cultivar were used as query-reads. The reference genome was ref-genome_STS, and the reference GFF3 was ref-GFF3_STS_BUSCO. In this analysis, an AlleleMiner run was considered successful when it recovered one (homozygous) or two (heterozygous) allele sequences from a diploid locus.

### 2.6. Examination of coverage

Using real and simulated data, we evaluated the changes in AlleleMiner output across different coverage levels. In this evaluation, an AlleleMiner run was considered successful when it recovered one (homozygous) or two (heterozygous) allele sequences for diploid loci.

#### 2.6.1. Examination of coverage using real data

PacBio HiFi reads (42.9×) obtained from STS were downsampled using Rasusa^36^ to generate datasets with 30×, 20×, and 10× coverage. These were used as query reads, with ref-genome_STS as the ref-genome and GFF3_STS_BUSCO as the ref-GFF3. AlleleMiner analysis was performed, and the number of outputs was calculated.

#### 2.6.2. Examination of coverage using simulated data

The six coverage conditions for both low- and high-frequency variant models are described in Section 2.4.2. were used as query reads, with HAP_AH and HAP_AL as the ref-genome and GFF3_AH and GFF3_AL as the ref-GFF3. AlleleMiner analysis was performed, and the number of outputs was calculated.

### 2.7. Verification using simulated data

To verify AlleleMiner output using simulated reads, we executed AlleleMiner with HAP_AH and HAP_AL as the ref-genome, GFF3_AH and GFF3_AL as the ref-GFF3 and READ_H30 and READ_L30 (created under 30× coverage conditions) as query reads. We verified whether the allele sequences obtained from AlleleMiner output (am_hap_1, am_hap_2, am_homo) perfectly matched the ALLELE_AH and ALLELE_AL sequence used as the TGRS and its corresponding ALLELE_BH or ALLELE_BL sequence.

### 2.8. Verification using the clementine genome

As shown in Section 2.3.3, CLM_BUSCO_GENES were used as the TGRS. The validity of this method was evaluated by comparing whether the allele sequences reconstructed by AlleleMiner using PacBio HiFi from SWT and MDT, the parent strains of CLM perfectly matched the TGRS (CLM_BUSCO_GENES). Specifically, ref-genome_CLM was used as the ref-genome, ref-GFF3_CLM_BUSCO was used as the ref-GFF, and PacBio HiFi reads of MDT and SWT were analysed as query reads.

### 2.9. Verification of AlleleMiner output based on allele transmission in pedigree

We validated the full genomic sequences of alleles, including introns, untranslated regions (UTRs), and 2,000 bp upstream regulatory regions, for three single-copy genes of CUNunp r1.0: *CUN7G148100.t1* (encoding the receptor-like protein TOO MANY MOUTHS, TMM),^37^  *CUN2G259500.t1* (encoding Y-family DNA polymerase η),^38^ and *CUN1G201600.t1* (encoding the RING-type E3 ligase ABI3 INTERACTING PROTEIN 2, AIP2).^39^ Gene functions were verified by sequence similarity searches against the TAIR database (https://www.arabidopsis.org/).

These three genes were chosen because TMM mutations disrupt stomatal patterning, polymerase η catalyses translesion synthesis of UV-damaged DNA, and AIP2 mediates degradation of the transcription factor FUSCA3 during embryogenesis. Using ref_genome_STS as the reference genome, the TGRS of three genes as the reference GFF3, and PacBio HiFi reads from 18 cultivars as query reads, AlleleMiner analysis was performed.

Next, allele sequences were aligned using MUSCLE (ver. 5.1),^40^ and maximum-likelihood phylogenetic trees were generated with RAxML (ver. 8.2.11)^41^ under the GTR model with 1,000 bootstrap replicates, as implemented in Geneious Prime Build 2025-08-20 12:11 (Dotmatics, Boston, USA).

Known pedigrees, (1) STS as the offspring of KSH × KNN^42^; (2) MKK as a mutant of KSH^27^; (3) KNN as a progeny of KSH^43^; and (4) CLM as a hybrid of SWT × MDT,^44^ were used to assess allelic transmission. The corresponding genes in CLM were *Ciclev10014398m*, *Ciclev10018447m*, and *Ciclev10025464m*, retrieved from the Ccl v1.0.

A mismatch tolerance of one error per kbp was applied, consistent with the reported HiFi read accuracy of 99.5%–99.9%.^45,46^

### 2.10. Validation of AlleleMiner output by *CCD4* gene family

To evaluate the capacity of AlleleMiner to phase duplicated genes, we examined the *CCD4* subfamily (*CCD4a*, *CCD4b1*, *CCD4b2*, *CCD4c*, and *CCD4d*).^47–50^ Because citrus is diploid, these *CCD4* members were treated as paralogous loci (not polyploid-derived homologs), and Allele Miner was applied to each locus independently. Alleles were phased from STS, KSH, and KNN using the Ccl v1.0 as the reference. Genomic sequences, including introns and UTRs, for *Ciclev10031003m*, *Ciclev10028113m*, *Ciclev10030384m*, *Ciclev10011335m*, and *Ciclev10013726m* were obtained from Phytozome for comparative analysis.

## 3. Results

### 3.1. Performance of AlleleMiner

Across 18 cultivars and 1,409 target genes, AlleleMiner successfully phased an average of 1,228.7 genes per cultivar, corresponding to a mean success rate of 91.5% (Table 1). Heterozygosity varied widely among cultivars; KSH showed the highest heterozygosity (number of heterozygous sites/total sites = 0.77), whereas CTR was nearly homozygous (0.01). Success rates were positively correlated with sequencing depth. Success rates were positively correlated with sequencing depth (Pearson’s correlation coefficient *r* = 0.795) (Figure S1).

**Table 1.** AlleleMiner output counts per cultivar (18 citrus cultivars) and their range, mean, and standard deviation across loci.

Cultivar (abbreviation)	No. of AlleleMiner output	Ratio of AlleleMiner output (%)	No. of heterozygous	No. of homozygous	Heterozygosity	Coverage (total bases/360MB)
**STS**	1,388	98.5	799	589	0.58	42.9
**KSH**	1,372	97.4	1,062	310	0.77	30.9
**KNN**	1,366	96.9	988	378	0.72	26.3
**PNK**	1,348	95.6	998	350	0.74	21.5
**MDT**	1,342	95.2	865	477	0.64	23.0
**MKK**	1,331	94.5	895	436	0.67	18.9
**IYK**	1,318	93.5	770	548	0.58	19.2
**KNG**	1,308	92.8	810	498	0.62	17.9
**LMN**	1,306	92.7	990	316	0.76	21.7
**CTR**	1,303	92.5	9	1,294	0.01	23.8
**HYG**	1,287	91.3	882	405	0.69	18.2
**SWT**	1,278	90.7	783	495	0.61	16.0
**OVL**	1,268	90.0	848	420	0.67	19.8
**HSS**	1,257	89.2	784	473	0.62	16.7
**MRC**	1,238	87.9	484	754	0.39	13.3
**PMM**	1,185	84.1	531	654	0.45	13.2
**DNC**	1,156	82.0	352	804	0.30	11.3
**GRP**	1,145	81.3	496	649	0.43	13.5
**Range**	1,388 to 1,145	98.5 to 81.3	1,062 to 9	1,294 to 310	0.77 to 0.01	42.9 to 11.3
**Average**	1,228.7	91.5	741.4	547.2	0.58	20.5
**Standard deviation (SD)**	69.0	…	269.2	234.1	…	…

Abbreviations: STS, Satsuma mandarin; KSH, Kishu mandarin; KNN, Kunenbo mandarin; PNK, ponkan madnarin; MDT, Mediterranean mandarin; MKK, Mukaku Kishu; IYK, Iyokan; KNG, King mandarin; LMN, lemon; CTR, citron; HYG, Hyuganatsu; SWT, sweet orange; OVL, oval kumquat; HSS, hassaku; MRC, Murcott tangor; PMM, pummelo; DNC, Dancy tangerine; GRP, grapefruit.

Among cultivars with coverage exceeding 20× (STS, 42.9×; KSH, 30.9×; KNN, 26.3×; PNK, 21.5×; MDT, 23.0×; LMN, 21.7×; CTR, 23.8×), five achieved success rates above 95%, while two ranged between 90% and 95%. Conversely, among cultivars with coverage below 20× (MKK, 18.9×; IYK, 19.2×; KNG, 17.9×; HYG, 18.2×; SWT, 16.0×; OVL, 19.8×; HSS, 16.7×; MRC, 13.3×; PMM, 13.2×; DNC, 11.3×; GRP, 13.5×), none reached 95%. Six cultivars achieved success rates between 90% and 95%, whereas another five cultivars fell below 90%. The average percent of failures per cultivar at each step of AlleleMiner workflow in Fig. 1 was 0.6% at the mapping step, 12.3% at the read selection stage, 78.8% at the HiFi *de novo* assembly step, and 8.2% at the allele extraction stage (Table S2).

To illustrate the computational performance, AlleleMiner analysis of the cultivar with the largest read file and highest coverage, STS (42.9×), required approximately [Time; 2.83 h, Max memory 5.9 GB], while that of the lowest-coverage cultivar, DNC (11.3×), completed in [Time; 1.56 h, Max memory; 2.2 GB]. Both analyses were performed on a workstation (Intel Core i9-14900 K, 24-core CPU, 94 GB RAM, Ubuntu 24.04.2 LTS).

Table S3 presents zygosity and allele sequence lengths for one cultivar (MDT), along with MinHash values and Jaccard coefficients between alleles.

### 3.2. Coverage-dependent behaviour of AlleleMiner output

#### 3.2.1. Coverage-dependent behaviour of AlleleMiner using real HiFi data

**Fig. 3.**
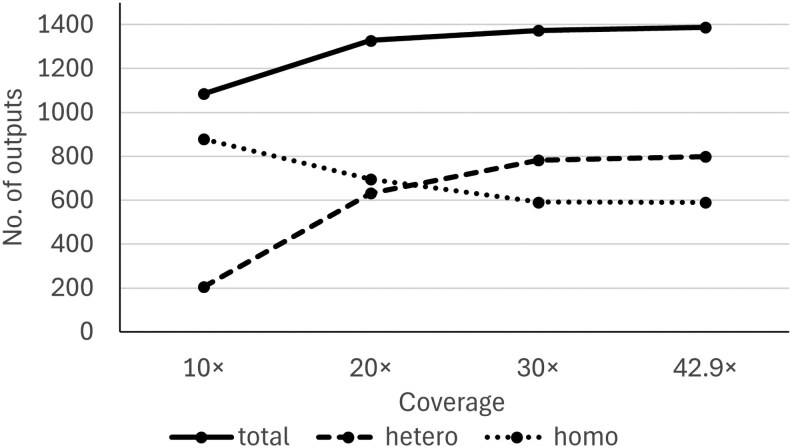
Variation in AlleleMiner output counts due to coverage differences in real data. PacBio HiFi reads (42.9×) derived from STS were down sampled using Rasusa (Hall 2021) to create datasets with 10×, 20×, 30×, and 42× coverage. AlleleMiner analysis was performed using ref-genome_STS and GFF3_STS as reference. The number of AlleleMiner outputs increased with coverage. Abbreviations: STS, Satsuma mandarin.

Using the STS real data shown in Section 2.6.1, we evaluated the relationship between coverage and the number of AlleleMiner outputs. The results showed that the number of outputs increased with increasing coverage (Fig. 3), peaking at 1,388 outputs (799 heterozygotes and 589 homozygotes) at 42.9× coverage. At 30× coverage, 1,373 outputs (782 heterozygous and 591 homozygous) were obtained, corresponding to 98.9% of the output count at 42.9× (97.9% for heterozygous and 100% for homozygous).

#### 3.2.2. Coverage-dependent behaviour of AlleleMiner using simulated data

**Fig. 4.**
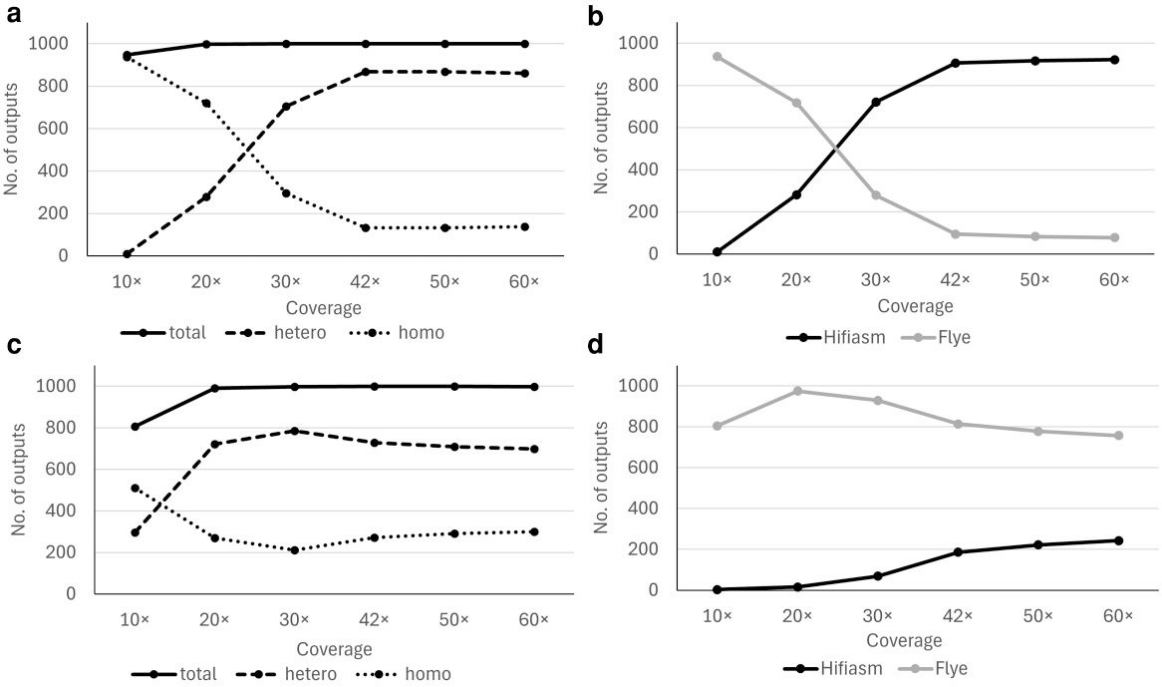
Relationship between coverage and AlleleMiner output counts in simulated reads and source assemblers. The horizontal axis shows the coverage, and the vertical axis shows the number of AlleleMiner outputs. A and B show the results for simulated data based on low-frequency variant models, whereas C and D show the results based on high-frequency variant models. Panels A and C display the number of AlleleMiner outputs obtained under different coverage conditions, whereas panels B and D show outputs by the assembler that generated them.

Using simulated reads, we verified the relationship between coverage and AlleleMiner output counts. As coverage increased, the output counts showed an overall increasing trend (Fig. 4a, c). In the low-frequency variant model, total output counts plateaued at 20×, with heterozygous outputs peaking at 42× and 50×. The high-frequency variant model also showed total output plateauing at 20×, with heterozygous outputs peaking at 30×. Comparing assemblers generating outputs, Flye tended to produce more outputs under low-coverage conditions in both models, whereas Hifiasm tended to produce more outputs under high-coverage conditions (Fig. 4b, d). This trend was more pronounced in the low-frequency variant model. Furthermore, Flye generated more outputs in the high-frequency variant model, whereas Hifiasm generated more outputs in the low-frequency variant model.

### 3.3. Accuracy of allele reconstruction using simulated haplotype data

#### 3.3.1. Allele reconstruction accuracy under a low-frequency variant model

Among the am_hap_1, am_hap_2, and am_homo sequences output by AlleleMiner, 817 sequences (81.7%) perfectly matched ALLELE_AL, 806 sequences (80.6%) perfectly matched ALLELE_BL, and 654 sequences (65.4%) were heterozygous, perfectly matching both ALLELE_AL and ALLELE_BL. Furthermore, in the low-frequency variant model, there were 32 loci where ALLELE_AL and ALLELE_BL were homozygous, and 31 sequences perfectly matched am_homo. Therefore, the number of loci at which AlleleMiner correctly output the allele was 685. There were 61 loci where the AlleleMiner output matched neither ALLELE_AL or ALLELE_BL, and one locus where no output was obtained.

#### 3.3.2. Allele reconstruction accuracy under a high-frequency variant model

Among the am_hap_1, am_hap_2, and am_homo outputs from AlleleMiner, 980 sequences (98.0%) perfectly matched ALLELE_AH, 680 sequences (68.0%) perfectly matched ALLELE_BH, and 670 sequences (67.0%) perfectly matched both ALLELE_AH and ALLELE_BH in heterozygous states. Because no loci showed homozygous ALLELE_AH and ALLELE_BH in the high-frequency variant model, AlleleMiner correctly output allele sequences for 670 loci. There were seven loci where AlleleMiner output matched neither ALLELE_AH or ALLELE_BH, and three loci where no output was obtained.

### 3.4. Validation of allele reconstruction using the clementine genes

**Fig. 5.**
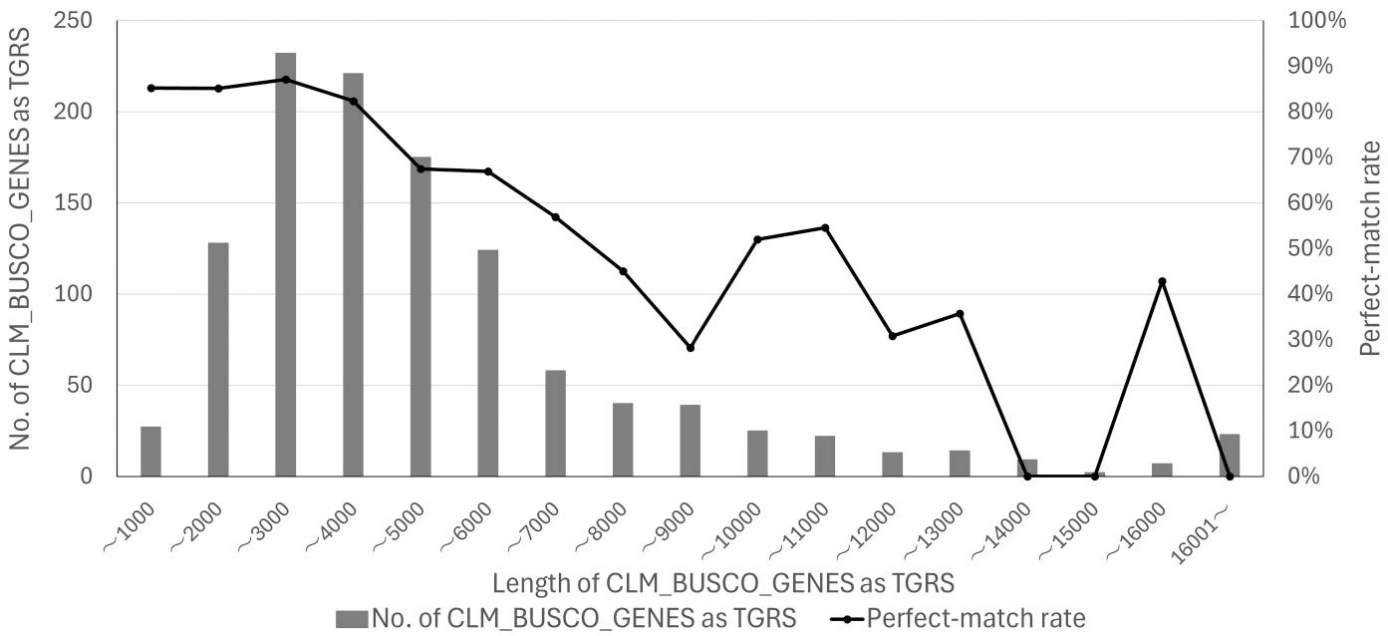
Perfect match rate of AlleleMiner-reconstructed alleles to the CLM_BUSCO_GENES with the length and locus counts per length bin. Across 1,159 CLM_BUSCO_GENES defined by ref-genome_CLM_BUSCO and ref-GFF3_CLM_BUSCO as TGRS, represents the fraction of loci for which at least one of the allele sequences reconstructed by AlleleMiner from the two parental cultivars (MDT and SWT) perfectly matched the corresponding CLM_BUSCO_GENES at the loci. The bars show the number of CLM_BUSCO_GENES loci in each-length bin. CLM is a progeny of MDT × SWT. Abbreviations: CLM, clementine; MDT, Mediterranean mandarin; SWT, sweet orange; TGRS, target gene region sequence. CLM_BUSCO_GENES refer to Section 2.3.3.

At 1,159 loci, the MDT- or SWT-derived allele sequences output by AlleleMiner perfectly matched the CLM_BUSCO_GENES in 816 cases (70.4%). Because the CLM_BUSCO_GENES defined in Section 2.3.3 serve as a proxy reference for parental alleles, this perfect match reflects the accuracy of AlleleMiner's parental allele reconstruction in this analysis. The percentage of perfect matches tended to decrease with increasing gene sequence lengths (Fig. 5). However, for genes under 6,000 bp, which comprised 78% of all loci, matches were confirmed in 79% of cases. The longest gene for which a match was confirmed was 15,605 bp. These results indicate that AlleleMiner possesses high reconstruction accuracy at practical gene-length scales.

### 3.5. Verification of AlleleMiner output based on allele transmission in pedigree

#### 3.5.1. Pedigree-consistent allele transmission at CUN7G148100.t1

**Fig. 6.**
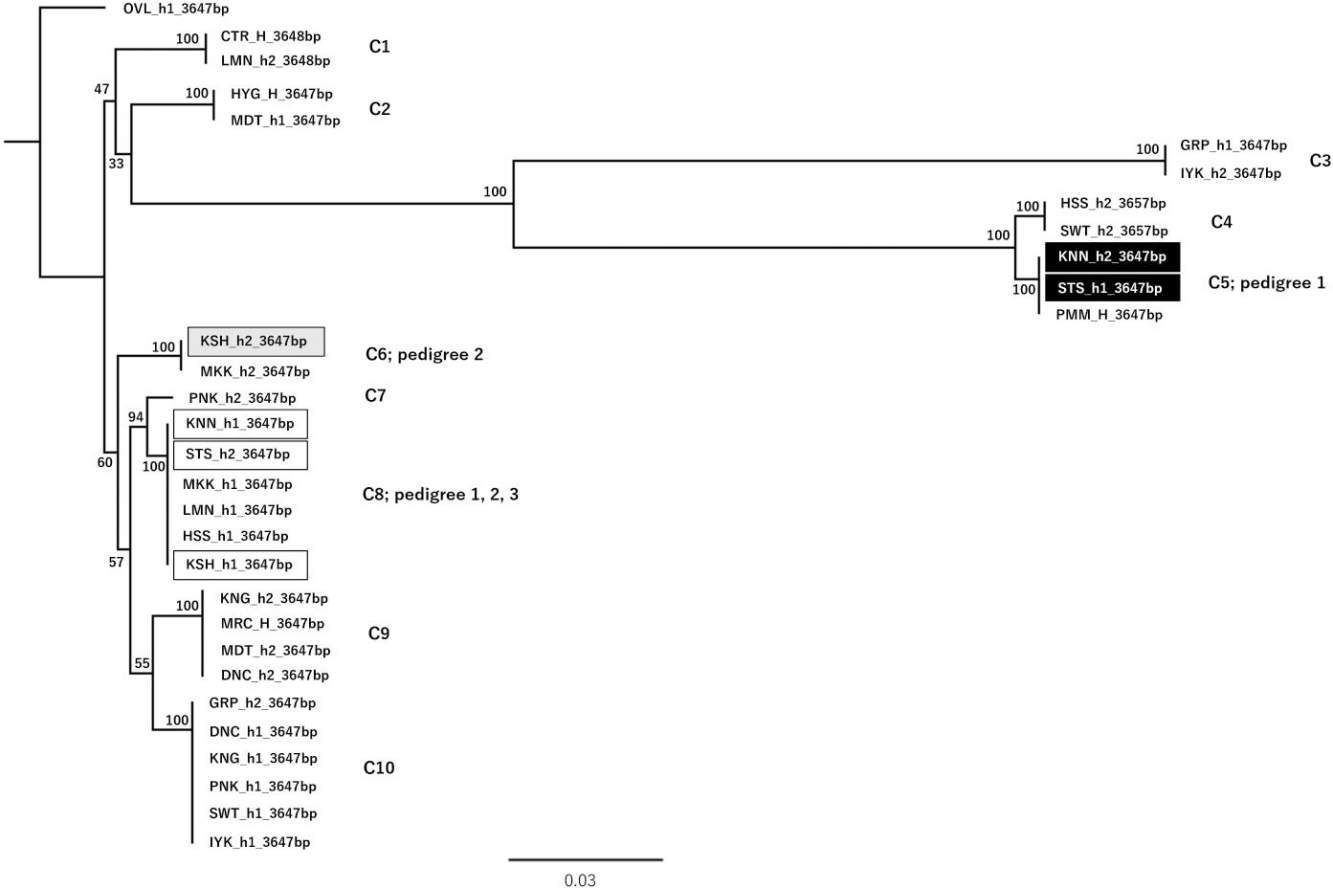
Phylogenetic tree of AlleleMiner-reconstructed allele sequences for *CUN7G148100.t1* in 18 citrus cultivars. The tree is provided to visualize allele sharing and allele transmission among cultivars; it is not intended for quantitative comparison of polymorphism levels or formal phylogenetic inference. The tree topology was consistent with the three known pedigree relationships (1 to 3) described in Section 2.5.1 and summarized in the pedigree diagram: (1) STS is an offspring of KSH × KNN; (2) MKK is a spontaneous mutant of KSH; and (3) KNN is a progeny of KSH. These relationships (1 to 3) were supported by the clustering patterns in the allele tree. Abbreviations: STS, Satsuma mandarin; KSH, Kishu mandarin; KNN, Kunenbo mandarin; PMM, pummelo; HSS, Hassaku; GRP, grapefruit; SWT, sweet orange; HYG, Hyuganatsu; IYK, Iyokan; PNK, Ponkan; DNC, Dancy tangerine; KNG, King mandarin; MRC, Murcott tangor; MDT, Mediterranean mandarin; MKK, Mukaku Kishu; LMN, lemon; CTR, citron; OVL, oval kumquat. Oval kumquat was used as an outgroup to root the tree.h1 and h2 denote haploid allele sequence outputs, and H denotes a homozygous output (a single haploid allele sequence) by AlleleMiner. The numbers starting with C next to each clade indicate the clade number.

**Fig. 7.**
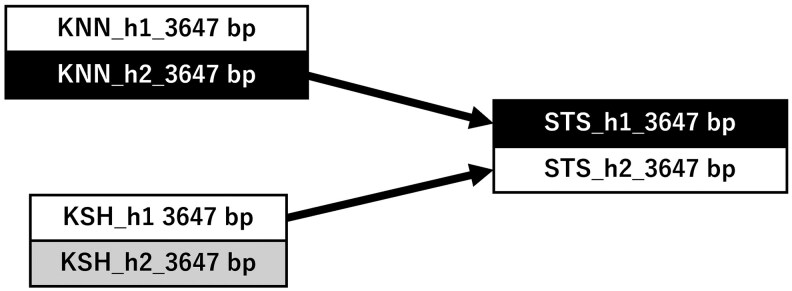
Allelic transmission pattern of *CUN7G148100.t1* alleles reconstructed AlleleMiner. Allele sharing indicated transmission consistent with the established pedigree: KNN_h2 was recovered in STS_h1 and KSH_h1 was recovered in STS_h2. h1 and h2 denote haploid allele sequences output by AlleleMiner. STS is a progeny of KSH × KNN. Abbreviations: STS, Satsuma mandarin; KSH, Kishu mandarin; KNN, Kunenbo mandarin.

All 18 cultivars yielded successfully phased alleles, forming a phylogenetic tree with 10 clades (Fig. 6, h1 and h2 denote haploid allele sequences output and H denotes a homozygous output by AlleleMiner). This tree is shown as a qualitative visualization to illustrate allele sharing among cultivars and pedigree-consistent allele transmission, rather than as a quantitative assessment of polymorphism levels or a formal phylogenetic inference. In clade 5, STS_h1 (3,647 bp) clustered with KNN_h2 (3,647 bp), while in clade 8, STS_h2 (3,647bp) was grouped with KSH_h1 (3,647 bp). Within these clades, allele lengths and pairwise distances were identical, confirming sequence identity (Table 2). Exact allele transmissions were observed: KNN_h2 corresponded to STS_h1 and KSH_h1 to STS_h2 (Fig. 7, Pedigree 1). In clade 6, KSH_h2 (3,647 bp) clustered with MKK_h2 (3,647 bp), and in clade 8, MKK_h1 (3,647 bp) grouped with KSH_h1 (3,647 bp), again with identical allele lengths and pairwise distances (Table 2, Pedigree 2).

**Table 2.** Pairwise distances among AlleleMiner-reconstructed alleles of four cultivars for *CUN7G148100.t1*.

	KNN_h2	STS_h1	KSH_h2	MKK_h2	KNN_h1	STS_h2	MKK_h1	KSH_h1
KNN_h2	…	0	703	703	820	820	820	820
STS_h1	0	…	703	703	820	820	820	820
KSH_h2	703	703	…	0	424	424	424	424
MKK_h2	703	703	0	…	424	424	424	424
KNN_h1	820	820	424	424	…	0	0	0
STS_h2	820	820	424	424	0	…	0	0
MKK_h1	820	820	424	424	0	0	…	0
KSH_h1	820	820	424	424	0	0	0	…

STS is a KSH × KNN hybrid, whereas MKK is a spontaneous mutant of KSH. h1 and h2 denote haploid allele sequences output by AlleleMiner.

Abbreviations: KNN, Kunenbo mandarin; KSH, Kishu mandarin; STS, Satsuma mandarin; MKK, Mukaku Kishu.

**Fig. 8.**
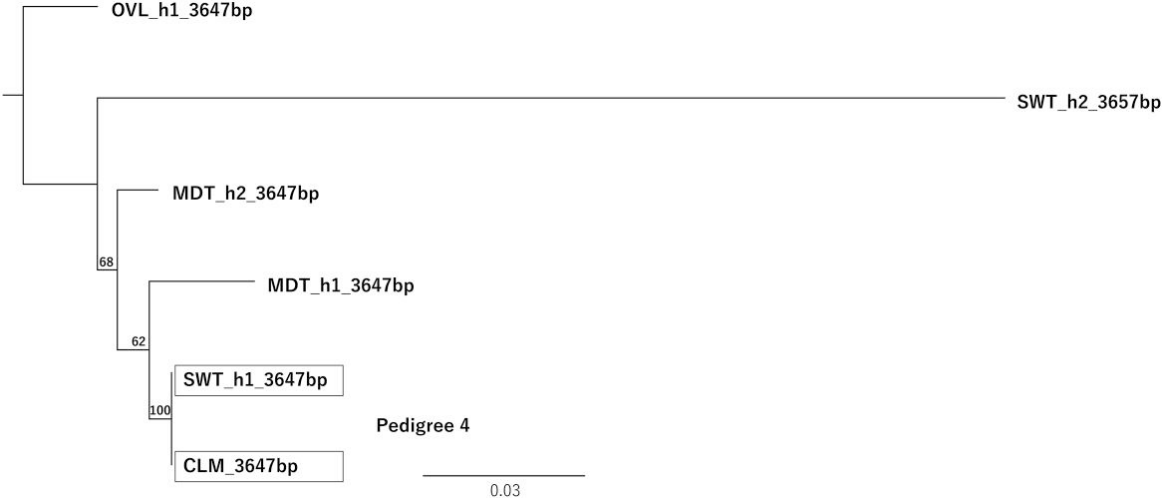
Phylogenetic tree of AlleleMiner-reconstructed allele sequences for *CUN7G148100.t1* in CLM, MDT, and SWT. The tree is provided to visualize allele sharing and allele transmission among cultivars; it is not intended for quantitative comparison of polymorphism levels or formal phylogenetic inference. The tree supports the known pedigree relationship: clementine (CLM) is a progeny of MDT × SWT, and SWT_h1 clusters with CLM sequence. The CLM allele sequence was retrieved from the clementine reference genome and presumed to represent a homologous locus. Abbreviations: CLM, clementine; MDT, Mediterranean mandarin; SWT, sweet orange. h1/h2 denotes the AlleleMiner haploid allele output.

Pedigree 3: In clade 8, KNN_h1 (3,647 bp) was shared with KSH_h1 (3,647 bp), and their pairwise distances were zero (Table 2). Pedigree 4: SWT_h1 (3,647 bp) also showed identical allele lengths and pairwise distances of zero with CLM (Fig. 8). The alignment of allelic sequences used for this phylogenetic analysis is provided in Figure S2.

#### 3.5.2. Pedigree-consistent allele transmission at CUN2G259500.t1 and detection of heterozygous structural variation consistent

Phased alleles were obtained for all 18 cultivars. The phylogenetic tree (Figure S3a) comprised 17 clades. Pedigree 1: In clade 17, STS_H (9,314 bp) clustered with KSH_h2 (9,314 bp) and KNN_h2 (9,3134 bp). Allele lengths and pairwise distances within this clade were identical. Exact transmissions were observed KNN_h1 corresponding to STS_H and KSH_h2 to STS_H (Figure S4a). Pedigree 2: In clade 14, KSH_h1 (9,315 bp) clustered with MKK_h1 (9,316 bp), while in clade 17, MKK_h2 (9,314 bp) clustered with KSH_h2 (9,314 bp). Allele lengths and pairwise distances among sequences in these clades were ≤1.

**Fig. 9.**
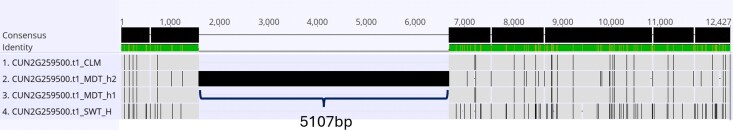
Alignment of AlleleMiner-reconstructed *CUN2G259500.t1* alleles showing a 5,107-bp insertion in one MDT allele. Multiple alignments of the *CUN2G259500.t1* gene region comparing the clementine reference allele (CLM; extracted from the reference genome) with AlleleMiner outputs from MDT and SWT. A 5,107-bp insertion was present in MDT_h2, whereas MDT_h1, SWT_H, and CLM lacked this insertion. The CLM reference allele was identical to MDT_h1 in the aligned region, indicating that the MDT allele carrying the insertion was not represented in the CLM reference sequence. Because CLM is a hybrid of SWT × MDT, this pattern is consistent with CLM carrying an MDT_h1-like allele and a distinct SWT-derived allele (not captured by haploid reference sequence). Abbreviations: CLM, clementine; MDT, Mediterranean mandarin; SWT, sweet orange. h1/h2 denote haploid allele sequence outputs, and H denotes a homozygous output (single allele) by AlleleMiner.

Pedigree 3: In clade 17, KNN_h2 (9,314 bp) was shared with KSH_h2 (9,314 bp), with identical allele lengths and pairwise distances. Pedigree 4: MDT_h1 (9,314 bp) clustered with CLM (9,314 bp) (Figure S5a). Allele lengths and pairwise distances within this clade were identical. Notably, AlleleMiner phasing of MDT revealed that MDT_h2 contained a 5,107 bp insertion relative to MDT_h1. To examine transmission of this insertion in CLM that is the MDT × SWT pedigree, we aligned the phased allele sequences from MDT (MDT_h1 and MDT_h2) and SWT (SWT_H) with the orthologous CLM sequence retrieved from Ccl v1.0, and found that the insertion was present only in MDT_h2 (Fig. 9), whereas CLM was identical to MDT_h1 (Fig. 9). Because the CLM sequence was retrieved from Ccl v1.0 (ie, representing a single haploid allele), its identity to MDT_h1 indicates that CLM inherited the non-insertion MDT allele (MDT_h1). The insertion in MDT_h2 showed high similarity to a *Vitis vinifera* retrovirus-related *Pol* polyprotein (GenBank accession XM_010662957) from transposon *TNT 1-94*,^51^ suggesting an origin from an LTR retrotransposon-like element. This finding demonstrates the ability of AlleleMiner to detect large insertions.

#### 3.5.3. Pedigree-consistent allele transmission at CUN1G201600.t1

Phased alleles were obtained for 17 cultivars, excluding GRP. The phylogenetic tree (Figure S3b) contained 13 clades. Pedigree 1: In clade 8, STS_h1 (4,528 bp) clustered with KNN_h1 (4,528 bp), and in clade 11, STS_h2 (4,528 bp) clustered with KSH_h2 (4,528 bp). Allele lengths and pairwise distances among sequences within these clades were identical. Exact allele transmissions were observed KNN_h1 corresponding to STS_h1 and KSH_h2 corresponding to STS_h2 (Figure S4b). Pedigree 2: In clade 11, KSH_h2 (4,528 bp) clustered with MKK_h1 (4,528 bp), and in clade 13, MKK_h2 (4,529 bp) clustered with KSH_h1 (4,529 bp). Allele lengths and pairwise distances within these clades were identical. Pedigree 3: In clade 13, KNN_h2 (4,529 bp) showed identical lengths and pairwise distances with KSH_h1.

Pedigree 4: SWT_h1 (4,481 bp) clustered with CLM (4,481 bp) (Figure S5b), and their allele lengths and pairwise distances were also identical.

### 3.6. Allele phasing of the *CCD4* gene family using AlleleMiner

Carotenoid cleavage dioxygenase 4 (*CCD4*) represents a multigene family in citrus comprising five known members: *CCD4a*, *CCD4b1*, *CCD4b2*, *CCD4c*, and *CCD4*.^47–50^ Because of their high sequence similarity, duplicated genes often pose challenges for phasing pipelines, as misassemble or allele switching can occur when paralogous reads are merged incorrectly. To examine this issue, we assessed whether AlleleMiner could accurately phase duplicated genes using the *CCD4* subfamily in three closely related cultivars (STS, KSH, and KNN).

**Fig. 10.**
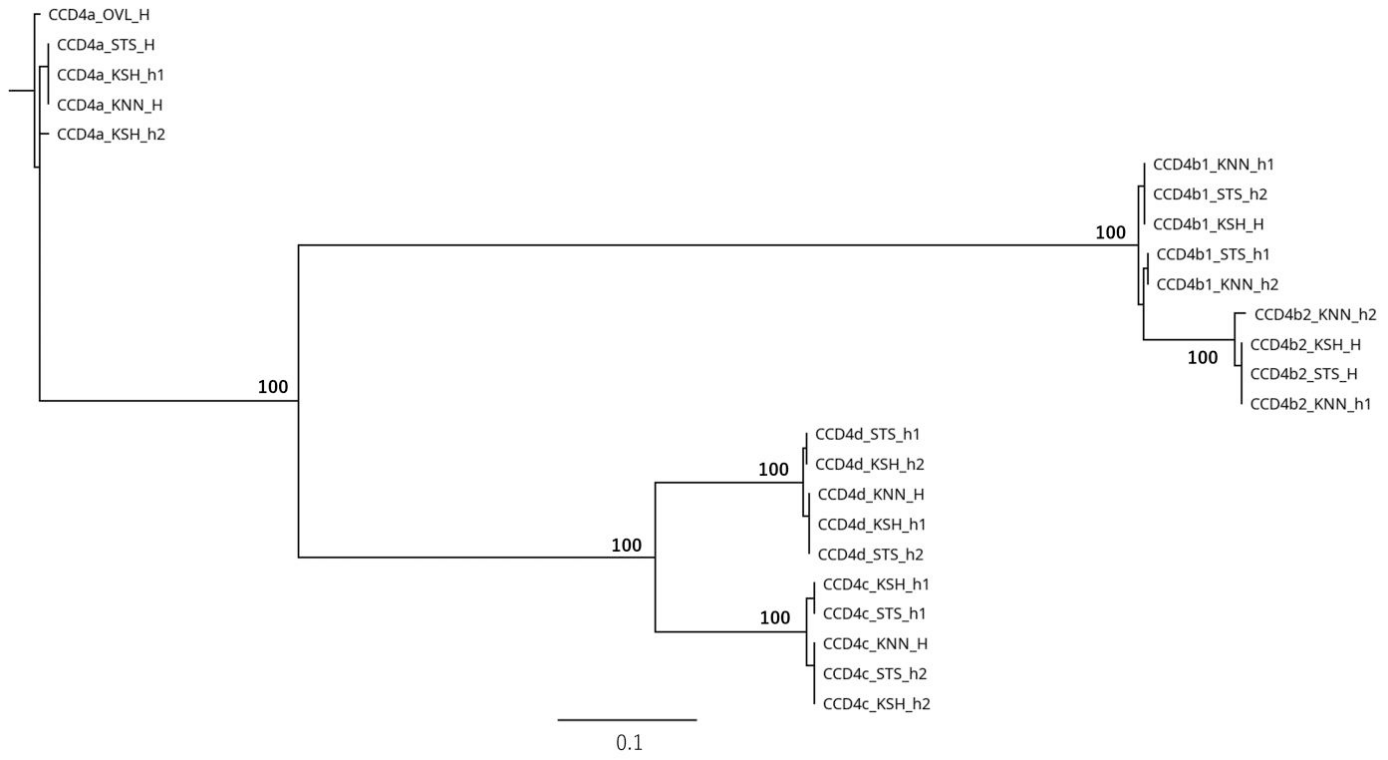
Phylogenetic relationships among STS, KSH, and KNN within the *CCD4* gene subfamily inferred from AlleleMiner-reconstructed alleles. The tree is provided to visualize allele sharing and allele transmission among cultivars; it is not intended for quantitative comparison of polymorphism levels or formal phylogenetic inference. Phylogenetic tree of allele sequences for five *CCD4* paralogs (*CCD4a*, *CCD4b1*, *CCD4b2*, *CCD4c*, and *CCD4d*) reconstructed by AlleleMiner from three closely related cultivars (STS, KSH, and KNN). The tip labels identify the alleles (gene name, cultivar code, and allele type). Alleles clustered by paralog identity rather than by cultivar origin, indicating that AlleleMiner distinguishes highly similar duplicated loci without merging cross-paralog reads. Bootstrap values are shown at the major nodes. Within each gene-specific clade, heterozygous loci yielded two alleles (h1 and h2), whereas homozygous loci produced a single allele (H). Abbreviations: STS, Satsuma mandarin; KSH, Kishu mandarin; KNN, Kunenbo mandarin; h1/h2, AlleleMiner haploid allele outputs (haploid 1/haploid 2); H, homozygous output (single allele). STS is a progeny of KSH × KNN.

AlleleMiner successfully reconstructed alleles for all five *CCD4* genes across the three cultivars. Phylogenetic analysis (Fig. 10) showed that alleles clustered primarily by gene identity rather than cultivar origin, supported by high bootstrap values among clades. For instance, *CCD4a* alleles from STS, KSH, and KNN formed a single, well-supported clade distinct from those of *CCD4b1* and *CCD4b2*. These results indicate that AlleleMiner accurately distinguished paralogous copies with high fidelity.

Within each gene-specific clade, heterozygous loci produced two distinct alleles (h1 and h2), whereas homozygous loci yielded a single allele (H), consistent with the patterns observed in single-copy genes. For example, in *CCD4b1*, STS_h1 and STS_h2 clustered separately with KSH_H and KNN_h2, reflecting known pedigree relationships. Similarly, *CCD4c* and *CCD4d* exhibited allele segregation consistent with allele transmission, confirming that paralogous gene copies were resolved without cross-cultivar misassignment.

Together, these findings demonstrate that AlleleMiner extends beyond single-copy loci to phase members of complex gene families reliably. This capability is particularly relevant to citrus carotenoid metabolism, where functional diversification among *CCD4* paralogs influences carotenoid composition. The successful phasing of *CCD4* alleles across STS, KSH, and KNN underscores the broad applicability of AlleleMiner to duplicated and functionally redundant gene families.

## 4. Discussion

A fundamental distinction between AlleleMiner and conventional variant calling-based approaches lies in the representation of allelic variation. Variant calling frameworks define genetic diversity as discrete differences relative to a reference genome, such as SNPs, small indels, or structural variants, each anchored to reference coordinates. Although this representation is effective for population-scale analyses, it constrains allelic diversity to reference-dependent variant feature calls and does not directly yield independent allele sequences that can be reused as complete genetic units.

AlleleMiner was designed to overcome this limitation by decoupling allele reconstruction from reference-based coordinate systems. In this framework, the reference genome is used exclusively to identify TGRS and select homologous long reads. All subsequent processing was performed through locus-wise *de novo* assembly, enabling the reconstruction of phased allele sequences, reference-independent allele sequences rather than reference-anchored variant features.

AlleleMiner is not intended to replace conventional variant-calling approaches. Variant calling remains highly effective for applications such as population genetics, genome-wide association studies, and large-scale genotyping, where reference-anchored coordinates provide a common framework for integrating variations across numerous samples. However, for gene-centred analyses that require direct comparison, clustering, or pedigree-based tracking of complete alleles, representing variation solely as a collection of variant features can be limiting.

By reconstructing alleles as continuous sequences, AlleleMiner complements variant calling by representing genetic variation that is more naturally aligned with functional interpretation, inheritance analysis, and breeding-oriented applications. In this sense, AlleleMiner and variant calling approaches serve distinct but complementary roles, differing primarily in how allelic diversity is encoded and used, rather than in their ability to detect variation.

AlleleMiner provides an automated and efficient pipeline for phasing diploid gene sequences using PacBio HiFi reads. By extracting long reads that span target genes and performing HiFi-based *de novo* assembly, the pipeline reconstructs alleles and distinguishes between homozygous and heterozygous loci.

The reference genome is used only to extract reads and locate gene regions, after which assembly proceeds independently of reference similarity. Through this reference-minimized design, AlleleMiner enables robust and unbiased allele phasing across cultivars with diverse genetic backgrounds, overcoming a key limitation of conventional reference-guided analyses. AlleleMiner represents a major advance in minimizing reference dependence for the discovery of alleles in heterozygous crops.

AlleleMiner is intended as a complementary approach to whole-genome assembly, enabling standardized allele-level comparisons at defined gene loci across numerous cultivars, particularly in crops for which generating phased chromosome-scale assemblies for all lines remains impractical. Notably, even when high-quality phased, chromosome-scale assemblies are available, deriving allele sequences that are directly comparable at the gene level across many cultivars is not necessarily straightforward. Phased assemblies are generated in sample-specific coordinate systems, and gene models and boundaries are frequently inferred separately for each assembly. Consequently, cross-cultivar allele comparisons often require additional steps (eg, whole-genome alignment, orthology assignment, and consistent boundary definition), which can vary across pipelines and thereby introduce methodological heterogeneity. AlleleMiner addresses this comparability challenge by using a single reference genome and its GFF3 annotation only as an anchor to locate target regions and define gene boundaries, while reconstructing allele sequences *de novo* from locus-specific read sets. This locus-centric, read-supported strategy provides locus-level quality control and may reduce the impact of locus-scale artefacts that can persist even in phased assemblies (eg, haplotype switches or missing haplotigs), particularly in highly heterozygous genomes such as those of citrus cultivars. Accordingly, AlleleMiner is positioned as a complementary approach that facilitates standardized allele sequence generation across large cultivar panels and can serve as an independent, read-supported layer to validate or refine allele sequences derived from genome assemblies.

### 4.1. Overall performance and robustness of AlleleMiner

This study evaluated the performance of AlleleMiner on real-world data comprising 18 cultivars and 1,409 loci, confirming a high average phasing success rate of 91.5% (Table 1). As this study focused on diploid organisms, phasing success was defined as the output of either one or two phased allele sequences per locus. A single sequence was classified as homozygous, and two sequences were classified as heterozygous. Based on this operational definition, the evaluation showed that AlleleMiner operated stably and exhibited consistent behaviour independent of specific genetic backgrounds, even under conditions where heterozygosity varied greatly between cultivars. Analysis proceeded without failure from the nearly homozygous CTR to the highly heterozygous KSH, demonstrating the robustness of the pipeline design.

The success rate correlated more strongly with sequencing depth (*r* = 0.795) than with cultivar heterozygosity (Figure S1), suggesting that a high success rate can be achieved regardless of the degree of genetic diversity, provided that sufficient read coverage is obtained. This trend is consistent with the design philosophy of AlleleMiner, which reconstructs alleles based on *de novo* assembly using long reads that completely encompass the target gene rather than relying on simple mapping results to the reference genome.

When we summarized phasing failures by cultivar and by pipeline step (Table S2), 78.8% of all failures occurred at the *de novo* assembly stage. This pattern was particularly pronounced in cultivars with coverage ≤20×. Taken together, these results indicate that the primary cause of AlleleMiner phasing failure is insufficient sequencing depth, leading to unsuccessful *de novo* assembly. Based on the above results and Figure S1, the success rate of AlleleMiner primarily depends on coverage. Within the scope of this study, no systematic effects attributable to differences among cultivars were observed. Therefore, under conditions where sufficient coverage is ensured, AlleleMiner demonstrates stable performance in real-world data analysis and can be considered a robust pipeline for the practical implementation of locus-level allele phasing.

### 4.2. Sequencing coverage requirements for reliable allele phasing

We evaluated the impact of sequence coverage on AlleleMiner output using both real and simulated data. The results showed that, focusing on the total output count, the number of outputs tended to saturate early, even at relatively low-coverage levels. For real data, this saturation occurred at approximately 20× (Fig. 3), and for the low- and high-frequency variant model in the simulated data, the total output count also plateaued at 20× (Fig. 4). These results indicate that AlleleMiner can produce practical outputs, in terms of recovering a certain number of allele sequences, even at approximately 20 × coverage.

However, a distinct trend was observed when focusing on the heterozygote outputs. In real data, at 30× coverage, both the total output count and heterozygous output reached approximately 99% of the values observed at 42.9× coverage (Fig. 3). In the simulated data, the heterozygous output reached its maximum or near-maximum values at 30× or higher for both the low- and high-frequency variant models (Fig. 4). These results suggest that a higher coverage than the point at which the total output saturates is necessary for the stable recovery of heterozygous alleles.

The discrepancy in which the total output saturates at approximately 20×, whereas the heterozygous output peaks at 30×, indicates that under low-coverage conditions, so-called allele drop may occur, in which only one allele is reconstructed whereas the other is lost. Specifically, in heterozygotes, if sufficient reads are not distributed to both alleles, *de novo* assembly-based facing is incomplete. Therefore, relying solely on the total output count as an indicator risk overlooking such incomplete facing.

AlleleMiner achieved consistent results for allele facing at approximately 20× coverage when evaluated by total output, but a coverage of 30× or higher is desirable for stable and complete recovery of heterozygous alleles. Conversely, coverage exceeding 30× did not yield considerable improvements in either the total output or heterozygous output and did not substantially alter AlleleMiner’s analysis results. Therefore, for allele phasing at the locus level in citrus, a HiFi coverage of approximately 30× is considered a practical benchmark that balances analytical accuracy and cost.

### 4.3. Allele reconstruction performance evaluated using simulated and real datasets

We verified the behaviour of allele reconstruction using AlleleMiner with both simulated and real data sets. In the simulated data analysis, the output of AlleleMiner showed a high perfect match rate for allele sequences derived from the haplotypes (HAP_AL and HAP_AH) used as the reference genome and reference GFF3. Specifically, 81.7% of the reconstruction sequences by AlleleMiner matched ALLELE_AL perfectly under the low-frequency variant model, and 98.0% matched ALLEL_AH perfectly under the high-frequency variant model. This confirmed that alleles consistent with the provided reference genome structure were reconstructed with high precision.

The perfect match rate with the allele sequence derived from the other haplotype (HAP_B), which was not used as a reference, were at 80.6% in the low-frequency variant model, and at just under 70% in the high-frequency variant models. The trend that the HAP_BH allele's perfect match rate is lower than that of the HAP_AH is explained by the fact that ALLELE_AH, which are structurally consistent with the reference HAP_AH, are reconstructed efficiently, whereas ALLELE_BH with structural differences from the reference do not achieve a perfect match in a certain percentage of cases. This trend is stronger in the high-frequency variant model, which includes many variants between HAP_AH and HAP_BH. Whereas the number of loci where both alleles were simultaneously reconstructed as perfect matches was just under 70% of the total, in both low- and high-frequency variant models.

This perfect match rate of approximately 70% is not a phenomenon specific to virtual data but was also reproduced in real data analysis. In a verification using CLM_BUSCO_GENES as a substitute reference for the parental allele, perfect matches between AlleleMiner output and CLM_BUSCO_GENES were confirmed in 70.4% of the 1,159 loci. This value aligns well with the perfect match rate for the allele side not used as a reference in the simulated data, indicating that AlleleMiner's allele reconstruction behaviour is consistent and independent of the data type.

Under these conditions, this study defined successful AlleleMiner allele reconstruction as loci at which both allele sequences showed a “perfect-match”, which was observed for approximately 70% of loci. This definition applies a stringent, perfect-match criterion; partially reconstructed alleles or those showing high similarity but incomplete reconstruction, although such reconstructions may still be biologically meaningful in downstream analyses, were intentionally excluded from this perfect-match rate. Reconstruction performance was evaluated under the constraints using a single reference genome to locate target regions and GFF3-based annotation to define gene boundaries.

In real data analysis, a tendency was observed for the perfect match rate to decrease with increasing gene sequence length. This is thought to occur because longer genes are more likely to contain structural variations or insertions/deletions, making it more difficult to achieve a perfect match with the reference sequence. Conversely, perfect matches were confirmed in approximately 80% of loci under 6,000 bp, demonstrating that AlleleMiner possesses high reconstruction performance at practical gene lengths.

Based on these results, we can conclude that AlleleMiner accurately recovers alleles consistent with the reference genome while also reconstructing alleles with structural differences from the reference with approximately a 70% probability of being perfect matches. This conclusion is supported by both virtual and real data.

### 4.4. Verification of AlleleMiner output based on allele transmission in pedigree

Validation using three representative single-copy genes demonstrated that AlleleMiner reliably reconstructs alleles in a manner consistent with allele transmission inferred from known pedigree relationships. The known parent–offspring relationships were accurately reflected in the phylogenetic trees, and the allele lengths differed by no more than one base per kbp, consistent with the 99.5%–99.9% accuracy range of HiFi reads.

In these analyses, alleles shared among related cultivars clustered with zero or near-zero pairwise distances, indicating identical or near-identical sequences and supporting pedigree-consistent allele transmission inferred from AlleleMiner outputs. Minor differences of one or a few bases were tolerated in this evaluation, reflecting both the intrinsic error of HiFi reads and the practical resolution required for allele-level reconstruction.

The detection of a 5.1 kbp transposon insertion in MDT_h2 demonstrates the advantage of long reads in identifying structural variants that are typically missed by short-read methods.

This large insertion was detected within a phased allele and correctly distinguished from the alternative allele at the same locus, illustrating that AlleleMiner can recover substantial heterozygous insertions while preserving allele transmission patterns across related cultivars.

The ability to detect such large indels in a pedigree-consistent manner highlights the utility of AlleleMiner for capturing structural variations at gene loci beyond SNPs.

The successful phasing of the duplicated *CCD4* gene family further demonstrated that AlleleMiner effectively resolves paralogous loci. Despite the high sequence similarity among *CCD4* family genes, alleles were primarily clustered by gene identity rather than by cluster origin, and heterozygous and homozygous states were consistently distinguished within each gene-specific clade. These results indicate that AlleleMiner can accurately reconstruct alleles for single-copy genes and complex gene families, in which paralogous sequences confound real-based phasing approaches.

Together, these findings indicate that the pipeline captures both fine-scale polymorphisms and large structural variants while validating pedigree-consistent allele transmission at the representative loci. As summarized in Table S2, a comparison of allele sequence lengths, Jaccard coefficients, and MinHash values provides an additional quantitative layer for interpreting heterozygosity and allele transmission inferred by AlleleMiner. For example, when insertions, such as those observed in *CUN2G259500.t1*, occur heterozygosity, substantial differences in allele length can reveal the presence of structural variations. The Jaccard coefficient, which is equal to 1 for identical alleles, serves as an index of sequence similarity between heteroallelic pairs. In contrast, the MinHash value acts as a compact signature that is identical only when the two allele sequences are the same. Accordingly, comparing MinHash values among cultivars enables the rapid identification of shared or unique alleles with no alignment, facilitating the large-scale assessment of allele transmission patterns across pedigrees. In addition, MinHash signatures obtained from multiple independent AlleleMiner runs can be aggregated into an allele “sketch” database and compared across datasets to detect recurrent or highly similar allele sequences, enabling scalable downstream screening of shared alleles and candidate transmission links before performing alignments for confirmation.

### 4.5. Scope and extensibility of AlleleMiner

Regarding sequencing depth, whereas the output saturates at approximately 20× when using the total output count as the sole metric, both real and simulated data demonstrated that approximately 30× or higher HiFi coverage is required for the stable recovery of heterozygous alleles. Under 20× conditions, cases in which only one allele is reconstructed persist, potentially leading to the so-called allele drop. Therefore, approximately 30× can be recommended as a practical lower limit for reliable allele detection.

Regarding the allele reconstruction success rate, approximately 70% of loci were reconstructed with both alleles perfectly matched. This value aligns with the non-reference allele matching rate observed in virtual data analysis and real data validation using the CLM_BUSCO_GENES (70.4%), indicating that the behaviour of AlleleMiner is consistent and independent of the data type. In this study, we defined a complete-match rate of approximately 70% as a successful allele reconstruction.

Gene length is a critical factor affecting the success rate. TGRS under 6,000 bp achieved approximately 80% perfect match rates, whereas match rates decreased with increasing gene length. This is likely because AlleleMiner is designed to assemble based on reads spanning the entire TGRS, making it more difficult to secure sufficient reads for longer genes. In the current implementation, TGRS under 6,000 bp is considered the most suitable application range. As a future improvement to address this limitation, dividing TGRS exceeding 6,000 bp into multiple subregions for allele reconstruction, followed by sequence integration, is considered effective. This segmentation and integration approach has the potential to mitigate the problem of insufficient read coverage at long gene loci and expand the range of applications.

In the current pipeline, Hifiasm is used as the primary assembler, with Flye as a fallback assembler when Hifiasm fails to produce valid phased contigs. As shown in Fig. 4b and 4d, the two assemblers are used complementarily under certain conditions, and the combined fallback strategy is crucial for ensuring the robustness of AlleleMiner and increasing the success rate. However, an analysis of the simulated data under the high-variation model revealed a tendency in assembler usage. As shown in Fig. 4d, the assemblies generated by Flye accounted for a substantially larger fraction of the successful outputs under high-variation conditions. Similarly, in the analysis of 18 citrus cultivars (Section 2.5), only 14.3% of loci were successfully processed using Hifiasm alone, whereas 85.7% required Flye as a fallback assembler. These results indicate that Flye is more tolerant than Hifiasm for highly polymorphic loci. Accordingly, for datasets expected to contain high levels of allelic variation, configuring Flye as the primary assembler may yield higher allele recovery rates than the current configuration. Regarding the selection for the primary assembler, Flye should be implemented as the primary assembler in the next update.

Although AlleleMiner was implemented for diploid organisms in this study, its core algorithm is conceptually extensible to polyploids. For example, in triploids, allowing up to three phased contigs per locus during the allele extraction step enables the output of three allelic sequences. Similar extensions are possible for tetraploids and above, although additional algorithm development is required to identify multiple haplotypes and manage their combinations. This study first focused on establishing a robust diploid implementation.

AlleleMiner is designed for allele-level phasing at predefined gene loci and does not analyse genome-wide structural variations, such as inversions or translocations. The hash values and Jaccard distances output by AlleleMiner are designed for allele acquisition and a lightweight similarity assessment. However, structural interpretation of alleles should be performed using appropriate downstream annotation tools.

### 4.6. Conclusion

This study presents AlleleMiner, a pipeline for determining allele facing at diploid loci using PacBio HiFi reads and systematically validated its utility across 1,409 loci in 18 citrus cultivars. AlleleMiner reconstructs allele sequences through *de novo* assembly, minimizing reference genome dependency and enabling stable recovery of heterozygous alleles at approximately 30× HiFi coverage. Furthermore, validation using simulated data and CLM_BUSCO_GENES showed that complete-match-based allele reconstruction was achieved at approximately 70% of loci, improving to approximately 80% for loci under 6,000 bp. Including validation using pedigree information and duplicated gene families, AlleleMiner provides a practical foundation for gene-centred allele discovery in heterozygous crops by complementing reference-anchored variant feature–based analyses with direct reconstruction of independent allele sequences.

## Supplementary Material

dsag004_Supplementary_Data

## Data Availability

The datasets supporting this article are available in the DDBJ DRA repository (https://www.ddbj.nig.ac.jp/dra/index-e.html) under BioProject PRJDB15866 and PRJDB15974. Moreover, AlleleMiner source code is freely available from the GitHub repository (https://github.com/YukinariKiryu/AlleleMiner).
