# Supplementary material for: AlleleMiner: a long-read pipeline for gene-wise de novo allele phasing and variant detection in diploid citrus cultivars
Source: DNA Res. 2026 Mar 3;33(2):dsag004. doi: 10.1093/dnares/dsag004 (PMC13011809; doi:10.1093/dnares/dsag004)
Supplement: dsag004_Supplementary_Data [file dsag004_supplementary_data.zip › Kiryu_Supplementary_Fig_S2_260216.pdf]

Fig. S2 Multiple alignment of 18 cultivars and clementine allele sequences for CUN7G148100.t1 obtained by AlleleMiner analysis.
Citrus hybrids in breeding have been developed by crossing the cultivars derived from 13 founders among the 18 cultivars listed below, and therefor each cultivar possesses the alleles listed below at each respective gene locus.
Abbreviations: KSH, Kishu mandarin; KNN, Kunenbo mandarin; PMM, pummelo; HSS, Hassaku; GRP, grapefruit; SWT, sweet orange; HYG, Hyuganatsu; IYK, Iyokan; PNK, Ponkan; DNC, Dancy tangerine; KNG, King mandarin; MRC, Murcott tangor; MDT, Mediterranean mandarin. The above are the 13 founders. STS, Satsuma mandarin; MKK, Mukaku Kishu; LMN, lemon; CTR, citron; OVL, oval kumquat.

```
Consensus      AATAATTCATAATATTCATGTGTACGGTATCATGTTCAACTTGTTAAATTGATATGATA-    59
OVL_h1_3647bp ...........G.............A.C...........................A.G.-    59
CTR_H_3648bp  .........................................................G.-    59
LMN_h2_3648bp .........................................................G.-    59
HYG_H_3647bp  ----------------------------------------------------------.-     1
MDT_h1_3647bp ----------------------------------------------------------.-     1
GRP_h1_3647bp ----------------------------------AA...AAGAGG....GCT..AAG..A    26
IYK_h2_3647bp ----------------------------------AA...AAGAGG....GCT..AAG..A    26
HSS_h2_3657bp ----------------------------------..-TT...T..CTT..TC.T.TT.G-    24
SWT_h2_3657bp ----------------------------------..-TT...T..CTT..TC.T.TT.G-    24
KNN_h2_3647bp ---------------------------------------...T..CTT..TC.T.TT.G-    20
STS_h1_3647bp ---------------------------------------...T..CTT..TC.T.TT.G-    20
PMM_H_3647bp  ---------------------------------------...T..CTT..TC.T.TT.G-    20
KSH_h2_3647bp ------------------------------------------.................-    17
MKK_h2_3647bp ------------------------------------------.................-    17
PNK_h2_3647bp ----------------------------------.........................-    25
KNN_h1_3647bp ------------------------------------------------------------
STS_h2_3647bp ------------------------------------------------------------
MKK_h1_3647bp ------------------------------------------------------------
LMN_h1_3647bp ------------------------------------------------------------
HSS_h1_3647bp ------------------------------------------------------------
KSH_h1_3647bp ------------------------------------------------------------
KNG_h2_3647bp -----------------------------------...................C....-    24
MRC_H_3647bp  ----------------------------------....................C....-    25
MDT_h2_3647bp ----------------------------------....................C....-    25
DNC_h2_3647bp ----------------------------------....................C....-    25
GRP_h2_3647bp ------------------------------------------------------------
DNC_h1_3647bp ------------------------------------------------------------
KNG_h1_3647bp ------------------------------------------------------------
PNK_h1_3647bp ------------------------------------------------------------
SWT_h1_3647bp ------------------------------------------------------------
IYK_h1_3647bp ------------------------------------------------------------
CLM_3647bp    ------------------------------------------------------------

Consensus     -------------------GGTCTGGAGTTCAAGT---------TCCGCTCATTAATTGA    91
OVL_h1_3647bp -------------------...T............---------.....C........A.    91
CTR_H_3648bp  -------------------................---------................    91
LMN_h2_3648bp -------------------................---------................    91
HYG_H_3647bp  -------------------.....A..........---------................    33
MDT_h1_3647bp -------------------.....A..........---------................    33
GRP_h1_3647bp AGTGGGGGTGGAGCCAAGA.AA.AC.C.CAA..CGTCAGCACAAA.T...GT..CTC.C.    86
IYK_h2_3647bp AGTGGGGGTGGAGCCAAGA.AA.AC.C.CAA..CGTCAGCACAAA.T...GT..CTC.C.    86
HSS_h2_3657bp -------------------..GTAA.G.C.A.T.A---------.A.AA.GT.C...G--    54
SWT_h2_3657bp -------------------..GTAA.G.C.A.T.A---------.A.AA.GT.C...G--    54
KNN_h2_3647bp -------------------..GTAA.G.C.A.T.A---------.A.AA.GT.C...G--    50
STS_h1_3647bp -------------------..GTAA.G.C.A.T.A---------.A.AA.GT.C...G--    50
PMM_H_3647bp  -------------------..GTAA.G.C.A.T.A---------.A.AA.GT.C...G--    50
KSH_h2_3647bp -------------------................---------...C............    49
MKK_h2_3647bp -------------------................---------...C............    49
PNK_h2_3647bp -------------------................---------................    57
KNN_h1_3647bp ------------------------------------------------------------
STS_h2_3647bp ------------------------------------------------------------
MKK_h1_3647bp ------------------------------------------------------------
LMN_h1_3647bp ------------------------------------------------------------
HSS_h1_3647bp ------------------------------------------------------------
KSH_h1_3647bp ------------------------------------------------------------
```

```
KNG_h2_3647bp -------------------.A..............---------................    56
MRC_H_3647bp  -------------------.A..............---------................    57
MDT_h2_3647bp -------------------.A..............---------................    57
DNC_h2_3647bp -------------------.A..............---------................    57
GRP_h2_3647bp ------------------------------------------------------------
DNC_h1_3647bp ------------------------------------------------------------
KNG_h1_3647bp ------------------------------------------------------------
PNK_h1_3647bp ------------------------------------------------------------
SWT_h1_3647bp ------------------------------------------------------------
IYK_h1_3647bp ------------------------------------------------------------
CLM_3647bp    ------------------------------------------------------------

Consensus     AGTAATGC-AAACTTACCTAGTAT-TAGGA--------------CTTAAAGTCCACAAAC   135
OVL_h1_3647bp ........-...............-.....--------------....G...........   135
CTR_H_3648bp  ........-...............-.....--------------................   135
LMN_h2_3648bp ........-...............-.....--------------................   135
HYG_H_3647bp  ........-...............-.....--------------................    77
MDT_h1_3647bp ........-...............-.....--------------................    77
GRP_h1_3647bp G..TC.CT-C.G.C.T..GGACG.TA..C.TTTTCCATGTTCATG..C..TAAG.G...T   145
IYK_h2_3647bp G..TC.CT-C.G.C.T..GGACG.TA..C.TTTTCCATGTTCATG..C..TAAG.G...T   145
HSS_h2_3657bp ...G....T....G.CGGG.----------------------------------------    74
SWT_h2_3657bp ...G....T....G.CGGG.----------------------------------------    74
KNN_h2_3647bp ...G...TT....G.CGGA.----------------------------------------    70
STS_h1_3647bp ...G...TT....G.CGGA.----------------------------------------    70
PMM_H_3647bp  ...G...TT....G.CGGA.----------------------------------------    70
KSH_h2_3647bp ........-...............-.....--------------................    93
MKK_h2_3647bp ........-...............-.....--------------................    93
PNK_h2_3647bp ........-...............-.....--------------................   101
KNN_h1_3647bp ------------------------------------------------------------
STS_h2_3647bp ------------------------------------------------------------
MKK_h1_3647bp ------------------------------------------------------------
LMN_h1_3647bp ------------------------------------------------------------
HSS_h1_3647bp ------------------------------------------------------------
KSH_h1_3647bp ------------------------------------------------------------
KNG_h2_3647bp ........-...............-.....--------------................   100
MRC_H_3647bp  ........-...............-.....--------------................   101
MDT_h2_3647bp ........-...............-.....--------------................   101
DNC_h2_3647bp ........-...............-.....--------------................   101
GRP_h2_3647bp ------------------------------------------------------------
DNC_h1_3647bp ------------------------------------------------------------
KNG_h1_3647bp ------------------------------------------------------------
PNK_h1_3647bp ------------------------------------------------------------
SWT_h1_3647bp ------------------------------------------------------------
IYK_h1_3647bp ------------------------------------------------------------
CLM_3647bp    ------------------------------------------------------------

Consensus     TCAAGCCCTCGGGGCAATGCCCAATGATGGACGAAACCGGGGCTGACATTTTCTTATCAA   195
OVL_h1_3647bp ............................................................   195
CTR_H_3648bp  .......................................................G....   195
LMN_h2_3648bp .......................................................G....   195
HYG_H_3647bp  .......................................T...............G....   137
MDT_h1_3647bp .......................................T...............G....   137
GRP_h1_3647bp .T.TCGT.C.TTACA..A.TAA...A.AAT.T....AAA..A.AC.G.GAC..C..AT.T   205
IYK_h2_3647bp .T.TCGT.C.TTACA..A.TAA...A.AAT.T....AAA..A.AC.G.GAC..C..AT.T   205
HSS_h2_3657bp -----------A...T.A-GAG...CC..AGA.....G.CAA..TGTGC.G.TG.T...C   122
SWT_h2_3657bp -----------A...T.A-GAG...CC..AGA.....G.CAA..TGTGC.G.TG.T...C   122
KNN_h2_3647bp -----------A...T.A-GAG...CC..AGA.....G.CAA..TGTGC.G..G.T...C   118
STS_h1_3647bp -----------A...T.A-GAG...CC..AGA.....G.CAA..TGTGC.G..G.T...C   118
PMM_H_3647bp  -----------A...T.A-GAG...CC..AGA.....G.CAA..TGTGC.G..G.T...C   118
KSH_h2_3647bp ............................................................   153
MKK_h2_3647bp ............................................................   153
PNK_h2_3647bp ...........................G................................   161
KNN_h1_3647bp ------------------------------------------------------------
STS_h2_3647bp ------------------------------------------------------------
MKK_h1_3647bp ------------------------------------------------------------
LMN_h1_3647bp ------------------------------------------------------------
HSS_h1_3647bp ------------------------------------------------------------
KSH_h1_3647bp ------------------------------------------------------------
```

```
KNG_h2_3647bp ............................................................   160
MRC_H_3647bp  ............................................................   161
MDT_h2_3647bp ............................................................   161
DNC_h2_3647bp ............................................................   161
GRP_h2_3647bp ------------------------------------------------------------
DNC_h1_3647bp ------------------------------------------------------------
KNG_h1_3647bp ------------------------------------------------------------
PNK_h1_3647bp ------------------------------------------------------------
SWT_h1_3647bp ------------------------------------------------------------
IYK_h1_3647bp ------------------------------------------------------------
CLM_3647bp    ------------------------------------------------------------

Consensus     GATC-ATC-AGGGGCATCATCGCCACCGTTCATTTGATCATCACCAGCTA----------   243
OVL_h1_3647bp ....-...-.........................................----------   243
CTR_H_3648bp  ....-...-............A...........CC...............----------   243
LMN_h2_3648bp ....-...-............A...........CC...............----------   243
HYG_H_3647bp  ....-...-............A...........C....T...........----------   185
MDT_h1_3647bp ....-...-............A...........C....T...........----------   185
GRP_h1_3647bp TTGA-..A-....A..-..GA.ATC.TTAA.......AA.AA.GATA.A.----------   252
IYK_h2_3647bp TTGA-..A-....A..-..GA.ATC.TTAA.......AA.AA.GATA.A.----------   252
HSS_h2_3657bp .CGTGT..TC.T...CC..C.C..CA....A-CG.TC..T.TT.TTTT..ACTTAACGTT   181
SWT_h2_3657bp .CGTGT..TC.T...CC..C.C..CA....A-CG.TC..T.TT.TTTT..ACTTAACGTT   181
KNN_h2_3647bp .CGTGT..TC.T...CC..C.C..CA.T..A-CG.TC..T.TT.TTTT..ACTTAACGTT   177
STS_h1_3647bp .CGTGT..TC.T...CC..C.C..CA.T..A-CG.TC..T.TT.TTTT..ACTTAACGTT   177
PMM_H_3647bp  .CGTGT..TC.T...CC..C.C..CA.T..A-CG.TC..T.TT.TTTT..ACTTAACGTT   177
KSH_h2_3647bp ....-...-...........................G.............----------   201
MKK_h2_3647bp ....-...-...........................G.............----------   201
PNK_h2_3647bp ....-...-.........................................----------   209
KNN_h1_3647bp ------------------------------------------------------------
STS_h2_3647bp ------------------------------------------------------------
MKK_h1_3647bp ------------------------------------------------------------
LMN_h1_3647bp ------------------------------------------------------------
HSS_h1_3647bp ------------------------------------------------------------
KSH_h1_3647bp ------------------------------------------------------------
KNG_h2_3647bp ....-...-.........................................----------   208
MRC_H_3647bp  ....-...-.........................................----------   209
MDT_h2_3647bp ....-...-.........................................----------   209
DNC_h2_3647bp ....-...-.........................................----------   209
GRP_h2_3647bp ------------------------------------------------------------
DNC_h1_3647bp ------------------------------------------------------------
KNG_h1_3647bp ------------------------------------------------------------
PNK_h1_3647bp ------------------------------------------------------------
SWT_h1_3647bp ------------------------------------------------------------
IYK_h1_3647bp ------------------------------------------------------------
CLM_3647bp    ------------------------------------------------------------

Consensus     -----------------------TGTGAGCCAATATATCAAGCATAGTAAGCATCCCTAT   280
OVL_h1_3647bp -----------------------.....................................   280
CTR_H_3648bp  -----------------------.....................................   280
LMN_h2_3648bp -----------------------.....................................   280
HYG_H_3647bp  -----------------------...................T.................   222
MDT_h1_3647bp -----------------------...................T.................   222
GRP_h1_3647bp -----------------------AACCTC.T..GG.T....AA.AGAC.CT..GA...CC   289
IYK_h2_3647bp -----------------------AACCTC.T..GG.T....AA.AGAC.CT..GA...CC   289
HSS_h2_3657bp TGCTGTAGTCGGCCTTTTGCTGCA..C.A.TC..C.C..C..T.CCAG..A.TA..G...   241
SWT_h2_3657bp TGCTGTAGTCGGCCTTTTGCTGCA..C.A.TC..C.C..C..T.CCAG..A.TA..G...   241
KNN_h2_3647bp TGCTGCAGTCGGCCTTTTGCTGCA..C.A.TC..C.C..C.CT.CCAG..A.TA..G...   237
STS_h1_3647bp TGCTGCAGTCGGCCTTTTGCTGCA..C.A.TC..C.C..C.CT.CCAG..A.TA..G...   237
PMM_H_3647bp  TGCTGCAGTCGGCCTTTTGCTGCA..C.A.TC..C.C..C.CT.CCAG..A.TA..G...   237
KSH_h2_3647bp -----------------------.....................................   238
MKK_h2_3647bp -----------------------.....................................   238
PNK_h2_3647bp -----------------------.....................................   246
KNN_h1_3647bp ------------------------------------------------------------
STS_h2_3647bp ------------------------------------------------------------
MKK_h1_3647bp ------------------------------------------------------------
LMN_h1_3647bp ------------------------------------------------------------
HSS_h1_3647bp ------------------------------------------------------------
KSH_h1_3647bp ------------------------------------------------------------
```

```
KNG_h2_3647bp -----------------------.....................................   245
MRC_H_3647bp  -----------------------.....................................   246
MDT_h2_3647bp -----------------------.....................................   246
DNC_h2_3647bp -----------------------.....................................   246
GRP_h2_3647bp ------------------------------------------------------------
DNC_h1_3647bp ------------------------------------------------------------
KNG_h1_3647bp ------------------------------------------------------------
PNK_h1_3647bp ------------------------------------------------------------
SWT_h1_3647bp ------------------------------------------------------------
IYK_h1_3647bp ------------------------------------------------------------
CLM_3647bp    ------------------------------------------------------------

Consensus     GTAGTGTTTCCTCACAGCCCCAGTCTGCTTATCCGACTCCATTATCATCGACCCGCCNGC   340
OVL_h1_3647bp .................................G.......................T..   340
CTR_H_3648bp  ...............G..T......................................G..   340
LMN_h2_3648bp ...............G..T......................................G..   340
HYG_H_3647bp  ...............G.........................................G..   282
MDT_h1_3647bp ...............G.........................................G..   282
GRP_h1_3647bp A.TT-...AA.AT.A.------T.A.AA..T.AAAGTGTA....C...TATA..CTT---   339
IYK_h2_3647bp A.TT-...AA.AT.A.------T.A.AA..T.AAAGTGTA....C...TATA..CTT---   339
HSS_h2_3657bp C.TC.CCC...C.T..ATTT..TCGGCA.C...G.-------------------------   276
SWT_h2_3657bp C.TC.CCC...C.T..ATTT..TCGGCA.C...G.-------------------------   276
KNN_h2_3647bp C.TC.CCC...C.T..ATTT..TCGGCA.C...G.-------------------------   272
STS_h1_3647bp C.TC.CCC...C.T..ATTT..TCGGCA.C...G.-------------------------   272
PMM_H_3647bp  C.TC.CCC...C.T..ATTT..TCGGCA.C...G.-------------------------   272
KSH_h2_3647bp .........................................................G..   298
MKK_h2_3647bp .........................................................G..   298
PNK_h2_3647bp ...............G.........................................G..   306
KNN_h1_3647bp ------------------------------------------------------------
STS_h2_3647bp ------------------------------------------------------------
MKK_h1_3647bp ------------------------------------------------------------
LMN_h1_3647bp ------------------------------------------------------------
HSS_h1_3647bp ------------------------------------------------------------
KSH_h1_3647bp ------------------------------------------------------------
KNG_h2_3647bp .........................................................A..   305
MRC_H_3647bp  .........................................................A..   306
MDT_h2_3647bp .........................................................A..   306
DNC_h2_3647bp .........................................................A..   306
GRP_h2_3647bp ------------------------------------------------------------
DNC_h1_3647bp ------------------------------------------------------------
KNG_h1_3647bp ------------------------------------------------------------
PNK_h1_3647bp ------------------------------------------------------------
SWT_h1_3647bp ------------------------------------------------------------
IYK_h1_3647bp ------------------------------------------------------------
CLM_3647bp    ------------------------------------------------------------

Consensus     GCCGATCCACTGCCCCGGTGGCGCCGCCACCGGTAC-CGCCACCACCTTGTTGGCCACCA   399
OVL_h1_3647bp ...T.....A..........................-.......................   399
CTR_H_3648bp  ....................................-.......................   399
LMN_h2_3648bp ....................................-.......................   399
HYG_H_3647bp  ....................................-.......................   341
MDT_h1_3647bp ....................................-.......................   341
GRP_h1_3647bp ------------------------------------------AT.G.A.A..AAAA.AT.   357
IYK_h2_3647bp ------------------------------------------AT.G.A.A..AAAA.AT.   357
HSS_h2_3657bp -------...-T.T.TCT..T.AAT.G..T..T...-..A..A.CATC.C..TCT.TT..   327
SWT_h2_3657bp -------...-T.T.TCT..T.AAT.G..T..T...-..A..A.CATC.C..TCT.TT..   327
KNN_h2_3647bp -------...-T.T.TCT..T.AAT.G..T..T...-..A..A.CATC.C..TCT.TT..   323
STS_h1_3647bp -------...-T.T.TCT..T.AAT.G..T..T...-..A..A.CATC.C..TCT.TT..   323
PMM_H_3647bp  -------...-T.T.TCT..T.AAT.G..T..T...-..A..A.CATC.C..TCT.TT..   323
KSH_h2_3647bp .....................T..............-.......................   357
MKK_h2_3647bp .....................T..............-.......................   357
PNK_h2_3647bp ....................................-.......................   365
KNN_h1_3647bp -------------------------------------------.................    17
STS_h2_3647bp -------------------------------------------.................    17
MKK_h1_3647bp -------------------------------------------.................    17
LMN_h1_3647bp -------------------------------------------.................    17
HSS_h1_3647bp -------------------------------------------.................    17
KSH_h1_3647bp -------------------------------------------.................    17
```

```
KNG_h2_3647bp ....................................C...................G...   365
MRC_H_3647bp  ....................................-...................G...   365
MDT_h2_3647bp ....................................-...................G...   365
DNC_h2_3647bp ....................................-...................G...   365
GRP_h2_3647bp --------------------------------------------................    16
DNC_h1_3647bp --------------------------------------------................    16
KNG_h1_3647bp --------------------------------------------................    16
PNK_h1_3647bp --------------------------------------------................    16
SWT_h1_3647bp --------------------------------------------................    16
IYK_h1_3647bp --------------------------------------------................    16
CLM_3647bp    --------------------------------------------................    16

Consensus     ATGCGTTCATTGTGTGAGCCAAAGACGCCGTATGCGGCACCTCAACTATCCGCCTCTTCT   459
OVL_h1_3647bp ............................................................   459
CTR_H_3648bp  ...........................................G................   459
LMN_h2_3648bp ...........................................G................   459
HYG_H_3647bp  ............................................................   401
MDT_h1_3647bp ............................................................   401
GRP_h1_3647bp -ATT.........CCA.TTT.TTCGTATAT...TAATA.A--------------------   396
IYK_h2_3647bp -ATT.........CCA.TTT.TTCGTATAT...TAATA.A--------------------   396
HSS_h2_3657bp GCT...CACGACA.AC.C.ACC..T.-.G..T..TCA.TT.GTCCGG...TCT.CA..A.   386
SWT_h2_3657bp GCT...CACGACA.AC.C.ACC..T.-.G..T..TCA.TT.GTCCGG...TCT.CA..A.   386
KNN_h2_3647bp GCT...CACGACA.AC.C.ACC..T.-.G..T..TCA.TT.GTCCGG...TC..CA..A.   382
STS_h1_3647bp GCT...CACGACA.AC.C.ACC..T.-.G..T..TCA.TT.GTCCGG...TC..CA..A.   382
PMM_H_3647bp  GCT...CACGACA.AC.C.ACC..T.-.G..T..TCA.TT.GTCCGG...TC..CA..A.   382
KSH_h2_3647bp ...........................................G................   417
MKK_h2_3647bp ...........................................G................   417
PNK_h2_3647bp ............................................................   425
KNN_h1_3647bp ............................................................    77
STS_h2_3647bp ............................................................    77
MKK_h1_3647bp ............................................................    77
LMN_h1_3647bp ............................................................    77
HSS_h1_3647bp ............................................................    77
KSH_h1_3647bp ............................................................    77
KNG_h2_3647bp ............................................................   425
MRC_H_3647bp  ............................................................   425
MDT_h2_3647bp ............................................................   425
DNC_h2_3647bp ............................................................   425
GRP_h2_3647bp ............................................................    76
DNC_h1_3647bp ............................................................    76
KNG_h1_3647bp ............................................................    76
PNK_h1_3647bp ............................................................    76
SWT_h1_3647bp ............................................................    76
IYK_h1_3647bp ............................................................    76
CLM_3647bp    ............................................................    76

Consensus     CCGCCGTCAAGTCCCTCACTTTAGCCTCCCTCA--------------------GCAGCCG   499
OVL_h1_3647bp .................................--------------------..C....   499
CTR_H_3648bp  ......................C..........--------------------..C....   499
LMN_h2_3648bp ......................C..........--------------------..C....   499
HYG_H_3647bp  .....T...........................--------------------..C....   441
MDT_h1_3647bp .....T...........................--------------------..C....   441
GRP_h1_3647bp -----A.T.C.AATA.A......TTA..--------------------------------   419
IYK_h2_3647bp -----A.T.C.AATA.A......TTA..--------------------------------   419
HSS_h2_3657bp T.CAG..GTGT.T..C.TA...TTTTGTT..G.AAAACCCAAACAAATGAAGAA..A.AC   446
SWT_h2_3657bp T.CAG..GTGT.T..C.TA...TTTTGTT..G.AAAACCCAAACAAATGAAGAA..A.AC   446
KNN_h2_3647bp T.CAG..GTGT.T..C.TA...TTTTGTT..G.AAAACCCAAACAAATGAAGAA..A.AC   442
STS_h1_3647bp T.CAG..GTGT.T..C.TA...TTTTGTT..G.AAAACCCAAACAAATGAAGAA..A.AC   442
PMM_H_3647bp  T.CAG..GTGT.T..C.TA...TTTTGTT..G.AAAACCCAAACAAATGAAGAA..A.AC   442
KSH_h2_3647bp ......................C..........--------------------..C....   457
MKK_h2_3647bp ......................C..........--------------------..C....   457
PNK_h2_3647bp .................................--------------------.......   465
KNN_h1_3647bp .................................--------------------.......   117
STS_h2_3647bp .................................--------------------.......   117
MKK_h1_3647bp .................................--------------------.......   117
LMN_h1_3647bp .................................--------------------.......   117
HSS_h1_3647bp .................................--------------------.......   117
KSH_h1_3647bp .................................--------------------.......   117
```

```
KNG_h2_3647bp .................................--------------------.......   465
MRC_H_3647bp  .................................--------------------.......   465
MDT_h2_3647bp .................................--------------------.......   465
DNC_h2_3647bp .................................--------------------.......   465
GRP_h2_3647bp .................................--------------------.......   116
DNC_h1_3647bp .................................--------------------.......   116
KNG_h1_3647bp .................................--------------------.......   116
PNK_h1_3647bp .................................--------------------.......   116
SWT_h1_3647bp .................................--------------------.......   116
IYK_h1_3647bp .................................--------------------.......   116
CLM_3647bp    .................................--------------------.......   116

Consensus     CTGGTTTTCATCGGCTGCGTGTTTTCCAG-CTG-ATGATATGGGGTCTTGCTCT------   551
OVL_h1_3647bp .............................-...-....................------   551
CTR_H_3648bp  .............................-...-....................------   551
LMN_h2_3648bp .............................-...-....................------   551
HYG_H_3647bp  .............................-...-....................------   493
MDT_h1_3647bp .............................-...-....................------   493
GRP_h1_3647bp -----------------TAAT....T..T-T.A-..TT.TGTCAAAT..T....TCAACT   460
IYK_h2_3647bp -----------------TAAT....T..T-T.A-..TT.TGTCAAAT..T....TCAACT   460
HSS_h2_3657bp ...T-----..T.AT.T..AT....TG.TT.CAAG.C.A.G...TG....G-..------   494
SWT_h2_3657bp ...T-----..T.AT.T..AT....TG.TT.CAAG.C.A.G...TG....G-..------   494
KNN_h2_3647bp ...T-----..T.AT.T..AT....TG.TT.CAAG.C.A.G...TG....G-..------   490
STS_h1_3647bp ...T-----..T.AT.T..AT....TG.TT.CAAG.C.A.G...TG....G-..------   490
PMM_H_3647bp  ...T-----..T.AT.T..AT....TG.TT.CAAG.C.A.G...TG....G-..------   490
KSH_h2_3647bp ...........A.................-...-....................------   509
MKK_h2_3647bp ...........A.................-...-....................------   509
PNK_h2_3647bp .............................-...-....................------   517
KNN_h1_3647bp .............................-...-....................------   169
STS_h2_3647bp .............................-...-....................------   169
MKK_h1_3647bp .............................-...-....................------   169
LMN_h1_3647bp .............................-...-....................------   169
HSS_h1_3647bp .............................-...-....................------   169
KSH_h1_3647bp .............................-...-....................------   169
KNG_h2_3647bp .............................-...-....................------   517
MRC_H_3647bp  .............................-...-....................------   517
MDT_h2_3647bp .............................-...-....................------   517
DNC_h2_3647bp .............................-...-....................------   517
GRP_h2_3647bp .............................-...-....................------   168
DNC_h1_3647bp .............................-...-....................------   168
KNG_h1_3647bp .............................-...-....................------   168
PNK_h1_3647bp .............................-...-....................------   168
SWT_h1_3647bp .............................-...-....................------   168
IYK_h1_3647bp .............................-...-....................------   168
CLM_3647bp    .............................-...-....................------   168

Consensus     ---TTCGTTTGCTGCATTCTTGAAGATGGGATTGAAAATTGAAGTGAAGTG---------   599
OVL_h1_3647bp ---................................................---------   599
CTR_H_3648bp  ---.................C...................T..........---------   599
LMN_h2_3648bp ---.................C...................T..........---------   599
HYG_H_3647bp  ---.....................................T..........---------   541
MDT_h1_3647bp ---.....................................T..........---------   541
GRP_h1_3647bp AAA.AAA...AA.T....GC.C....ATAA.AAT.-....A..T...TTACTAATATAAT   519
IYK_h2_3647bp AAA.AAA...AA.T....GC.C....ATAA.AAT.-....A..T...TTACTAATATAAT   519
HSS_h2_3657bp ---..TAA-.TA.A..AGA.A.....C.AA.CCA..TT....T..TG.-..---------   540
SWT_h2_3657bp ---..TAA-.TA.A..AGA.A.....C.AA.CCA..TT....T..TG.-..---------   540
KNN_h2_3647bp ---..TAA-.TA.A..AGA.A.....C.AA.CCA..TT....T..TG.-..---------   536
STS_h1_3647bp ---..TAA-.TA.A..AGA.A.....C.AA.CCA..TT....T..TG.-..---------   536
PMM_H_3647bp  ---..TAA-.TA.A..AGA.A.....C.AA.CCA..TT....T..TG.-..---------   536
KSH_h2_3647bp ---................................................---------   557
MKK_h2_3647bp ---................................................---------   557
PNK_h2_3647bp ---............................................G...---------   565
KNN_h1_3647bp ---................................................---------   217
STS_h2_3647bp ---................................................---------   217
MKK_h1_3647bp ---................................................---------   217
LMN_h1_3647bp ---................................................---------   217
HSS_h1_3647bp ---................................................---------   217
KSH_h1_3647bp ---................................................---------   217
```

```
KNG_h2_3647bp ---................................................---------   565
MRC_H_3647bp  ---................................................---------   565
MDT_h2_3647bp ---................................................---------   565
DNC_h2_3647bp ---................................................---------   565
GRP_h2_3647bp ---................................................---------   216
DNC_h1_3647bp ---................................................---------   216
KNG_h1_3647bp ---................................................---------   216
PNK_h1_3647bp ---................................................---------   216
SWT_h1_3647bp ---................................................---------   216
IYK_h1_3647bp ---................................................---------   216
CLM_3647bp    ---................................................---------   216

Consensus     ---------------CACTAATGGGTTCGCATGTGAT-----------------------   621
OVL_h1_3647bp ---------------......................-----------------------   621
CTR_H_3648bp  ---------------......................-----------------------   621
LMN_h2_3648bp ---------------......................-----------------------   621
HYG_H_3647bp  ---------------......................-----------------------   563
MDT_h1_3647bp ---------------......................-----------------------   563
GRP_h1_3647bp AAAAATAACAATTCT..A...CAAT.CTT...T.A.ACTTATATAATCAACAAAATAAAA   579
IYK_h2_3647bp AAAAATAACAATTCT..A...CAAT.CTT...T.A.ACTTATATAATCAACAAAATAAAA   579
HSS_h2_3657bp ---------------A.GA.GAA.AAA.AA.AT...C-----------------------   562
SWT_h2_3657bp ---------------A.GA.GAA.AAA.AA.AT...C-----------------------   562
KNN_h2_3647bp ---------------A.GA.GAA.AAA.AA.AT...C-----------------------   558
STS_h1_3647bp ---------------A.GA.GAA.AAA.AA.AT...C-----------------------   558
PMM_H_3647bp  ---------------A.GA.GAA.AAA.AA.AT...C-----------------------   558
KSH_h2_3647bp ---------------........A.............-----------------------   579
MKK_h2_3647bp ---------------........A.............-----------------------   579
PNK_h2_3647bp ---------------......................-----------------------   587
KNN_h1_3647bp ---------------......................-----------------------   239
STS_h2_3647bp ---------------......................-----------------------   239
MKK_h1_3647bp ---------------......................-----------------------   239
LMN_h1_3647bp ---------------......................-----------------------   239
HSS_h1_3647bp ---------------......................-----------------------   239
KSH_h1_3647bp ---------------......................-----------------------   239
KNG_h2_3647bp ---------------......................-----------------------   587
MRC_H_3647bp  ---------------......................-----------------------   587
MDT_h2_3647bp ---------------......................-----------------------   587
DNC_h2_3647bp ---------------......................-----------------------   587
GRP_h2_3647bp ---------------................A.....-----------------------   238
DNC_h1_3647bp ---------------................A.....-----------------------   238
KNG_h1_3647bp ---------------................A.....-----------------------   238
PNK_h1_3647bp ---------------................A.....-----------------------   238
SWT_h1_3647bp ---------------................A.....-----------------------   238
IYK_h1_3647bp ---------------................A.....-----------------------   238
CLM_3647bp    ---------------................A.....-----------------------   238

Consensus     ---GTGTGTTATTATATGAT---------------------------GCGTATGATTTTG   651
OVL_h1_3647bp ---.................---------------------------.............   651
CTR_H_3648bp  ---......C..........---------------------------...C.........   651
LMN_h2_3648bp ---......C..........---------------------------...C.........   651
HYG_H_3647bp  ---.................---------------------------.............   593
MDT_h1_3647bp ---.................---------------------------.............   593
GRP_h1_3647bp AAAA.TG....CA.C.AT.A---------------------------TTAC.AA..C..A   612
IYK_h2_3647bp AAAA.TG....CA.C.AT.A---------------------------TTAC.AA..C..A   612
HSS_h2_3657bp ---AG.A.GA.GA.G.A...AATTGTTATGATGATGTTGTTGATGAA.AAG....AA...   619
SWT_h2_3657bp ---AG.A.GA.GA.G.A...AATTGTTATGATGATGTTGTTGATGAA.AAG....AA...   619
KNN_h2_3647bp ---AG.A.GA.GA.G.A...AATTGTTATGATGATGTTGTTGATGAA.AAG....AA...   615
STS_h1_3647bp ---AG.A.GA.GA.G.A...AATTGTTATGATGATGTTGTTGATGAA.AAG....AA...   615
PMM_H_3647bp  ---AG.A.GA.GA.G.A...AATTGTTATGATGATGTTGTTGATGAA.AAG....AA...   615
KSH_h2_3647bp ---.................---------------------------.............   609
MKK_h2_3647bp ---.................---------------------------.............   609
PNK_h2_3647bp ---................C---------------------------.............   617
KNN_h1_3647bp ---................C---------------------------.............   269
STS_h2_3647bp ---................C---------------------------.............   269
MKK_h1_3647bp ---................C---------------------------.............   269
LMN_h1_3647bp ---................C---------------------------.............   269
HSS_h1_3647bp ---................C---------------------------.............   269
KSH_h1_3647bp ---................C---------------------------.............   269
```

```
KNG_h2_3647bp ---.................---------------------------.............   617
MRC_H_3647bp  ---.................---------------------------.............   617
MDT_h2_3647bp ---.................---------------------------.............   617
DNC_h2_3647bp ---.................---------------------------.............   617
GRP_h2_3647bp ---................C---------------------------.............   268
DNC_h1_3647bp ---................C---------------------------.............   268
KNG_h1_3647bp ---................C---------------------------.............   268
PNK_h1_3647bp ---................C---------------------------.............   268
SWT_h1_3647bp ---................C---------------------------.............   268
IYK_h1_3647bp ---................C---------------------------.............   268
CLM_3647bp    ---................C---------------------------.............   268

Consensus     AGTACGTTTCGTGCATGCGCCGCACGTGGCGACGAAGGATTACGCATCATTGCATG----   707
OVL_h1_3647bp ............................................T...........----   707
CTR_H_3648bp  ........A............A......................T...........----   707
LMN_h2_3648bp ........A............A......................T...........----   707
HYG_H_3647bp  ........A...................................T...........----   649
MDT_h1_3647bp ........A...................................T...........----   649
GRP_h1_3647bp .T.GTAC.AAA.AA.ATG.AAAA.TTG..TA.T....TTA..TT...A...TT..T----   668
IYK_h2_3647bp .T.GTAC.AAA.AA.ATG.AAAA.TTG..TA.T....TTA..TT...A...TT..T----   668
HSS_h2_3657bp .CG.T.GAGA.AAGTAA.AAA.A.AT..CA..A..GA..AA.A.A.AA.AGTG.A.AAGA   679
SWT_h2_3657bp .CG.T.GAGA.AAGTAA.AAA.A.AT..CA..A..GA..AA.A.A.AA.AGTG.A.AAGA   679
KNN_h2_3647bp .CG.T.GAGA.AAGTAA.AAA.A.AT..CA..A..GA..GA.A.A.AA.AGTG.A.AAGA   675
STS_h1_3647bp .CG.T.GAGA.AAGTAA.AAA.A.AT..CA..A..GA..GA.A.A.AA.AGTG.A.AAGA   675
PMM_H_3647bp  .CG.T.GAGA.AAGTAA.AAA.A.AT..CA..A..GA..GA.A.A.AA.AGTG.A.AAGA   675
KSH_h2_3647bp ...C.........................................C..........----   665
MKK_h2_3647bp ...C.........................................C..........----   665
PNK_h2_3647bp ........................................................----   673
KNN_h1_3647bp ........................................................----   325
STS_h2_3647bp ........................................................----   325
MKK_h1_3647bp ........................................................----   325
LMN_h1_3647bp ........................................................----   325
HSS_h1_3647bp ........................................................----   325
KSH_h1_3647bp ........................................................----   325
KNG_h2_3647bp ........................................................----   673
MRC_H_3647bp  ........................................................----   673
MDT_h2_3647bp ........................................................----   673
DNC_h2_3647bp ........................................................----   673
GRP_h2_3647bp ........................................................----   324
DNC_h1_3647bp ........................................................----   324
KNG_h1_3647bp ........................................................----   324
PNK_h1_3647bp ........................................................----   324
SWT_h1_3647bp ........................................................----   324
IYK_h1_3647bp ........................................................----   324
CLM_3647bp    ........................................................----   324

Consensus     -------GCTAAG----CAGTGTCTGCCACGTTGAATAGTTG------------------   738
OVL_h1_3647bp -------......----.........................------------------   738
CTR_H_3648bp  -------......----.........................------------------   738
LMN_h2_3648bp -------......----.........................------------------   738
HYG_H_3647bp  -------......----.........................------------------   680
MDT_h1_3647bp -------......----.........................------------------   680
GRP_h1_3647bp -------AA...TATAGA.A.AAA.TGG..AA....C.A..A------------------   703
IYK_h2_3647bp -------AA...TATAGA.A.AAA.TGG..AA....C.A..A------------------   703
HSS_h2_3657bp TAAAAGAAGA..A----A.AC.GAA.AG.GA.AA....A.AT------------------   717
SWT_h2_3657bp TAAAAGAAGA..A----A.AC.GAA.AG.GA.AA....A.AT------------------   717
KNN_h2_3647bp TAAAAGAAGA..A----A.ACAGAA.AG.GA.AA....A.AT------------------   713
STS_h1_3647bp TAAAAGAAGA..A----A.ACAGAA.AG.GA.AA....A.AT------------------   713
PMM_H_3647bp  TAAAAGAAGA..A----A.ACAGAA.AG.GA.AA....A.AT------------------   713
KSH_h2_3647bp -------......----.........................------------------   696
MKK_h2_3647bp -------......----.........................------------------   696
PNK_h2_3647bp -------......----.........................------------------   704
KNN_h1_3647bp -------......----.........................TAGTTCCTGTTTGGTATG   374
STS_h2_3647bp -------......----.........................TAGTTCCTGTTTGGTATG   374
MKK_h1_3647bp -------......----.........................TAGTTCCTGTTTGGTATG   374
LMN_h1_3647bp -------......----.........................TAGTTCCTGTTTGGTATG   374
HSS_h1_3647bp -------......----.........................TAGTTCCTGTTTGGTATG   374
KSH_h1_3647bp -------......----.........................TAGTTCCTGTTTGGTATG   374
```

```
KNG_h2_3647bp -------......----.........................------------------   704
MRC_H_3647bp  -------......----.........................------------------   704
MDT_h2_3647bp -------......----.........................------------------   704
DNC_h2_3647bp -------......----.........................------------------   704
GRP_h2_3647bp -------......----.........................TAGTTCCTGTTTGGTATG   373
DNC_h1_3647bp -------......----.........................TAGTTCCTGTTTGGTATG   373
KNG_h1_3647bp -------......----.........................TAGTTCCTGTTTGGTATG   373
PNK_h1_3647bp -------......----.........................TAGTTCCTGTTTGGTATG   373
SWT_h1_3647bp -------......----.........................TAGTTCCTGTTTGGTATG   373
IYK_h1_3647bp -------......----.........................TAGTTCCTGTTTGGTATG   373
CLM_3647bp    -------......----.........................TAGTTCCTGTTTGGTATG   373

Consensus     ------------------------------------------------------------   738
OVL_h1_3647bp ------------------------------------------------------------   738
CTR_H_3648bp  ------------------------------------------------------------   738
LMN_h2_3648bp ------------------------------------------------------------   738
HYG_H_3647bp  ------------------------------------------------------------   680
MDT_h1_3647bp ------------------------------------------------------------   680
GRP_h1_3647bp ------------------------------------------------------------   703
IYK_h2_3647bp ------------------------------------------------------------   703
HSS_h2_3657bp ------------------------------------------------------------   717
SWT_h2_3657bp ------------------------------------------------------------   717
KNN_h2_3647bp ------------------------------------------------------------   713
STS_h1_3647bp ------------------------------------------------------------   713
PMM_H_3647bp  ------------------------------------------------------------   713
KSH_h2_3647bp ------------------------------------------------------------   696
MKK_h2_3647bp ------------------------------------------------------------   696
PNK_h2_3647bp ------------------------------------------------------------   704
KNN_h1_3647bp ACTTATTTAAGAGGCATAAGTACTTATTTAATCGTATAAGCACTTTTTAAAAATTTTTAT   434
STS_h2_3647bp ACTTATTTAAGAGGCATAAGTACTTATTTAATCGTATAAGCACTTTTTAAAAATTTTTAT   434
MKK_h1_3647bp ACTTATTTAAGAGGCATAAGTACTTATTTAATCGTATAAGCACTTTTTAAAAATTTTTAT   434
LMN_h1_3647bp ACTTATTTAAGAGGCATAAGTACTTATTTAATCGTATAAGCACTTTTTAAAAATTTTTAT   434
HSS_h1_3647bp ACTTATTTAAGAGGCATAAGTACTTATTTAATCGTATAAGCACTTTTTAAAAATTTTTAT   434
KSH_h1_3647bp ACTTATTTAAGAGGCATAAGTACTTATTTAATCGTATAAGCACTTTTTAAAAATTTTTAT   434
KNG_h2_3647bp ------------------------------------------------------------   704
MRC_H_3647bp  ------------------------------------------------------------   704
MDT_h2_3647bp ------------------------------------------------------------   704
DNC_h2_3647bp ------------------------------------------------------------   704
GRP_h2_3647bp GCTTATTTAAGAGGCATAAGTACTTATTTAATCGTATAAACACTTTTTAAAATTTTTTAT   433
DNC_h1_3647bp GCTTATTTAAGAGGCATAAGTACTTATTTAATCGTATAAACACTTTTTAAAATTTTTTAT   433
KNG_h1_3647bp GCTTATTTAAGAGGCATAAGTACTTATTTAATCGTATAAACACTTTTTAAAATTTTTTAT   433
PNK_h1_3647bp GCTTATTTAAGAGGCATAAGTACTTATTTAATCGTATAAACACTTTTTAAAATTTTTTAT   433
SWT_h1_3647bp GCTTATTTAAGAGGCATAAGTACTTATTTAATCGTATAAACACTTTTTAAAATTTTTTAT   433
IYK_h1_3647bp GCTTATTTAAGAGGCATAAGTACTTATTTAATCGTATAAACACTTTTTAAAATTTTTTAT   433
CLM_3647bp    GCTTATTTAAGAGGCATAAGTACTTATTTAATCGTATAAACACTTTTTAAAATTTTTTAT   433

Consensus     ------------------------------------------------------------   738
OVL_h1_3647bp ------------------------------------------------------------   738
CTR_H_3648bp  ------------------------------------------------------------   738
LMN_h2_3648bp ------------------------------------------------------------   738
HYG_H_3647bp  ------------------------------------------------------------   680
MDT_h1_3647bp ------------------------------------------------------------   680
GRP_h1_3647bp ------------------------------------------------------------   703
IYK_h2_3647bp ------------------------------------------------------------   703
HSS_h2_3657bp ------------------------------------------------------------   717
SWT_h2_3657bp ------------------------------------------------------------   717
KNN_h2_3647bp ------------------------------------------------------------   713
STS_h1_3647bp ------------------------------------------------------------   713
PMM_H_3647bp  ------------------------------------------------------------   713
KSH_h2_3647bp ------------------------------------------------------------   696
MKK_h2_3647bp ------------------------------------------------------------   696
PNK_h2_3647bp ------------------------------------------------------------   704
KNN_h1_3647bp GGTGTTTGGTTATTTTTTTAGTAGAGTTTTTTAAGCTTAAATAAGCTAATTTACATCTAC   494
STS_h2_3647bp GGTGTTTGGTTATTTTTTTAGTAGAGTTTTTTAAGCTTAAATAAGCTAATTTACATCTAC   494
MKK_h1_3647bp GGTGTTTGGTTATTTTTTTAGTAGAGTTTTTTAAGCTTAAATAAGCTAATTTACATCTAC   494
LMN_h1_3647bp GGTGTTTGGTTATTTTTTTAGTAGAGTTTTTTAAGCTTAAATAAGCTAATTTACATCTAC   494
HSS_h1_3647bp GGTGTTTGGTTATTTTTTTAGTAGAGTTTTTTAAGCTTAAATAAGCTAATTTACATCTAC   494
KSH_h1_3647bp GGTGTTTGGTTATTTTTTTAGTAGAGTTTTTTAAGCTTAAATAAGCTAATTTACATCTAC   494
```

```
KNG_h2_3647bp ------------------------------------------------------------   704
MRC_H_3647bp  ------------------------------------------------------------   704
MDT_h2_3647bp ------------------------------------------------------------   704
DNC_h2_3647bp ------------------------------------------------------------   704
GRP_h2_3647bp GGTGTTTGGTTATTTTTTTAGTAGAGTTTTTTAAGCTTAAATAAGCTAATTTACATCTAC   493
DNC_h1_3647bp GGTGTTTGGTTATTTTTTTAGTAGAGTTTTTTAAGCTTAAATAAGCTAATTTACATCTAC   493
KNG_h1_3647bp GGTGTTTGGTTATTTTTTTAGTAGAGTTTTTTAAGCTTAAATAAGCTAATTTACATCTAC   493
PNK_h1_3647bp GGTGTTTGGTTATTTTTTTAGTAGAGTTTTTTAAGCTTAAATAAGCTAATTTACATCTAC   493
SWT_h1_3647bp GGTGTTTGGTTATTTTTTTAGTAGAGTTTTTTAAGCTTAAATAAGCTAATTTACATCTAC   493
IYK_h1_3647bp GGTGTTTGGTTATTTTTTTAGTAGAGTTTTTTAAGCTTAAATAAGCTAATTTACATCTAC   493
CLM_3647bp    GGTGTTTGGTTATTTTTTTAGTAGAGTTTTTTAAGCTTAAATAAGCTAATTTACATCTAC   493

Consensus     ------------------------------------------------------------   738
OVL_h1_3647bp ------------------------------------------------------------   738
CTR_H_3648bp  ------------------------------------------------------------   738
LMN_h2_3648bp ------------------------------------------------------------   738
HYG_H_3647bp  ------------------------------------------------------------   680
MDT_h1_3647bp ------------------------------------------------------------   680
GRP_h1_3647bp ------------------------------------------------------------   703
IYK_h2_3647bp ------------------------------------------------------------   703
HSS_h2_3657bp ------------------------------------------------------------   717
SWT_h2_3657bp ------------------------------------------------------------   717
KNN_h2_3647bp ------------------------------------------------------------   713
STS_h1_3647bp ------------------------------------------------------------   713
PMM_H_3647bp  ------------------------------------------------------------   713
KSH_h2_3647bp ------------------------------------------------------------   696
MKK_h2_3647bp ------------------------------------------------------------   696
PNK_h2_3647bp ------------------------------------------------------------   704
KNN_h1_3647bp TAGCAAAAACCCAAAATTTGAGCTTTTAGGAGTAGAGGTTATAGAGTTTTTCTAAAAATA   554
STS_h2_3647bp TAGCAAAAACCCAAAATTTGAGCTTTTAGGAGTAGAGGTTATAGAGTTTTTCTAAAAATA   554
MKK_h1_3647bp TAGCAAAAACCCAAAATTTGAGCTTTTAGGAGTAGAGGTTATAGAGTTTTTCTAAAAATA   554
LMN_h1_3647bp TAGCAAAAACCCAAAATTTGAGCTTTTAGGAGTAGAGGTTATAGAGTTTTTCTAAAAATA   554
HSS_h1_3647bp TAGCAAAAACCCAAAATTTGAGCTTTTAGGAGTAGAGGTTATAGAGTTTTTCTAAAAATA   554
KSH_h1_3647bp TAGCAAAAACCCAAAATTTGAGCTTTTAGGAGTAGAGGTTATAGAGTTTTTCTAAAAATA   554
KNG_h2_3647bp ------------------------------------------------------------   704
MRC_H_3647bp  ------------------------------------------------------------   704
MDT_h2_3647bp ------------------------------------------------------------   704
DNC_h2_3647bp ------------------------------------------------------------   704
GRP_h2_3647bp TAGCAAAAGCCCAAAATTTGAGCTTTTAGGAGTAGAGGTTATAGAGCTTTTCTAAAAATA   553
DNC_h1_3647bp TAGCAAAAGCCCAAAATTTGAGCTTTTAGGAGTAGAGGTTATAGAGCTTTTCTAAAAATA   553
KNG_h1_3647bp TAGCAAAAGCCCAAAATTTGAGCTTTTAGGAGTAGAGGTTATAGAGCTTTTCTAAAAATA   553
PNK_h1_3647bp TAGCAAAAGCCCAAAATTTGAGCTTTTAGGAGTAGAGGTTATAGAGCTTTTCTAAAAATA   553
SWT_h1_3647bp TAGCAAAAGCCCAAAATTTGAGCTTTTAGGAGTAGAGGTTATAGAGCTTTTCTAAAAATA   553
IYK_h1_3647bp TAGCAAAAGCCCAAAATTTGAGCTTTTAGGAGTAGAGGTTATAGAGCTTTTCTAAAAATA   553
CLM_3647bp    TAGCAAAAGCCCAAAATTTGAGCTTTTAGGAGTAGAGGTTATAGAGCTTTTCTAAAAATA   553

Consensus     ------------------------------------------------------------   738
OVL_h1_3647bp ------------------------------------------------------------   738
CTR_H_3648bp  ------------------------------------------------------------   738
LMN_h2_3648bp ------------------------------------------------------------   738
HYG_H_3647bp  ------------------------------------------------------------   680
MDT_h1_3647bp ------------------------------------------------------------   680
GRP_h1_3647bp ------------------------------------------------------------   703
IYK_h2_3647bp ------------------------------------------------------------   703
HSS_h2_3657bp ------------------------------------------------------------   717
SWT_h2_3657bp ------------------------------------------------------------   717
KNN_h2_3647bp ------------------------------------------------------------   713
STS_h1_3647bp ------------------------------------------------------------   713
PMM_H_3647bp  ------------------------------------------------------------   713
KSH_h2_3647bp ------------------------------------------------------------   696
MKK_h2_3647bp ------------------------------------------------------------   696
PNK_h2_3647bp ------------------------------------------------------------   704
KNN_h1_3647bp TATAATATAAAAAATATCACATAGTTTAATGGTTCAAAAGTACTTTTTTTATTGCAACCA   614
STS_h2_3647bp TATAATATAAAAAATATCACATAGTTTAATGGTTCAAAAGTACTTTTTTTATTGCAACCA   614
MKK_h1_3647bp TATAATATAAAAAATATCACATAGTTTAATGGTTCAAAAGTACTTTTTTTATTGCAACCA   614
LMN_h1_3647bp TATAATATAAAAAATATCACATAGTTTAATGGTTCAAAAGTACTTTTTTTATTGCAACCA   614
HSS_h1_3647bp TATAATATAAAAAATATCACATAGTTTAATGGTTCAAAAGTACTTTTTTTATTGCAACCA   614
KSH_h1_3647bp TATAATATAAAAAATATCACATAGTTTAATGGTTCAAAAGTACTTTTTTTATTGCAACCA   614
```

```
KNG_h2_3647bp ------------------------------------------------------------   704
MRC_H_3647bp  ------------------------------------------------------------   704
MDT_h2_3647bp ------------------------------------------------------------   704
DNC_h2_3647bp ------------------------------------------------------------   704
GRP_h2_3647bp TATAATATAAAAAATATCACATAGTTTAATGGTTCAAAAGTACTTTTTTTATTGCAACCA   613
DNC_h1_3647bp TATAATATAAAAAATATCACATAGTTTAATGGTTCAAAAGTACTTTTTTTATTGCAACCA   613
KNG_h1_3647bp TATAATATAAAAAATATCACATAGTTTAATGGTTCAAAAGTACTTTTTTTATTGCAACCA   613
PNK_h1_3647bp TATAATATAAAAAATATCACATAGTTTAATGGTTCAAAAGTACTTTTTTTATTGCAACCA   613
SWT_h1_3647bp TATAATATAAAAAATATCACATAGTTTAATGGTTCAAAAGTACTTTTTTTATTGCAACCA   613
IYK_h1_3647bp TATAATATAAAAAATATCACATAGTTTAATGGTTCAAAAGTACTTTTTTTATTGCAACCA   613
CLM_3647bp    TATAATATAAAAAATATCACATAGTTTAATGGTTCAAAAGTACTTTTTTTATTGCAACCA   613

Consensus     ------------------------------------------------------------   738
OVL_h1_3647bp ------------------------------------------------------------   738
CTR_H_3648bp  ------------------------------------------------------------   738
LMN_h2_3648bp ------------------------------------------------------------   738
HYG_H_3647bp  ------------------------------------------------------------   680
MDT_h1_3647bp ------------------------------------------------------------   680
GRP_h1_3647bp ------------------------------------------------------------   703
IYK_h2_3647bp ------------------------------------------------------------   703
HSS_h2_3657bp ------------------------------------------------------------   717
SWT_h2_3657bp ------------------------------------------------------------   717
KNN_h2_3647bp ------------------------------------------------------------   713
STS_h1_3647bp ------------------------------------------------------------   713
PMM_H_3647bp  ------------------------------------------------------------   713
KSH_h2_3647bp ------------------------------------------------------------   696
MKK_h2_3647bp ------------------------------------------------------------   696
PNK_h2_3647bp ------------------------------------------------------------   704
KNN_h1_3647bp AACACCCAATGACTTTTTGTCCAAAAGCTCTATTGGTATAAGCTCTACTACTATAAGCTC   674
STS_h2_3647bp AACACCCAATGACTTTTTGTCCAAAAGCTCTATTGGTATAAGCTCTACTACTATAAGCTC   674
MKK_h1_3647bp AACACCCAATGACTTTTTGTCCAAAAGCTCTATTGGTATAAGCTCTACTACTATAAGCTC   674
LMN_h1_3647bp AACACCCAATGACTTTTTGTCCAAAAGCTCTATTGGTATAAGCTCTACTACTATAAGCTC   674
HSS_h1_3647bp AACACCCAATGACTTTTTGTCCAAAAGCTCTATTGGTATAAGCTCTACTACTATAAGCTC   674
KSH_h1_3647bp AACACCCAATGACTTTTTGTCCAAAAGCTCTATTGGTATAAGCTCTACTACTATAAGCTC   674
KNG_h2_3647bp ------------------------------------------------------------   704
MRC_H_3647bp  ------------------------------------------------------------   704
MDT_h2_3647bp ------------------------------------------------------------   704
DNC_h2_3647bp ------------------------------------------------------------   704
GRP_h2_3647bp AACACCCAATGACTTTTTATCCAAAAACTCTATTGGTATAAGCTCTACTACTATAAGCTC   673
DNC_h1_3647bp AACACCCAATGACTTTTTATCCAAAAACTCTATTGGTATAAGCTCTACTACTATAAGCTC   673
KNG_h1_3647bp AACACCCAATGACTTTTTATCCAAAAACTCTATTGGTATAAGCTCTACTACTATAAGCTC   673
PNK_h1_3647bp AACACCCAATGACTTTTTATCCAAAAACTCTATTGGTATAAGCTCTACTACTATAAGCTC   673
SWT_h1_3647bp AACACCCAATGACTTTTTATCCAAAAACTCTATTGGTATAAGCTCTACTACTATAAGCTC   673
IYK_h1_3647bp AACACCCAATGACTTTTTATCCAAAAACTCTATTGGTATAAGCTCTACTACTATAAGCTC   673
CLM_3647bp    AACACCCAATGACTTTTTATCCAAAAACTCTATTGGTATAAGCTCTACTACTATAAGCTC   673

Consensus     -----------------------------TAATGACAAA--ACGATAAGCTTGTTATCAA   767
OVL_h1_3647bp -----------------------------..........--.................G.   767
CTR_H_3648bp  -----------------------------..........--...................   767
LMN_h2_3648bp -----------------------------..........--...................   767
HYG_H_3647bp  -----------------------------..........--...................   709
MDT_h1_3647bp -----------------------------..........--...................   709
GRP_h1_3647bp -----------------------------.TT.--------TTA...T...ATAATGGT.   726
IYK_h2_3647bp -----------------------------.TT.--------TTA...T...ATAATGGT.   726
HSS_h2_3657bp -----------------------------A..AA.....AG.G..A.T....AAAG.A..   748
SWT_h2_3657bp -----------------------------A..AA.....AG.G..A.T....AAAG.A..   748
KNN_h2_3647bp -----------------------------A..AA.....AG.G..A.T....AAAG.A..   744
STS_h1_3647bp -----------------------------A..AA.....AG.G..A.T....AAAG.A..   744
PMM_H_3647bp  -----------------------------A..AA.....AG.G..A.T....AAAG.A..   744
KSH_h2_3647bp -----------------------------..........--...................   725
MKK_h2_3647bp -----------------------------..........--...................   725
PNK_h2_3647bp -----------------------------..........--...................   733
KNN_h1_3647bp TATTTAATAAGCTGTACCAAACGGAGCCG..........--...................   732
STS_h2_3647bp TATTTAATAAGCTGTACCAAACGGAGCCG..........--...................   732
MKK_h1_3647bp TATTTAATAAGCTGTACCAAACGGAGCCG..........--...................   732
LMN_h1_3647bp TATTTAATAAGCTGTACCAAACGGAGCCG..........--...................   732
HSS_h1_3647bp TATTTAATAAGCTGTACCAAACGGAGCCG..........--...................   732
KSH_h1_3647bp TATTTAATAAGCTGTACCAAACGGAGCCG..........--...................   732
```

```
KNG_h2_3647bp -----------------------------..........--..............T....   733
MRC_H_3647bp  -----------------------------..........--..............T....   733
MDT_h2_3647bp -----------------------------..........--..............T....   733
DNC_h2_3647bp -----------------------------..........--..............T....   733
GRP_h2_3647bp TATTTAATAAGCTGTACCAAACAGAGCCG..........--...................   731
DNC_h1_3647bp TATTTAATAAGCTGTACCAAACAGAGCCG..........--...................   731
KNG_h1_3647bp TATTTAATAAGCTGTACCAAACAGAGCCG..........--...................   731
PNK_h1_3647bp TATTTAATAAGCTGTACCAAACAGAGCCG..........--...................   731
SWT_h1_3647bp TATTTAATAAGCTGTACCAAACAGAGCCG..........--...................   731
IYK_h1_3647bp TATTTAATAAGCTGTACCAAACAGAGCCG..........--...................   731
CLM_3647bp    TATTTAATAAGCTGTACCAAACAGAGCCG..........--...................   731

Consensus     GCACGTACATGGC-TCGTGGAGTGGCTCATT------------TATCTTCTC-----TTT   809
OVL_h1_3647bp ..G..........-.......C.........------------.....A...-----...   809
CTR_H_3648bp  .............-.................------------.....C...-----...   809
LMN_h2_3648bp .............-.................------------.....C...-----...   809
HYG_H_3647bp  .............-.................------------.........-----...   751
MDT_h1_3647bp .............-.................------------.........-----...   751
GRP_h1_3647bp TA.T.GTA..T..-A.T.TA.AATTA.A...------------.C.G..AA.AAATAGGG   773
IYK_h2_3647bp TA.T.GTA..T..-A.T.TA.AATTA.A...------------.C.G..AA.AAATAGGG   773
HSS_h2_3657bp .GGG.GGTGGA..CAA.A.A.CA--.G.GCA------------A.A.G..AG-----...   789
SWT_h2_3657bp .GGG.GGTGGA..CAA.A.A.CA--.G.GCA------------A.A.G..AG-----...   789
KNN_h2_3647bp .TGG.GGTGG...CAA.A.A.CA--.G.GCAAAACGTCAGCACA.A..G..A-----...   797
STS_h1_3647bp .TGG.GGTGG...CAA.A.A.CA--.G.GCAAAACGTCAGCACA.A..G..A-----...   797
PMM_H_3647bp  .TGG.GGTGG...CAA.A.A.CA--.G.GCAAAACGTCAGCACA.A..G..A-----...   797
KSH_h2_3647bp .............-.................------------.........-----...   767
MKK_h2_3647bp .............-.................------------.........-----...   767
PNK_h2_3647bp .............-.................------------.........-----...   775
KNN_h1_3647bp .............-.................------------.........-----...   774
STS_h2_3647bp .............-.................------------.........-----...   774
MKK_h1_3647bp .............-.................------------.........-----...   774
LMN_h1_3647bp .............-.................------------.........-----...   774
HSS_h1_3647bp .............-.................------------.........-----...   774
KSH_h1_3647bp .............-.................------------.........-----...   774
KNG_h2_3647bp .............-.................------------.........-----...   775
MRC_H_3647bp  .............-.................------------.........-----...   775
MDT_h2_3647bp .............-.................------------.........-----...   775
DNC_h2_3647bp .............-.................------------.........-----...   775
GRP_h2_3647bp .............-.................------------.........-----...   773
DNC_h1_3647bp .............-.................------------.........-----...   773
KNG_h1_3647bp .............-.................------------.........-----...   773
PNK_h1_3647bp .............-.................------------.........-----...   773
SWT_h1_3647bp .............-.................------------.........-----...   773
IYK_h1_3647bp .............-.................------------.........-----...   773
CLM_3647bp    .............-.................------------.........-----...   773

Consensus     CTCTAATTTTCTACTATTTTTGCTCATTCTTTT-TCTTTTTTCTT------TTTCTT-TT   861
OVL_h1_3647bp .A...............................-.......-----------.....-..   856
CTR_H_3648bp  ..............C..................-...........-----T......-..   862
LMN_h2_3648bp ..............C..................-...........-----T......-..   862
HYG_H_3647bp  .................................T...........-----T......-..   805
MDT_h1_3647bp .................................T...........-----T......-..   805
GRP_h1_3647bp G...T.G.G...TT.T.GA------.AC...AG-GGGG....G.G------.CC...T..   820
IYK_h2_3647bp G...T.G.G...TT.T.GA------.AC...AG-GGGG....G.G------.CC...T..   820
HSS_h2_3657bp ....C.GGG.TCT..CAG...T.C.GACG..AA-G.A....C.A.------G..------   836
SWT_h2_3657bp ....C.GGG.TCT..CAG...T.C.GACG..AA-G.A....C.A.------G..------   836
KNN_h2_3647bp ....C.GAG.TCT..CAGCC.T.C.GACG..AA-G.A....C------------------   838
STS_h1_3647bp ....C.GAG.TCT..CAGCC.T.C.GACG..AA-G.A....C------------------   838
PMM_H_3647bp  ....C.GAG.TCT..CAGCC.T.C.GACG..AA-G.A....C------------------   838
KSH_h2_3647bp .................................-...........TTTCTT......-..   825
MKK_h2_3647bp .................................-...........TTTCTT......-..   825
PNK_h2_3647bp .................................-...........------......-..   827
KNN_h1_3647bp .................................-...........------......-..   826
STS_h2_3647bp .................................-...........------......-..   826
MKK_h1_3647bp .................................-...........------......-..   826
LMN_h1_3647bp .................................-...........------......-..   826
HSS_h1_3647bp .................................-...........------......-..   826
KSH_h1_3647bp .................................-...........------......-..   826
```

```
KNG_h2_3647bp  .................................-...........------......-..   827
MRC_H_3647bp   .................................-...........------......-..   827
MDT_h2_3647bp  .................................-...........------......-..   827
DNC_h2_3647bp  .................................-...........------......-..   827
GRP_h2_3647bp  .................................-...........------......-..   825
DNC_h1_3647bp  .................................-...........------......-..   825
KNG_h1_3647bp  .................................-...........------......-..   825
PNK_h1_3647bp  .................................-...........------......-..   825
SWT_h1_3647bp  .................................-...........------......-..   825
IYK_h1_3647bp  .................................-...........------......-..   825
CLM_3647bp     .................................-...........------......-..   825

Consensus      TTGGGGTAAGGGCTAATGATAAAATGTTCAATA-AGAGAAATTTATCGTCCCTT-ACAAA   919
OVL_h1_3647bp  ...A.......AG....................-.................T..-.....   914
CTR_H_3648bp   .................................-....................-.....   920
LMN_h2_3648bp  .................................-....................-.....   920
HYG_H_3647bp   ...........................C.....-....................-.....   863
MDT_h1_3647bp  ...........................C.....-....................-.....   863
GRP_h1_3647bp  CAAAC..T....G..TCT-----G...CTCT..TTC.A.C...AGGG...T..GTGTCCT   875
IYK_h2_3647bp  CAAAC..T....G..TCT-----G...CTCT..TTC.A.C...AGGG...T..GTGTCCT   875
HSS_h2_3657bp  ----------------------C..........-....................-.....   872
SWT_h2_3657bp  ----------------------C..........-....................-.....   872
KNN_h2_3647bp  ----------------------C.........G-....................-.....   874
STS_h1_3647bp  ----------------------C.........G-....................-.....   874
PMM_H_3647bp   ----------------------C.........G-....................-.....   874
KSH_h2_3647bp  .................................-....................-.....   883
MKK_h2_3647bp  .................................-....................-.....   883
PNK_h2_3647bp  .................................-....................-.....   885
KNN_h1_3647bp  .................................-....................-.....   884
STS_h2_3647bp  .................................-....................-.....   884
MKK_h1_3647bp  .................................-....................-.....   884
LMN_h1_3647bp  .................................-....................-.....   884
HSS_h1_3647bp  .................................-....................-.....   884
KSH_h1_3647bp  .................................-....................-.....   884
KNG_h2_3647bp  .................................-....................-.....   885
MRC_H_3647bp   .................................-....................-.....   885
MDT_h2_3647bp  .................................-....................-.....   885
DNC_h2_3647bp  .................................-....................-.....   885
GRP_h2_3647bp  .................................-....................-.....   883
DNC_h1_3647bp  .................................-....................-.....   883
KNG_h1_3647bp  .................................-....................-.....   883
PNK_h1_3647bp  .................................-....................-.....   883
SWT_h1_3647bp  .................................-....................-.....   883
IYK_h1_3647bp  .................................-....................-.....   883
CLM_3647bp     .................................-....................-.....   883

Consensus      AGTAAAATAAAATATTGCAGCAACTTTTTAAATCACTGAAAAATTGAA-AATTAACTTTA   978
OVL_h1_3647bp  ..........................--..T...........T.....A...........   972
CTR_H_3648bp   ...............C..............T.................-...........   979
LMN_h2_3648bp  ...............C..............T.................-...........   979
HYG_H_3647bp   ..............................T...........T.....-...........   922
MDT_h1_3647bp  ..............................T...........T.....-...........   922
GRP_h1_3647bp  TT.CCC........................T....T............-.....C.....   934
IYK_h2_3647bp  TT.CCC........................T....T............-.....C.....   934
HSS_h2_3657bp  ..............................T...........T.....-.....C.....   931
SWT_h2_3657bp  ..............................T...........T.....-.....C.....   931
KNN_h2_3647bp  ..............................T.................-.....C.....   933
STS_h1_3647bp  ..............................T.................-.....C.....   933
PMM_H_3647bp   ..............................T.................-.....C.....   933
KSH_h2_3647bp  ................................................-.........A.   942
MKK_h2_3647bp  ................................................-.........A.   942
PNK_h2_3647bp  ................................................-...........   944
KNN_h1_3647bp  ................................................-...........   943
STS_h2_3647bp  ................................................-...........   943
MKK_h1_3647bp  ................................................-...........   943
LMN_h1_3647bp  ................................................-...........   943
HSS_h1_3647bp  ................................................-...........   943
KSH_h1_3647bp  ................................................-...........   943
```

```
KNG_h2_3647bp ................................................-.........A.   944
MRC_H_3647bp  ................................................-.........A.   944
MDT_h2_3647bp ................................................-.........A.   944
DNC_h2_3647bp ................................................-.........A.   944
GRP_h2_3647bp ................................................-...........   942
DNC_h1_3647bp ................................................-...........   942
KNG_h1_3647bp ................................................-...........   942
PNK_h1_3647bp ................................................-...........   942
SWT_h1_3647bp ................................................-...........   942
IYK_h1_3647bp ................................................-...........   942
CLM_3647bp    ................................................-...........   942

Consensus     AAATATGAGAAATAGAATTATATTAGATGTCAAAAATTTTTAGAGTTAAACTATTGACAC  1038
OVL_h1_3647bp ....................G....C...........C----------------------  1010
CTR_H_3648bp  ....................G....C............----------------------  1017
LMN_h2_3648bp ....................G....C............----------------------  1017
HYG_H_3647bp  ....................G....C....A.....AA......................   982
MDT_h1_3647bp ....................G....C....A.....AA......................   982
GRP_h1_3647bp .............G......G....C...................G..............   994
IYK_h2_3647bp .............G......G....C...................G..............   994
HSS_h2_3657bp .............G......G....C...................G..............   991
SWT_h2_3657bp .............G......G....C...................G..............   991
KNN_h2_3647bp .............G......G....C...................G..............   993
STS_h1_3647bp .............G......G....C...................G..............   993
PMM_H_3647bp  .............G......G....C...................G..............   993
KSH_h2_3647bp ............................................................  1002
MKK_h2_3647bp ............................................................  1002
PNK_h2_3647bp ...................................T........................  1004
KNN_h1_3647bp ....................................A.......................  1003
STS_h2_3647bp ....................................A.......................  1003
MKK_h1_3647bp ....................................A.......................  1003
LMN_h1_3647bp ....................................A.......................  1003
HSS_h1_3647bp ....................................A.......................  1003
KSH_h1_3647bp ....................................A.......................  1003
KNG_h2_3647bp ............................................................  1004
MRC_H_3647bp  ............................................................  1004
MDT_h2_3647bp ............................................................  1004
DNC_h2_3647bp ............................................................  1004
GRP_h2_3647bp ....................................A.......................  1002
DNC_h1_3647bp ....................................A.......................  1002
KNG_h1_3647bp ....................................A.......................  1002
PNK_h1_3647bp ....................................A.......................  1002
SWT_h1_3647bp ....................................A.......................  1002
IYK_h1_3647bp ....................................A.......................  1002
CLM_3647bp    ....................................A.......................  1002

Consensus     -CTCCCACAATTCTCATAAAAATTATTGTACTCATCTTAATACTAAAAATACACAWTTTA  1097
OVL_h1_3647bp -------------..-.TTTTT..............G..................T....  1056
CTR_H_3648bp  ------------...........C...A...........................T....  1065
LMN_h2_3648bp ------------...........C...A...........................T....  1065
HYG_H_3647bp  C...........T........................................A.T....  1042
MDT_h1_3647bp C...........T........................................A.T....  1042
GRP_h1_3647bp C......................................................T....  1054
IYK_h2_3647bp C......................................................T....  1054
HSS_h2_3657bp C................................G.....................T....  1051
SWT_h2_3657bp C................................G.....................T....  1051
KNN_h2_3647bp C......................................................T....  1053
STS_h1_3647bp C......................................................T....  1053
PMM_H_3647bp  C......................................................T....  1053
KSH_h2_3647bp -......................................................A....  1061
MKK_h2_3647bp -......................................................A....  1061
PNK_h2_3647bp -......................................................A....  1063
KNN_h1_3647bp -......................................................A....  1062
STS_h2_3647bp -......................................................A....  1062
MKK_h1_3647bp -......................................................A....  1062
LMN_h1_3647bp -......................................................A....  1062
HSS_h1_3647bp -......................................................A....  1062
KSH_h1_3647bp -......................................................A....  1062
```

```
KNG_h2_3647bp -......................................................T....  1063
MRC_H_3647bp  -......................................................T....  1063
MDT_h2_3647bp -......................................................T....  1063
DNC_h2_3647bp -......................................................T....  1063
GRP_h2_3647bp -......................................................A....  1061
DNC_h1_3647bp -......................................................A....  1061
KNG_h1_3647bp -......................................................A....  1061
PNK_h1_3647bp -......................................................A....  1061
SWT_h1_3647bp -......................................................A....  1061
IYK_h1_3647bp -......................................................A....  1061
CLM_3647bp    -......................................................A....  1061

Consensus     AAAAATAACCAATCTTCTTATGTTTAAATCTTTCGACTTTCAGAAATTTTATTATTATT-  1156
OVL_h1_3647bp ................T....T.......T.........C...................A  1116
CTR_H_3648bp  .......................................C..........T........-  1124
LMN_h2_3648bp .......................................C..........T........-  1124
HYG_H_3647bp  .............................T.........C..........T........-  1101
MDT_h1_3647bp .............................T.........C..........T........-  1101
GRP_h1_3647bp .......................................C..........T......G.-  1113
IYK_h2_3647bp .......................................C..........T......G.-  1113
HSS_h2_3657bp ..................................A....C..........T......G.-  1110
SWT_h2_3657bp ..................................A....C..........T......G.-  1110
KNN_h2_3647bp .......................................C..........T......G.-  1112
STS_h1_3647bp .......................................C..........T......G.-  1112
PMM_H_3647bp  .......................................C..........T......G.-  1112
KSH_h2_3647bp .................................T.........................-  1120
MKK_h2_3647bp .................................T.........................-  1120
PNK_h2_3647bp ...T.......................................................-  1122
KNN_h1_3647bp .................................T.................A.......-  1121
STS_h2_3647bp .................................T.................A.......-  1121
MKK_h1_3647bp .................................T.................A.......-  1121
LMN_h1_3647bp .................................T.................A.......-  1121
HSS_h1_3647bp .................................T.................A.......-  1121
KSH_h1_3647bp .................................T.................A.......-  1121
KNG_h2_3647bp ...........................................................-  1122
MRC_H_3647bp  ...........................................................-  1122
MDT_h2_3647bp ...........................................................-  1122
DNC_h2_3647bp ...........................................................-  1122
GRP_h2_3647bp ...........................................................-  1120
DNC_h1_3647bp ...........................................................-  1120
KNG_h1_3647bp ...........................................................-  1120
PNK_h1_3647bp ...........................................................-  1120
SWT_h1_3647bp ...........................................................-  1120
IYK_h1_3647bp ...........................................................-  1120
CLM_3647bp    ...........................................................-  1120

Consensus     --AAACCTTGTTTACAATTTTGATATCTCTATTCTTCTCTTTAGACTTTCTATTTCTACA  1214
OVL_h1_3647bp AT...T.......G..------......A......................C...T....  1170
CTR_H_3648bp  --...T.............................................G........  1182
LMN_h2_3648bp --...T.............................................G........  1182
HYG_H_3647bp  --...T.............................................G........  1159
MDT_h1_3647bp --...T.............................................G........  1159
GRP_h1_3647bp --...T.............................................G.....G..  1171
IYK_h2_3647bp --...T.............................................G.....G..  1171
HSS_h2_3657bp --...T.............................................G.....G..  1168
SWT_h2_3657bp --...T.............................................G.....G..  1168
KNN_h2_3647bp --...T...........................G.................G........  1170
STS_h1_3647bp --...T...........................G.................G........  1170
PMM_H_3647bp  --...T...........................G.................G........  1170
KSH_h2_3647bp --.....................................................T....  1178
MKK_h2_3647bp --.....................................................T....  1178
PNK_h2_3647bp --...................................G......................  1180
KNN_h1_3647bp --...................................G......................  1179
STS_h2_3647bp --...................................G......................  1179
MKK_h1_3647bp --...................................G......................  1179
LMN_h1_3647bp --...................................G......................  1179
HSS_h1_3647bp --...................................G......................  1179
KSH_h1_3647bp --...................................G......................  1179
```

```
KNG_h2_3647bp --..........................................................  1180
MRC_H_3647bp  --..........................................................  1180
MDT_h2_3647bp --..........................................................  1180
DNC_h2_3647bp --..........................................................  1180
GRP_h2_3647bp --..................................TG......................  1178
DNC_h1_3647bp --..................................TG......................  1178
KNG_h1_3647bp --..................................TG......................  1178
PNK_h1_3647bp --..................................TG......................  1178
SWT_h1_3647bp --..................................TG......................  1178
IYK_h1_3647bp --..................................TG......................  1178
CLM_3647bp    --..................................TG......................  1178

Consensus     AAACTAAAA-AAAATAGTAACTTCCCTAAAATAAAAATAATTAATATGAAAA--AAAAGA  1271
OVL_h1_3647bp .........A.....T.........................G..........---..T..  1227
CTR_H_3648bp  .........-..........................................--.C....  1239
LMN_h2_3648bp .........-..........................................--.C....  1239
HYG_H_3647bp  .........-..........................................AA......  1218
MDT_h1_3647bp .........-..........................................AA......  1218
GRP_h1_3647bp ....A....A..........................................--......  1229
IYK_h2_3647bp ....A....A..........................................--......  1229
HSS_h2_3657bp ....A....A..........................................AA......  1228
SWT_h2_3657bp ....A....A..........................................AA......  1228
KNN_h2_3647bp ....A....A..........................................--......  1228
STS_h1_3647bp ....A....A..........................................--......  1228
PMM_H_3647bp  ....A....A..........................................--......  1228
KSH_h2_3647bp .........-.....................A.-..................--......  1234
MKK_h2_3647bp .........-.....................A.-..................--......  1234
PNK_h2_3647bp ........--........................-.................--......  1235
KNN_h1_3647bp .........-..........................................--......  1236
STS_h2_3647bp .........-..........................................--......  1236
MKK_h1_3647bp .........-..........................................--......  1236
LMN_h1_3647bp .........-..........................................--......  1236
HSS_h1_3647bp .........-..........................................--......  1236
KSH_h1_3647bp .........-..........................................--......  1236
KNG_h2_3647bp .........-..........................................--......  1237
MRC_H_3647bp  .........-..........................................--......  1237
MDT_h2_3647bp .........-..........................................--......  1237
DNC_h2_3647bp .........-..........................................--......  1237
GRP_h2_3647bp .........-..........................................--......  1235
DNC_h1_3647bp .........-..........................................--......  1235
KNG_h1_3647bp .........-..........................................--......  1235
PNK_h1_3647bp .........-..........................................--......  1235
SWT_h1_3647bp .........-..........................................--......  1235
IYK_h1_3647bp .........-..........................................--......  1235
CLM_3647bp    .........-..........................................--......  1235

Consensus     AAGTAAAAGAAAGAGATGAAACTCCATACTAGTCTCACTTGATTTAAACCTAACTTACTG  1331
OVL_h1_3647bp ...........................T................................  1287
CTR_H_3648bp  ............................................................  1299
LMN_h2_3648bp ............................................................  1299
HYG_H_3647bp  ............................................................  1278
MDT_h1_3647bp ............................................................  1278
GRP_h1_3647bp ............................................................  1289
IYK_h2_3647bp ............................................................  1289
HSS_h2_3657bp .........................................................A..  1288
SWT_h2_3657bp .........................................................A..  1288
KNN_h2_3647bp ............................................................  1288
STS_h1_3647bp ............................................................  1288
PMM_H_3647bp  ............................................................  1288
KSH_h2_3647bp ............................................................  1294
MKK_h2_3647bp ............................................................  1294
PNK_h2_3647bp ............................................................  1295
KNN_h1_3647bp .....................T......................................  1296
STS_h2_3647bp .....................T......................................  1296
MKK_h1_3647bp .....................T......................................  1296
LMN_h1_3647bp .....................T......................................  1296
HSS_h1_3647bp .....................T......................................  1296
KSH_h1_3647bp .....................T......................................  1296
```

```
KNG_h2_3647bp ............................................................ 1297
MRC_H_3647bp  ............................................................ 1297
MDT_h2_3647bp ............................................................ 1297
DNC_h2_3647bp ............................................................ 1297
GRP_h2_3647bp ............................................................ 1295
DNC_h1_3647bp ............................................................ 1295
KNG_h1_3647bp ............................................................ 1295
PNK_h1_3647bp ............................................................ 1295
SWT_h1_3647bp ............................................................ 1295
IYK_h1_3647bp ............................................................ 1295
CLM_3647bp    ............................................................ 1295

Consensus     TGTATATGTATAATTTCTTTAATGAGTTGCTCCTTGAGCATCTTCATAGAAGGAGCTTCA 1391
OVL_h1_3647bp .....................T......A............................... 1347
CTR_H_3648bp  ..........................C.T............................... 1359
LMN_h2_3648bp ..........................C.T............................... 1359
HYG_H_3647bp  .T.......................................................... 1338
MDT_h1_3647bp .T.......................................................... 1338
GRP_h1_3647bp ............C.....C....-.................G.................. 1348
IYK_h2_3647bp ............C.....C....-.................G.................. 1348
HSS_h2_3657bp ............C.....C....-.................G.................. 1347
SWT_h2_3657bp ............C.....C....-.................G.................. 1347
KNN_h2_3647bp ............C.....C....-.................................... 1347
STS_h1_3647bp ............C.....C....-.................................... 1347
PMM_H_3647bp  ............C.....C....-.................................... 1347
KSH_h2_3647bp ...............................................T............ 1354
MKK_h2_3647bp ...............................................T............ 1354
PNK_h2_3647bp ............................................................ 1355
KNN_h1_3647bp ............................................................ 1356
STS_h2_3647bp ............................................................ 1356
MKK_h1_3647bp ............................................................ 1356
LMN_h1_3647bp ............................................................ 1356
HSS_h1_3647bp ............................................................ 1356
KSH_h1_3647bp ............................................................ 1356
KNG_h2_3647bp ............................................................ 1357
MRC_H_3647bp  ............................................................ 1357
MDT_h2_3647bp ............................................................ 1357
DNC_h2_3647bp ............................................................ 1357
GRP_h2_3647bp ............................................................ 1355
DNC_h1_3647bp ............................................................ 1355
KNG_h1_3647bp ............................................................ 1355
PNK_h1_3647bp ............................................................ 1355
SWT_h1_3647bp ............................................................ 1355
IYK_h1_3647bp ............................................................ 1355
CLM_3647bp    ............................................................ 1355

Consensus     AATATTTATGAAGTTTAGAGAAAACAAATTTATAATTTATTACATTGTT----------- 1440
OVL_h1_3647bp ..........................................A......TAAT------- 1400
CTR_H_3648bp  .................................................TAAT------- 1412
LMN_h2_3648bp .................................................TAAT------- 1412
HYG_H_3647bp  ..........................................A......TAATTGTTTAA 1398
MDT_h1_3647bp ..........................................A......TAATTGTTTAA 1398
GRP_h1_3647bp .................................................TAAT------- 1401
IYK_h2_3647bp .................................................TAAT------- 1401
HSS_h2_3657bp .................................................TAAT------- 1400
SWT_h2_3657bp .................................................TAAT------- 1400
KNN_h2_3647bp .................................................TAAT------- 1400
STS_h1_3647bp .................................................TAAT------- 1400
PMM_H_3647bp  .................................................TAAT------- 1400
KSH_h2_3647bp .................................................----------- 1403
MKK_h2_3647bp .................................................----------- 1403
PNK_h2_3647bp .........A.......................................----------- 1404
KNN_h1_3647bp .........A.......................................----------- 1405
STS_h2_3647bp .........A.......................................----------- 1405
MKK_h1_3647bp .........A.......................................----------- 1405
LMN_h1_3647bp .........A.......................................----------- 1405
HSS_h1_3647bp .........A.......................................----------- 1405
KSH_h1_3647bp .........A.......................................----------- 1405
```

```
KNG_h2_3647bp .................................................-----------  1406
MRC_H_3647bp  .................................................-----------  1406
MDT_h2_3647bp .................................................-----------  1406
DNC_h2_3647bp .................................................-----------  1406
GRP_h2_3647bp .........A.......................................-----------  1404
DNC_h1_3647bp .........A.......................................-----------  1404
KNG_h1_3647bp .........A.......................................-----------  1404
PNK_h1_3647bp .........A.......................................-----------  1404
SWT_h1_3647bp .........A.......................................-----------  1404
IYK_h1_3647bp .........A.......................................-----------  1404
CLM_3647bp    .........A.......................................-----------  1404

Consensus     -----TAATTGTTAGCGCATGAGCTTAGTGTAAGATATATTTAGTGGCTTTTAATATGTC  1495
OVL_h1_3647bp -TGTT...........T.........T...G...........G.................  1459
CTR_H_3648bp  -TGTT.......................................................  1471
LMN_h2_3648bp -TGTT.......................................................  1471
HYG_H_3647bp  TTGTT.....................T....T........-A.C..........C.....  1457
MDT_h1_3647bp TTGTT.....................T....T........-A.C..........C.....  1457
GRP_h1_3647bp -TGTT.......................................................  1460
IYK_h2_3647bp -TGTT.......................................................  1460
HSS_h2_3657bp -TGTT.......................................................  1459
SWT_h2_3657bp -TGTT.......................................................  1459
KNN_h2_3647bp -TGTT.......................................................  1459
STS_h1_3647bp -TGTT.......................................................  1459
PMM_H_3647bp  -TGTT.......................................................  1459
KSH_h2_3647bp -----..............................................C........  1458
MKK_h2_3647bp -----..............................................C........  1458
PNK_h2_3647bp -----.......................................................  1459
KNN_h1_3647bp -----.......................................................  1460
STS_h2_3647bp -----.......................................................  1460
MKK_h1_3647bp -----.......................................................  1460
LMN_h1_3647bp -----.......................................................  1460
HSS_h1_3647bp -----.......................................................  1460
KSH_h1_3647bp -----.......................................................  1460
KNG_h2_3647bp -----.............T.........................................  1461
MRC_H_3647bp  -----.............T.........................................  1461
MDT_h2_3647bp -----.............T.........................................  1461
DNC_h2_3647bp -----.............T.........................................  1461
GRP_h2_3647bp -----.......................................................  1459
DNC_h1_3647bp -----.......................................................  1459
KNG_h1_3647bp -----.......................................................  1459
PNK_h1_3647bp -----.......................................................  1459
SWT_h1_3647bp -----.......................................................  1459
IYK_h1_3647bp -----.......................................................  1459
CLM_3647bp    -----.......................................................  1459

Consensus     CTTTAAATTTAAGTACATAATTGATCATTGCTGTACCAACAAAAAA-AA--AAATTAATG  1552
OVL_h1_3647bp .............................................---.--.........  1514
CTR_H_3648bp  .............................................--..--.........  1527
LMN_h2_3648bp .............................................--..--.........  1527
HYG_H_3647bp  ...............-.........T..................G.A..AA.T.......  1516
MDT_h1_3647bp ...............-.........T..................G.A..AA.T.......  1516
GRP_h1_3647bp ......................T.......................A..--.........  1518
IYK_h2_3647bp ......................T.......................A..--.........  1518
HSS_h2_3657bp ......................T.......................A..--.........  1517
SWT_h2_3657bp ......................T.......................A..--.........  1517
KNN_h2_3647bp ......................T.......................A..--.........  1517
STS_h1_3647bp ......................T.......................A..--.........  1517
PMM_H_3647bp  ......................T.......................A..--.........  1517
KSH_h2_3647bp ..............................................A..AT.T.......  1518
MKK_h2_3647bp ..............................................A..AT.T.......  1518
PNK_h2_3647bp .................................A............A..--.........  1517
KNN_h1_3647bp ..............................................-..--.........  1517
STS_h2_3647bp ..............................................-..--.........  1517
MKK_h1_3647bp ..............................................-..--.........  1517
LMN_h1_3647bp ..............................................-..--.........  1517
HSS_h1_3647bp ..............................................-..--.........  1517
KSH_h1_3647bp ..............................................-..--.........  1517
```

```
KNG_h2_3647bp  .............................................--..--.........  1517
MRC_H_3647bp   .............................................--..--.........  1517
MDT_h2_3647bp  .............................................--..--.........  1517
DNC_h2_3647bp  .............................................--..--.........  1517
GRP_h2_3647bp  .................................A............-..--.........  1516
DNC_h1_3647bp  .................................A............-..--.........  1516
KNG_h1_3647bp  .................................A............-..--.........  1516
PNK_h1_3647bp  .................................A............-..--.........  1516
SWT_h1_3647bp  .................................A............-..--.........  1516
IYK_h1_3647bp  .................................A............-..--.........  1516
CLM_3647bp     .................................A............-..--.........  1516

Consensus      TTTCAGGTTTGTGCATAGATGGGAGA----GAGAAAAGAAAAGCTCATAAACATGTACAA  1608
OVL_h1_3647bp  ....................A.....GAGA...................T.G........  1574
CTR_H_3648bp   ....................A.....----...................T..........  1583
LMN_h2_3648bp  ....................A.....----...................T..........  1583
HYG_H_3647bp   ....................A.....----...................T..........  1572
MDT_h1_3647bp  ....................A.....----...................T..........  1572
GRP_h1_3647bp  ....................A.....----...................T......-...  1573
IYK_h2_3647bp  ....................A.....----...................T......-...  1573
HSS_h2_3657bp  ....................A.....----...................T..........  1573
SWT_h2_3657bp  ....................A.....----...................T..........  1573
KNN_h2_3647bp  ....................A.....----...................T..........  1573
STS_h1_3647bp  ....................A.....----...................T..........  1573
PMM_H_3647bp   ....................A.....----...................T..........  1573
KSH_h2_3647bp  ....................A.....----...................T..........  1574
MKK_h2_3647bp  ....................A.....----...................T..........  1574
PNK_h2_3647bp  ..........................----..............................  1573
KNN_h1_3647bp  ..........................----..............................  1573
STS_h2_3647bp  ..........................----..............................  1573
MKK_h1_3647bp  ..........................----..............................  1573
LMN_h1_3647bp  ..........................----..............................  1573
HSS_h1_3647bp  ..........................----..............................  1573
KSH_h1_3647bp  ..........................----..............................  1573
KNG_h2_3647bp  ..........................----............................C.  1573
MRC_H_3647bp   ..........................----............................C.  1573
MDT_h2_3647bp  ..........................----............................C.  1573
DNC_h2_3647bp  ..........................----............................C.  1573
GRP_h2_3647bp  ..........................----..............................  1572
DNC_h1_3647bp  ..........................----..............................  1572
KNG_h1_3647bp  ..........................----..............................  1572
PNK_h1_3647bp  ..........................----..............................  1572
SWT_h1_3647bp  ..........................----..............................  1572
IYK_h1_3647bp  ..........................----..............................  1572
CLM_3647bp     ..........................----..............................  1572

Consensus      GTAAAATAGATCGACAAGAGAGGAAAAAATTAAAATTTAATAAGAGTTTTACGAGATTAA  1668
OVL_h1_3647bp  ..................T..........A.T.T.A........................  1634
CTR_H_3648bp   ........................G.......T...........................  1643
LMN_h2_3648bp  ........................G.......T...........................  1643
HYG_H_3647bp   ....................T.......................T...............  1632
MDT_h1_3647bp  ....................T.......................T...............  1632
GRP_h1_3647bp  ................................T.................G.........  1633
IYK_h2_3647bp  ................................T.................G.........  1633
HSS_h2_3657bp  ................................T.................G.........  1633
SWT_h2_3657bp  ................................T.................G.........  1633
KNN_h2_3647bp  ................................T.................G.........  1633
STS_h1_3647bp  ................................T.................G.........  1633
PMM_H_3647bp   ................................T.................G.........  1633
KSH_h2_3647bp  ................................T.................G.........  1634
MKK_h2_3647bp  ................................T.................G.........  1634
PNK_h2_3647bp  ....................T.......................T...............  1633
KNN_h1_3647bp  ............................................................  1633
STS_h2_3647bp  ............................................................  1633
MKK_h1_3647bp  ............................................................  1633
LMN_h1_3647bp  ............................................................  1633
HSS_h1_3647bp  ............................................................  1633
KSH_h1_3647bp  ............................................................  1633
```

```
KNG_h2_3647bp ................................T.................G......G..  1633
MRC_H_3647bp  ................................T.................G......G..  1633
MDT_h2_3647bp ................................T.................G......G..  1633
DNC_h2_3647bp ................................T.................G......G..  1633
GRP_h2_3647bp ....................T.......................T...............  1632
DNC_h1_3647bp ....................T.......................T...............  1632
KNG_h1_3647bp ....................T.......................T...............  1632
PNK_h1_3647bp ....................T.......................T...............  1632
SWT_h1_3647bp ....................T.......................T...............  1632
IYK_h1_3647bp ....................T.......................T...............  1632
CLM_3647bp    ....................T.......................T...............  1632

Consensus     AGAARGGTGTCAATAGTCGAACCAAAATTTTTAGCTGTRCCCATTTGAGACATTAGCCCA  1728
OVL_h1_3647bp T...G....C............T.T.....C..T....A......C........G.....  1694
CTR_H_3648bp  C...G...........................T.....A...............TT....  1703
LMN_h2_3648bp C...G...........................T.....A...............TT....  1703
HYG_H_3647bp  ....A.................................G.....................  1692
MDT_h1_3647bp ....A.................................G.....................  1692
GRP_h1_3647bp C...G.................................A..G..................  1693
IYK_h2_3647bp C...G.................................A..G..................  1693
HSS_h2_3657bp C...G.................................A.....................  1693
SWT_h2_3657bp C...G.................................A.....................  1693
KNN_h2_3647bp C...G.................................A.....................  1693
STS_h1_3647bp C...G.................................A.....................  1693
PMM_H_3647bp  C...G.................................A.....................  1693
KSH_h2_3647bp C...G...A..G..........................A.....................  1694
MKK_h2_3647bp C...G...A..G..........................A.....................  1694
PNK_h2_3647bp ....A.................................G.....................  1693
KNN_h1_3647bp ....A.................................G.....................  1693
STS_h2_3647bp ....A.................................G.....................  1693
MKK_h1_3647bp ....A.................................G.....................  1693
LMN_h1_3647bp ....A.................................G.....................  1693
HSS_h1_3647bp ....A.................................G.....................  1693
KSH_h1_3647bp ....A.................................G.....................  1693
KNG_h2_3647bp C...G..CA.............................A.....................  1693
MRC_H_3647bp  C...G..CA.............................A.....................  1693
MDT_h2_3647bp C...G..CA.............................A.....................  1693
DNC_h2_3647bp C...G..CA.............................A.....................  1693
GRP_h2_3647bp ....A.................................G.....................  1692
DNC_h1_3647bp ....A.................................G.....................  1692
KNG_h1_3647bp ....A.................................G.....................  1692
PNK_h1_3647bp ....A.................................G.....................  1692
SWT_h1_3647bp ....A.................................G.....................  1692
IYK_h1_3647bp ....A.................................G.....................  1692
CLM_3647bp    ....A.................................G.....................  1692

Consensus     ATACAATGGTATTACAAGGCCCAATAAGAAACAAGATTGATCCACGACTATAACCCACCC  1788
OVL_h1_3647bp .................T.........................................G  1754
CTR_H_3648bp  ..............................G.............................  1763
LMN_h2_3648bp ..............................G.............................  1763
HYG_H_3647bp  ............................................................  1752
MDT_h1_3647bp ............................................................  1752
GRP_h1_3647bp ......................................T.....................  1753
IYK_h2_3647bp ......................................T.....................  1753
HSS_h2_3657bp .........................................................G..  1753
SWT_h2_3657bp .........................................................G..  1753
KNN_h2_3647bp .........................................................G..  1753
STS_h1_3647bp .........................................................G..  1753
PMM_H_3647bp  .........................................................G..  1753
KSH_h2_3647bp ............................................................  1754
MKK_h2_3647bp ............................................................  1754
PNK_h2_3647bp ............................................................  1753
KNN_h1_3647bp ............................................................  1753
STS_h2_3647bp ............................................................  1753
MKK_h1_3647bp ............................................................  1753
LMN_h1_3647bp ............................................................  1753
HSS_h1_3647bp ............................................................  1753
KSH_h1_3647bp ............................................................  1753
```

```
KNG_h2_3647bp ............................................................  1753
MRC_H_3647bp  ............................................................  1753
MDT_h2_3647bp ............................................................  1753
DNC_h2_3647bp ............................................................  1753
GRP_h2_3647bp ............................................................  1752
DNC_h1_3647bp ............................................................  1752
KNG_h1_3647bp ............................................................  1752
PNK_h1_3647bp ............................................................  1752
SWT_h1_3647bp ............................................................  1752
IYK_h1_3647bp ............................................................  1752
CLM_3647bp    ............................................................  1752

Consensus     GAGCAAACACCGACCCGATAGCTCCAACCCGACGTGGGCAAAAATCCCGTGAAACGCATT  1848
OVL_h1_3647bp ....-.......................................................  1813
CTR_H_3648bp  ....-.......................................................  1822
LMN_h2_3648bp ....-.......................................................  1822
HYG_H_3647bp  ............................................................  1812
MDT_h1_3647bp ............................................................  1812
GRP_h1_3647bp ....-.......................................................  1812
IYK_h2_3647bp ....-.......................................................  1812
HSS_h2_3657bp ....-............................A..........................  1812
SWT_h2_3657bp ....-............................A..........................  1812
KNN_h2_3647bp ....-............................A..........................  1812
STS_h1_3647bp ....-............................A..........................  1812
PMM_H_3647bp  ....-............................A..........................  1812
KSH_h2_3647bp ....-.................G.............A.......................  1813
MKK_h2_3647bp ....-.................G.............A.......................  1813
PNK_h2_3647bp ............................................................  1813
KNN_h1_3647bp ............................................................  1813
STS_h2_3647bp ............................................................  1813
MKK_h1_3647bp ............................................................  1813
LMN_h1_3647bp ............................................................  1813
HSS_h1_3647bp ............................................................  1813
KSH_h1_3647bp ............................................................  1813
KNG_h2_3647bp ............................................................  1813
MRC_H_3647bp  ............................................................  1813
MDT_h2_3647bp ............................................................  1813
DNC_h2_3647bp ............................................................  1813
GRP_h2_3647bp ............................................................  1812
DNC_h1_3647bp ............................................................  1812
KNG_h1_3647bp ............................................................  1812
PNK_h1_3647bp ............................................................  1812
SWT_h1_3647bp ............................................................  1812
IYK_h1_3647bp ............................................................  1812
CLM_3647bp    ............................................................  1812

Consensus     AATGCTGATGGCGTCACTAATGCATTAATTCCTAGCCC-CCAATTCTCGTGAAGCCATAA  1907
OVL_h1_3647bp ...................................T..-.....................  1872
CTR_H_3648bp  ......................................C.....................  1882
LMN_h2_3648bp ......................................C.....................  1882
HYG_H_3647bp  ......................................-.....................  1871
MDT_h1_3647bp ......................................-.....................  1871
GRP_h1_3647bp ......................................C.....................  1872
IYK_h2_3647bp ......................................C.....................  1872
HSS_h2_3657bp .................C....................C.....................  1872
SWT_h2_3657bp .................C....................C.....................  1872
KNN_h2_3647bp .................C....................C.....................  1872
STS_h1_3647bp .................C....................C.....................  1872
PMM_H_3647bp  .................C....................C.....................  1872
KSH_h2_3647bp ......................................-.....................  1872
MKK_h2_3647bp ......................................-.....................  1872
PNK_h2_3647bp ......................................-.....................  1872
KNN_h1_3647bp ......................................-.....................  1872
STS_h2_3647bp ......................................-.....................  1872
MKK_h1_3647bp ......................................-.....................  1872
LMN_h1_3647bp ......................................-.....................  1872
HSS_h1_3647bp ......................................-.....................  1872
KSH_h1_3647bp ......................................-.....................  1872
```

```
KNG_h2_3647bp ......................................-.....................  1872
MRC_H_3647bp  ......................................-.....................  1872
MDT_h2_3647bp ......................................-.....................  1872
DNC_h2_3647bp ......................................-.....................  1872
GRP_h2_3647bp ......................................-.....................  1871
DNC_h1_3647bp ......................................-.....................  1871
KNG_h1_3647bp ......................................-.....................  1871
PNK_h1_3647bp ......................................-.....................  1871
SWT_h1_3647bp ......................................-.....................  1871
IYK_h1_3647bp ......................................-.....................  1871
CLM_3647bp    ......................................-.....................  1871

Consensus     CTAGTTTCACTTCATTCCATGCCACAGCCGCTCTTTTTGTCACTGCATCTCATTAATACA  1967
OVL_h1_3647bp ................T.....A......T...........G..................  1932
CTR_H_3648bp  ...............----------....T..............................  1932
LMN_h2_3648bp ...............----------....T..............................  1932
HYG_H_3647bp  ............................................................  1931
MDT_h1_3647bp ............................................................  1931
GRP_h1_3647bp ....................A.A......T..............................  1932
IYK_h2_3647bp ....................A.A......T..............................  1932
HSS_h2_3657bp ......................A..G...T..............T...............  1932
SWT_h2_3657bp ......................A..G...T..............T...............  1932
KNN_h2_3647bp ......................A..G...T..............T...............  1932
STS_h1_3647bp ......................A..G...T..............T...............  1932
PMM_H_3647bp  ......................A..G...T..............T...............  1932
KSH_h2_3647bp .........................................G..................  1932
MKK_h2_3647bp .........................................G..................  1932
PNK_h2_3647bp ....C.......................................................  1932
KNN_h1_3647bp ............................................................  1932
STS_h2_3647bp ............................................................  1932
MKK_h1_3647bp ............................................................  1932
LMN_h1_3647bp ............................................................  1932
HSS_h1_3647bp ............................................................  1932
KSH_h1_3647bp ............................................................  1932
KNG_h2_3647bp .................................C............G.............  1932
MRC_H_3647bp  .................................C............G.............  1932
MDT_h2_3647bp .................................C............G.............  1932
DNC_h2_3647bp .................................C............G.............  1932
GRP_h2_3647bp ............................................................  1931
DNC_h1_3647bp ............................................................  1931
KNG_h1_3647bp ............................................................  1931
PNK_h1_3647bp ............................................................  1931
SWT_h1_3647bp ............................................................  1931
IYK_h1_3647bp ............................................................  1931
CLM_3647bp    ............................................................  1931

Consensus     CTGACCCATTTCATTAACTCCACCGTCACCTCTATATAAATCAAATGCAAGCCCAGTTAA  2027
OVL_h1_3647bp ....................................................T.......  1992
CTR_H_3648bp  ..............................................T.............  1992
LMN_h2_3648bp ..............................................T.............  1992
HYG_H_3647bp  ............................................................  1991
MDT_h1_3647bp ............................................................  1991
GRP_h1_3647bp ...........T................................................  1992
IYK_h2_3647bp ...........T................................................  1992
HSS_h2_3657bp ...........T................................................  1992
SWT_h2_3657bp ...........T................................................  1992
KNN_h2_3647bp ...........T................................................  1992
STS_h1_3647bp ...........T................................................  1992
PMM_H_3647bp  ...........T................................................  1992
KSH_h2_3647bp ............................................................  1992
MKK_h2_3647bp ............................................................  1992
PNK_h2_3647bp ............................................................  1992
KNN_h1_3647bp ............................................................  1992
STS_h2_3647bp ............................................................  1992
MKK_h1_3647bp ............................................................  1992
LMN_h1_3647bp ............................................................  1992
HSS_h1_3647bp ............................................................  1992
KSH_h1_3647bp ............................................................  1992
```

```
KNG_h2_3647bp ............................................................  1992
MRC_H_3647bp  ............................................................  1992
MDT_h2_3647bp ............................................................  1992
DNC_h2_3647bp ............................................................  1992
GRP_h2_3647bp ............................................................  1991
DNC_h1_3647bp ............................................................  1991
KNG_h1_3647bp ............................................................  1991
PNK_h1_3647bp ............................................................  1991
SWT_h1_3647bp ............................................................  1991
IYK_h1_3647bp ............................................................  1991
CLM_3647bp    ............................................................  1991

Consensus     -AGGAAAACAAATGCGCGCTTACAAAAAATTAAAAAAATGGCACAACATTTCTCAAAACG  2086
OVL_h1_3647bp -..............A............................................  2051
CTR_H_3648bp  -..............A.C..........................................  2051
LMN_h2_3648bp -..............A.C..........................................  2051
HYG_H_3647bp  A...........................................................  2051
MDT_h1_3647bp A...........................................................  2051
GRP_h1_3647bp -..............A.C..........................................  2051
IYK_h2_3647bp -..............A.C..........................................  2051
HSS_h2_3657bp -..............A............................................  2051
SWT_h2_3657bp -..............A............................................  2051
KNN_h2_3647bp -..............A............................................  2051
STS_h1_3647bp -..............A............................................  2051
PMM_H_3647bp  -..............A............................................  2051
KSH_h2_3647bp -........T..................................................  2051
MKK_h2_3647bp -........T..................................................  2051
PNK_h2_3647bp -.......T...................................................  2051
KNN_h1_3647bp -.......T...................................................  2051
STS_h2_3647bp -.......T...................................................  2051
MKK_h1_3647bp -.......T...................................................  2051
LMN_h1_3647bp -.......T...................................................  2051
HSS_h1_3647bp -.......T...................................................  2051
KSH_h1_3647bp -.......T...................................................  2051
KNG_h2_3647bp -..................A........................................  2051
MRC_H_3647bp  -..................A........................................  2051
MDT_h2_3647bp -..................A........................................  2051
DNC_h2_3647bp -..................A........................................  2051
GRP_h2_3647bp A...........................................................  2051
DNC_h1_3647bp A...........................................................  2051
KNG_h1_3647bp A...........................................................  2051
PNK_h1_3647bp A...........................................................  2051
SWT_h1_3647bp A...........................................................  2051
IYK_h1_3647bp A...........................................................  2051
CLM_3647bp    A...........................................................  2051

Consensus     AACTCCTTTGGTTCTGGCCTTCATTTGTGTTTTRCCATTTGCGAGGCCTTTCACGGTCAT  2146
OVL_h1_3647bp .................................G..........................  2111
CTR_H_3648bp  .................................G..........................  2111
LMN_h2_3648bp .................................G..........................  2111
HYG_H_3647bp  ...A...C.....T...................A........T.................  2111
MDT_h1_3647bp ...A...C.....T...................A........T.................  2111
GRP_h1_3647bp .................................G..........................  2111
IYK_h2_3647bp .................................G..........................  2111
HSS_h2_3657bp .................................G..........................  2111
SWT_h2_3657bp .................................G..........................  2111
KNN_h2_3647bp .................................G..........................  2111
STS_h1_3647bp .................................G..........................  2111
PMM_H_3647bp  .................................G..........................  2111
KSH_h2_3647bp ...............A.................G..........................  2111
MKK_h2_3647bp ...............A.................G..........................  2111
PNK_h2_3647bp .................................A...........T..............  2111
KNN_h1_3647bp .................................A...........T..............  2111
STS_h2_3647bp .................................A...........T..............  2111
MKK_h1_3647bp .................................A...........T..............  2111
LMN_h1_3647bp .................................A...........T..............  2111
HSS_h1_3647bp .................................A...........T..............  2111
KSH_h1_3647bp .................................A...........T..............  2111
```

```
KNG_h2_3647bp ......C..........................G..........................  2111
MRC_H_3647bp  ......C..........................G..........................  2111
MDT_h2_3647bp ......C..........................G..........................  2111
DNC_h2_3647bp ......C..........................G..........................  2111
GRP_h2_3647bp ...A...C.....T...................A........T.................  2111
DNC_h1_3647bp ...A...C.....T...................A........T.................  2111
KNG_h1_3647bp ...A...C.....T...................A........T.................  2111
PNK_h1_3647bp ...A...C.....T...................A........T.................  2111
SWT_h1_3647bp ...A...C.....T...................A........T.................  2111
IYK_h1_3647bp ...A...C.....T...................A........T.................  2111
CLM_3647bp    ...A...C.....T...................A........T.................  2111

Consensus     AATGCAAGATTCCGGCGGATCCTCGGGCCTTGTCGATGGGCCGCAGACTGGGTTCTCGAT  2206
OVL_h1_3647bp ............................................................  2171
CTR_H_3648bp  .....C......................................................  2171
LMN_h2_3648bp .....C......................................................  2171
HYG_H_3647bp  ............................................................  2171
MDT_h1_3647bp ............................................................  2171
GRP_h1_3647bp ............................................................  2171
IYK_h2_3647bp ............................................................  2171
HSS_h2_3657bp ................................................C...........  2171
SWT_h2_3657bp ................................................C...........  2171
KNN_h2_3647bp ................................................C...........  2171
STS_h1_3647bp ................................................C...........  2171
PMM_H_3647bp  ................................................C...........  2171
KSH_h2_3647bp C...........................................................  2171
MKK_h2_3647bp C...........................................................  2171
PNK_h2_3647bp ..........................................A.................  2171
KNN_h1_3647bp ..........................................A.................  2171
STS_h2_3647bp ..........................................A.................  2171
MKK_h1_3647bp ..........................................A.................  2171
LMN_h1_3647bp ..........................................A.................  2171
HSS_h1_3647bp ..........................................A.................  2171
KSH_h1_3647bp ..........................................A.................  2171
KNG_h2_3647bp C...........................................................  2171
MRC_H_3647bp  C...........................................................  2171
MDT_h2_3647bp C...........................................................  2171
DNC_h2_3647bp C...........................................................  2171
GRP_h2_3647bp ............................................................  2171
DNC_h1_3647bp ............................................................  2171
KNG_h1_3647bp ............................................................  2171
PNK_h1_3647bp ............................................................  2171
SWT_h1_3647bp ............................................................  2171
IYK_h1_3647bp ............................................................  2171
CLM_3647bp    ............................................................  2171

Consensus     GAACAAGGATGGAGCCCGAACCGAGCCCAAGGAGCAAGAGGCTGTCTACGACATAATGCG  2266
OVL_h1_3647bp ........................T...................................  2231
CTR_H_3648bp  ........................T...................................  2231
LMN_h2_3648bp ........................T...................................  2231
HYG_H_3647bp  ............................................................  2231
MDT_h1_3647bp ............................................................  2231
GRP_h1_3647bp ........................T...................................  2231
IYK_h2_3647bp ........................T...................................  2231
HSS_h2_3657bp ..G.....................T...................................  2231
SWT_h2_3657bp ..G.....................T...................................  2231
KNN_h2_3647bp ..G.....................T...................................  2231
STS_h1_3647bp ..G.....................T...................................  2231
PMM_H_3647bp  ..G.....................T...................................  2231
KSH_h2_3647bp .........................T..................................  2231
MKK_h2_3647bp .........................T..................................  2231
PNK_h2_3647bp ............................................................  2231
KNN_h1_3647bp ............................................................  2231
STS_h2_3647bp ............................................................  2231
MKK_h1_3647bp ............................................................  2231
LMN_h1_3647bp ............................................................  2231
HSS_h1_3647bp ............................................................  2231
KSH_h1_3647bp ............................................................  2231
```

```
KNG_h2_3647bp ............G............T..................................  2231
MRC_H_3647bp  ............G............T..................................  2231
MDT_h2_3647bp ............G............T..................................  2231
DNC_h2_3647bp ............G............T..................................  2231
GRP_h2_3647bp ............................................................  2231
DNC_h1_3647bp ............................................................  2231
KNG_h1_3647bp ............................................................  2231
PNK_h1_3647bp ............................................................  2231
SWT_h1_3647bp ............................................................  2231
IYK_h1_3647bp ............................................................  2231
CLM_3647bp    ............................................................  2231

Consensus     GGCCACAGGAAACGATTGGGCCACCGAAATTCCCGACGTGTGTCGCGGCCGTTGGCACGG  2326
OVL_h1_3647bp .............................................T..............  2291
CTR_H_3648bp  ......G........C............................................  2291
LMN_h2_3648bp ......G........C............................................  2291
HYG_H_3647bp  ............................................................  2291
MDT_h1_3647bp ............................................................  2291
GRP_h1_3647bp .............................................T..............  2291
IYK_h2_3647bp .............................................T..............  2291
HSS_h2_3657bp ............................................................  2291
SWT_h2_3657bp ............................................................  2291
KNN_h2_3647bp ............................................................  2291
STS_h1_3647bp ............................................................  2291
PMM_H_3647bp  ............................................................  2291
KSH_h2_3647bp ............................................................  2291
MKK_h2_3647bp ............................................................  2291
PNK_h2_3647bp ............................................................  2291
KNN_h1_3647bp ............................................................  2291
STS_h2_3647bp ............................................................  2291
MKK_h1_3647bp ............................................................  2291
LMN_h1_3647bp ............................................................  2291
HSS_h1_3647bp ............................................................  2291
KSH_h1_3647bp ............................................................  2291
KNG_h2_3647bp ............................................................  2291
MRC_H_3647bp  ............................................................  2291
MDT_h2_3647bp ............................................................  2291
DNC_h2_3647bp ............................................................  2291
GRP_h2_3647bp ............................................................  2291
DNC_h1_3647bp ............................................................  2291
KNG_h1_3647bp ............................................................  2291
PNK_h1_3647bp ............................................................  2291
SWT_h1_3647bp ............................................................  2291
IYK_h1_3647bp ............................................................  2291
CLM_3647bp    ............................................................  2291

Consensus     TATCGAGTGCATGCCGGATAAAGAAAACGTGTACCACGTGGTGTCGTTGATGTTTGGAGC  2386
OVL_h1_3647bp ............................................................  2351
CTR_H_3648bp  ............................................................  2351
LMN_h2_3648bp ............................................................  2351
HYG_H_3647bp  C...........................................................  2351
MDT_h1_3647bp C...........................................................  2351
GRP_h1_3647bp ..............................C.............................  2351
IYK_h2_3647bp ..............................C.............................  2351
HSS_h2_3657bp ............................................................  2351
SWT_h2_3657bp ............................................................  2351
KNN_h2_3647bp ............................................................  2351
STS_h1_3647bp ............................................................  2351
PMM_H_3647bp  ............................................................  2351
KSH_h2_3647bp C...........................................................  2351
MKK_h2_3647bp C...........................................................  2351
PNK_h2_3647bp ............................................................  2351
KNN_h1_3647bp ............................................................  2351
STS_h2_3647bp ............................................................  2351
MKK_h1_3647bp ............................................................  2351
LMN_h1_3647bp ............................................................  2351
HSS_h1_3647bp ............................................................  2351
KSH_h1_3647bp ............................................................  2351
```

```
KNG_h2_3647bp C...........................................................  2351
MRC_H_3647bp  C...........................................................  2351
MDT_h2_3647bp C...........................................................  2351
DNC_h2_3647bp C...........................................................  2351
GRP_h2_3647bp C...........................................................  2351
DNC_h1_3647bp C...........................................................  2351
KNG_h1_3647bp C...........................................................  2351
PNK_h1_3647bp C...........................................................  2351
SWT_h1_3647bp C...........................................................  2351
IYK_h1_3647bp C...........................................................  2351
CLM_3647bp    C...........................................................  2351

Consensus     TTTATCTGATGACACTGCTTTTCCGACTTGTGATCCGACCCGGTCTCATATCTCACGATC  2446
OVL_h1_3647bp ............................................................  2411
CTR_H_3648bp  ......C........G...........A...........T....................  2411
LMN_h2_3648bp ......C........G...........A...........T....................  2411
HYG_H_3647bp  ............................................................  2411
MDT_h1_3647bp ............................................................  2411
GRP_h1_3647bp ......C........G...........A................................  2411
IYK_h2_3647bp ......C........G...........A................................  2411
HSS_h2_3657bp .....................................G......................  2411
SWT_h2_3657bp .....................................G......................  2411
KNN_h2_3647bp .....................................G......................  2411
STS_h1_3647bp .....................................G......................  2411
PMM_H_3647bp  .....................................G......................  2411
KSH_h2_3647bp ............................................................  2411
MKK_h2_3647bp ............................................................  2411
PNK_h2_3647bp ......C........G...........A................................  2411
KNN_h1_3647bp ......C........G...........A.................C..............  2411
STS_h2_3647bp ......C........G...........A.................C..............  2411
MKK_h1_3647bp ......C........G...........A.................C..............  2411
LMN_h1_3647bp ......C........G...........A.................C..............  2411
HSS_h1_3647bp ......C........G...........A.................C..............  2411
KSH_h1_3647bp ......C........G...........A.................C..............  2411
KNG_h2_3647bp ............................................................  2411
MRC_H_3647bp  ............................................................  2411
MDT_h2_3647bp ............................................................  2411
DNC_h2_3647bp ............................................................  2411
GRP_h2_3647bp ............................................................  2411
DNC_h1_3647bp ............................................................  2411
KNG_h1_3647bp ............................................................  2411
PNK_h1_3647bp ............................................................  2411
SWT_h1_3647bp ............................................................  2411
IYK_h1_3647bp ............................................................  2411
CLM_3647bp    ............................................................  2411

Consensus     CATCACCAAGCTTCCTTACCTTAGAACTTTGTTCTTTTACCGTTGCTTCACTCACAATCC  2506
OVL_h1_3647bp ............................................................  2471
CTR_H_3648bp  ............................................................  2471
LMN_h2_3648bp ............................................................  2471
HYG_H_3647bp  ............................................................  2471
MDT_h1_3647bp ............................................................  2471
GRP_h1_3647bp ......G.....................................................  2471
IYK_h2_3647bp ......G.....................................................  2471
HSS_h2_3657bp ......G.....................................................  2471
SWT_h2_3657bp ......G.....................................................  2471
KNN_h2_3647bp ......G.....................................................  2471
STS_h1_3647bp ......G.....................................................  2471
PMM_H_3647bp  ......G.....................................................  2471
KSH_h2_3647bp ............................................................  2471
MKK_h2_3647bp ............................................................  2471
PNK_h2_3647bp ............................................................  2471
KNN_h1_3647bp ............................................................  2471
STS_h2_3647bp ............................................................  2471
MKK_h1_3647bp ............................................................  2471
LMN_h1_3647bp ............................................................  2471
HSS_h1_3647bp ............................................................  2471
KSH_h1_3647bp ............................................................  2471
```

```
KNG_h2_3647bp .......................A....................................  2471
MRC_H_3647bp  .......................A....................................  2471
MDT_h2_3647bp .......................A....................................  2471
DNC_h2_3647bp .......................A....................................  2471
GRP_h2_3647bp ............................................................  2471
DNC_h1_3647bp ............................................................  2471
KNG_h1_3647bp ............................................................  2471
PNK_h1_3647bp ............................................................  2471
SWT_h1_3647bp ............................................................  2471
IYK_h1_3647bp ............................................................  2471
CLM_3647bp    ............................................................  2471

Consensus     CCAGCCAATCCCAGCATTTTTAGGTCAATTGGGCCAAACGTTGCAAACTTTAGTGCTTAG  2566
OVL_h1_3647bp ..................C.........................................  2531
CTR_H_3648bp  ..........................................A..............C..  2531
LMN_h2_3648bp ..........................................A..............C..  2531
HYG_H_3647bp  ............................................................  2531
MDT_h1_3647bp ............................................................  2531
GRP_h1_3647bp ..........................................A..............C..  2531
IYK_h2_3647bp ..........................................A..............C..  2531
HSS_h2_3657bp ..........................................A..............C..  2531
SWT_h2_3657bp ..........................................A..............C..  2531
KNN_h2_3647bp ..........................................A..............C..  2531
STS_h1_3647bp ..........................................A..............C..  2531
PMM_H_3647bp  ..........................................A..............C..  2531
KSH_h2_3647bp .........................................................C..  2531
MKK_h2_3647bp .........................................................C..  2531
PNK_h2_3647bp ..................C.........................................  2531
KNN_h1_3647bp ..................C.........................................  2531
STS_h2_3647bp ..................C.........................................  2531
MKK_h1_3647bp ..................C.........................................  2531
LMN_h1_3647bp ..................C.........................................  2531
HSS_h1_3647bp ..................C.........................................  2531
KSH_h1_3647bp ..................C.........................................  2531
KNG_h2_3647bp ..................C.........................................  2531
MRC_H_3647bp  ..................C.........................................  2531
MDT_h2_3647bp ..................C.........................................  2531
DNC_h2_3647bp ..................C.........................................  2531
GRP_h2_3647bp ............................................................  2531
DNC_h1_3647bp ............................................................  2531
KNG_h1_3647bp ............................................................  2531
PNK_h1_3647bp ............................................................  2531
SWT_h1_3647bp ............................................................  2531
IYK_h1_3647bp ............................................................  2531
CLM_3647bp    ............................................................  2531

Consensus     AGAAAACGGGAACGTGGGTCCAATTCCTAGCGAATTAGGAAATCTCACCCGTTTAAAAGT  2626
OVL_h1_3647bp ............................................................  2591
CTR_H_3648bp  ......T......................TT.............................  2591
LMN_h2_3648bp ......T......................TT.............................  2591
HYG_H_3647bp  ..............................T.............................  2591
MDT_h1_3647bp ..............................T.............................  2591
GRP_h1_3647bp ............................................................  2591
IYK_h2_3647bp ............................................................  2591
HSS_h2_3657bp ............................................................  2591
SWT_h2_3657bp ............................................................  2591
KNN_h2_3647bp ............................................................  2591
STS_h1_3647bp ............................................................  2591
PMM_H_3647bp  ............................................................  2591
KSH_h2_3647bp ......T.......................T.............................  2591
MKK_h2_3647bp ......T.......................T.............................  2591
PNK_h2_3647bp ..............................T.............................  2591
KNN_h1_3647bp ............................................................  2591
STS_h2_3647bp ............................................................  2591
MKK_h1_3647bp ............................................................  2591
LMN_h1_3647bp ............................................................  2591
HSS_h1_3647bp ............................................................  2591
KSH_h1_3647bp ............................................................  2591
```

```
KNG_h2_3647bp  .......................................G....................  2591
MRC_H_3647bp   .......................................G....................  2591
MDT_h2_3647bp  .......................................G....................  2591
DNC_h2_3647bp  .......................................G....................  2591
GRP_h2_3647bp  ..............................T.......C.....................  2591
DNC_h1_3647bp  ..............................T.......C.....................  2591
KNG_h1_3647bp  ..............................T.......C.....................  2591
PNK_h1_3647bp  ..............................T.......C.....................  2591
SWT_h1_3647bp  ..............................T.......C.....................  2591
IYK_h1_3647bp  ..............................T.......C.....................  2591
CLM_3647bp     ..............................T.......C.....................  2591

Consensus      CCTTGATCTTCACAAAAACAATCTCAACGGTTCGATTCCAGTTTCTTTAGGCAGGATCAA  2686
OVL_h1_3647bp  ..................T.........................................  2651
CTR_H_3648bp   ...................................................G........  2651
LMN_h2_3648bp  ...................................................G........  2651
HYG_H_3647bp   ............................................................  2651
MDT_h1_3647bp  ............................................................  2651
GRP_h1_3647bp  ............................................................  2651
IYK_h2_3647bp  ............................................................  2651
HSS_h2_3657bp  ............................................................  2651
SWT_h2_3657bp  ............................................................  2651
KNN_h2_3647bp  ............................................................  2651
STS_h1_3647bp  ............................................................  2651
PMM_H_3647bp   ............................................................  2651
KSH_h2_3647bp  ............................................................  2651
MKK_h2_3647bp  ............................................................  2651
PNK_h2_3647bp  ............................................................  2651
KNN_h1_3647bp  ............................................................  2651
STS_h2_3647bp  ............................................................  2651
MKK_h1_3647bp  ............................................................  2651
LMN_h1_3647bp  ............................................................  2651
HSS_h1_3647bp  ............................................................  2651
KSH_h1_3647bp  ............................................................  2651
KNG_h2_3647bp  ............................................................  2651
MRC_H_3647bp   ............................................................  2651
MDT_h2_3647bp  ............................................................  2651
DNC_h2_3647bp  ............................................................  2651
GRP_h2_3647bp  ............................................................  2651
DNC_h1_3647bp  ............................................................  2651
KNG_h1_3647bp  ............................................................  2651
PNK_h1_3647bp  ............................................................  2651
SWT_h1_3647bp  ............................................................  2651
IYK_h1_3647bp  ............................................................  2651
CLM_3647bp     ............................................................  2651

Consensus      CGGTCTGAGGTCGTTAGATTTGAGTGGGAACAAATTAACCGGTTCGATACCCAGTATAAG  2746
OVL_h1_3647bp  ............................................................  2711
CTR_H_3648bp   ...............G.......................................T....  2711
LMN_h2_3648bp  ...............G.......................................T....  2711
HYG_H_3647bp   .......................................G....................  2711
MDT_h1_3647bp  .......................................G....................  2711
GRP_h1_3647bp  ............................................................  2711
IYK_h2_3647bp  ............................................................  2711
HSS_h2_3657bp  ............................................................  2711
SWT_h2_3657bp  ............................................................  2711
KNN_h2_3647bp  ............................................................  2711
STS_h1_3647bp  ............................................................  2711
PMM_H_3647bp   ............................................................  2711
KSH_h2_3647bp  ............................................................  2711
MKK_h2_3647bp  ............................................................  2711
PNK_h2_3647bp  ............................................................  2711
KNN_h1_3647bp  ...............G....................................T.......  2711
STS_h2_3647bp  ...............G....................................T.......  2711
MKK_h1_3647bp  ...............G....................................T.......  2711
LMN_h1_3647bp  ...............G....................................T.......  2711
HSS_h1_3647bp  ...............G....................................T.......  2711
KSH_h1_3647bp  ...............G....................................T.......  2711
```

```
KNG_h2_3647bp ...............G............................................  2711
MRC_H_3647bp  ...............G............................................  2711
MDT_h2_3647bp ...............G............................................  2711
DNC_h2_3647bp ...............G............................................  2711
GRP_h2_3647bp ............................................................  2711
DNC_h1_3647bp ............................................................  2711
KNG_h1_3647bp ............................................................  2711
PNK_h1_3647bp ............................................................  2711
SWT_h1_3647bp ............................................................  2711
IYK_h1_3647bp ............................................................  2711
CLM_3647bp    ............................................................  2711

Consensus     CTTCCCAGTTCTAAATGTTTTAGACTTGAACCAAAACCTTCTTATGGGTCCGATCCCATC  2806
OVL_h1_3647bp ...................A........................................  2771
CTR_H_3648bp  ............................................................  2771
LMN_h2_3648bp ............................................................  2771
HYG_H_3647bp  ..................................................T.........  2771
MDT_h1_3647bp ..................................................T.........  2771
GRP_h1_3647bp ...................................................C........  2771
IYK_h2_3647bp ...................................................C........  2771
HSS_h2_3657bp ...................................................C........  2771
SWT_h2_3657bp ...................................................C........  2771
KNN_h2_3647bp ...................................................C........  2771
STS_h1_3647bp ...................................................C........  2771
PMM_H_3647bp  ...................................................C........  2771
KSH_h2_3647bp ............................................................  2771
MKK_h2_3647bp ............................................................  2771
PNK_h2_3647bp ...................................................A........  2771
KNN_h1_3647bp ............................................................  2771
STS_h2_3647bp ............................................................  2771
MKK_h1_3647bp ............................................................  2771
LMN_h1_3647bp ............................................................  2771
HSS_h1_3647bp ............................................................  2771
KSH_h1_3647bp ............................................................  2771
KNG_h2_3647bp ..........................................................A.  2771
MRC_H_3647bp  ..........................................................A.  2771
MDT_h2_3647bp ..........................................................A.  2771
DNC_h2_3647bp ..........................................................A.  2771
GRP_h2_3647bp ..................................................T.........  2771
DNC_h1_3647bp ..................................................T.........  2771
KNG_h1_3647bp ..................................................T.........  2771
PNK_h1_3647bp ..................................................T.........  2771
SWT_h1_3647bp ..................................................T.........  2771
IYK_h1_3647bp ..................................................T.........  2771
CLM_3647bp    ..................................................T.........  2771

Consensus     TAGTCTCGGAACGTGCCATTCTTTAATCAAAATCGATTTTAGCCATAATCGCCTCACCGG  2866
OVL_h1_3647bp ............................................................  2831
CTR_H_3648bp  ............................................................  2831
LMN_h2_3648bp ............................................................  2831
HYG_H_3647bp  ............................................................  2831
MDT_h1_3647bp ............................................................  2831
GRP_h1_3647bp ...C.......GC..........................C....................  2831
IYK_h2_3647bp ...C.......GC..........................C....................  2831
HSS_h2_3657bp ...C.......GC..........................C....................  2831
SWT_h2_3657bp ...C.......GC..........................C....................  2831
KNN_h2_3647bp ...C.......GC..........................C....................  2831
STS_h1_3647bp ...C.......GC..........................C....................  2831
PMM_H_3647bp  ...C.......GC..........................C....................  2831
KSH_h2_3647bp ......T.....................................................  2831
MKK_h2_3647bp ......T.....................................................  2831
PNK_h2_3647bp ............................................................  2831
KNN_h1_3647bp ...C........................................................  2831
STS_h2_3647bp ...C........................................................  2831
MKK_h1_3647bp ...C........................................................  2831
LMN_h1_3647bp ...C........................................................  2831
HSS_h1_3647bp ...C........................................................  2831
KSH_h1_3647bp ...C........................................................  2831
```

```
KNG_h2_3647bp .................................T..................T.......  2831
MRC_H_3647bp  .................................T..................T.......  2831
MDT_h2_3647bp .................................T..................T.......  2831
DNC_h2_3647bp .................................T..................T.......  2831
GRP_h2_3647bp ............................................................  2831
DNC_h1_3647bp ............................................................  2831
KNG_h1_3647bp ............................................................  2831
PNK_h1_3647bp ............................................................  2831
SWT_h1_3647bp ............................................................  2831
IYK_h1_3647bp ............................................................  2831
CLM_3647bp    ............................................................  2831

Consensus     CTCAATACCCGATTCGATTAGCAATCTAAGAGACCTCATTCTCTTGGATTTAAGCTATAA  2926
OVL_h1_3647bp ....................................T.................T.....  2891
CTR_H_3648bp  ............................................................  2891
LMN_h2_3648bp ............................................................  2891
HYG_H_3647bp  ............................................................  2891
MDT_h1_3647bp ............................................................  2891
GRP_h1_3647bp ............................................................  2891
IYK_h2_3647bp ............................................................  2891
HSS_h2_3657bp ............................................................  2891
SWT_h2_3657bp ............................................................  2891
KNN_h2_3647bp ............................................................  2891
STS_h1_3647bp ............................................................  2891
PMM_H_3647bp  ............................................................  2891
KSH_h2_3647bp ............................................................  2891
MKK_h2_3647bp ............................................................  2891
PNK_h2_3647bp ............................................................  2891
KNN_h1_3647bp ............................................................  2891
STS_h2_3647bp ............................................................  2891
MKK_h1_3647bp ............................................................  2891
LMN_h1_3647bp ............................................................  2891
HSS_h1_3647bp ............................................................  2891
KSH_h1_3647bp ............................................................  2891
KNG_h2_3647bp ............................................................  2891
MRC_H_3647bp  ............................................................  2891
MDT_h2_3647bp ............................................................  2891
DNC_h2_3647bp ............................................................  2891
GRP_h2_3647bp ............................................................  2891
DNC_h1_3647bp ............................................................  2891
KNG_h1_3647bp ............................................................  2891
PNK_h1_3647bp ............................................................  2891
SWT_h1_3647bp ............................................................  2891
IYK_h1_3647bp ............................................................  2891
CLM_3647bp    ............................................................  2891

Consensus     TCATCTCTCGGGTCCATTTCCTATATCTATTCGAAATTTAAATTCTCTCCAAGCCTTGAT  2986
OVL_h1_3647bp ......................T.....................................  2951
CTR_H_3648bp  ............................................................  2951
LMN_h2_3648bp ............................................................  2951
HYG_H_3647bp  ............................................................  2951
MDT_h1_3647bp ............................................................  2951
GRP_h1_3647bp ............................................................  2951
IYK_h2_3647bp ............................................................  2951
HSS_h2_3657bp ............................................................  2951
SWT_h2_3657bp ............................................................  2951
KNN_h2_3647bp ............................................................  2951
STS_h1_3647bp ............................................................  2951
PMM_H_3647bp  ............................................................  2951
KSH_h2_3647bp ............................................................  2951
MKK_h2_3647bp ............................................................  2951
PNK_h2_3647bp ............................................................  2951
KNN_h1_3647bp ............................................................  2951
STS_h2_3647bp ............................................................  2951
MKK_h1_3647bp ............................................................  2951
LMN_h1_3647bp ............................................................  2951
HSS_h1_3647bp ............................................................  2951
KSH_h1_3647bp ............................................................  2951
```

```
KNG_h2_3647bp ............................................................  2951
MRC_H_3647bp  ............................................................  2951
MDT_h2_3647bp ............................................................  2951
DNC_h2_3647bp ............................................................  2951
GRP_h2_3647bp ............................................................  2951
DNC_h1_3647bp ............................................................  2951
KNG_h1_3647bp ............................................................  2951
PNK_h1_3647bp ............................................................  2951
SWT_h1_3647bp ............................................................  2951
IYK_h1_3647bp ............................................................  2951
CLM_3647bp    ............................................................  2951

Consensus     TCTAAAAAGCAACTCAATGGGTCCAATAACAATTCCTAATTATAGTTTCATTGGCATGAG  3046
OVL_h1_3647bp ............................................................  3011
CTR_H_3648bp  .....................................G......................  3011
LMN_h2_3648bp .....................................G......................  3011
HYG_H_3647bp  .........T.............................................G....  3011
MDT_h1_3647bp .........T.............................................G....  3011
GRP_h1_3647bp ............................................................  3011
IYK_h2_3647bp ............................................................  3011
HSS_h2_3657bp ............................................................  3011
SWT_h2_3657bp ............................................................  3011
KNN_h2_3647bp ............................................................  3011
STS_h1_3647bp ............................................................  3011
PMM_H_3647bp  ............................................................  3011
KSH_h2_3647bp ............................................................  3011
MKK_h2_3647bp ............................................................  3011
PNK_h2_3647bp ............................................................  3011
KNN_h1_3647bp ............................................................  3011
STS_h2_3647bp ............................................................  3011
MKK_h1_3647bp ............................................................  3011
LMN_h1_3647bp ............................................................  3011
HSS_h1_3647bp ............................................................  3011
KSH_h1_3647bp ............................................................  3011
KNG_h2_3647bp .......................................................G....  3011
MRC_H_3647bp  .......................................................G....  3011
MDT_h2_3647bp .......................................................G....  3011
DNC_h2_3647bp .......................................................G....  3011
GRP_h2_3647bp .........T.............................................G....  3011
DNC_h1_3647bp .........T.............................................G....  3011
KNG_h1_3647bp .........T.............................................G....  3011
PNK_h1_3647bp .........T.............................................G....  3011
SWT_h1_3647bp .........T.............................................G....  3011
IYK_h1_3647bp .........T.............................................G....  3011
CLM_3647bp    .........T.............................................G....  3011

Consensus     AAACTTAATGATACTTATTTTATCAAACATGAATTTGCGCGGTCCAATCCCCGAGTCACT  3106
OVL_h1_3647bp ............................................................  3071
CTR_H_3648bp  .............................................C..............  3071
LMN_h2_3648bp .............................................C..............  3071
HYG_H_3647bp  ............................................................  3071
MDT_h1_3647bp ............................................................  3071
GRP_h1_3647bp G...........C.................T.............................  3071
IYK_h2_3647bp G...........C.................T.............................  3071
HSS_h2_3657bp ............................................................  3071
SWT_h2_3657bp ............................................................  3071
KNN_h2_3647bp G...........................................................  3071
STS_h1_3647bp G...........................................................  3071
PMM_H_3647bp  G...........................................................  3071
KSH_h2_3647bp ....C.......................................................  3071
MKK_h2_3647bp ....C.......................................................  3071
PNK_h2_3647bp ............................................................  3071
KNN_h1_3647bp ...............................G............................  3071
STS_h2_3647bp ...............................G............................  3071
MKK_h1_3647bp ...............................G............................  3071
LMN_h1_3647bp ...............................G............................  3071
HSS_h1_3647bp ...............................G............................  3071
KSH_h1_3647bp ...............................G............................  3071
```

```
KNG_h2_3647bp ............................................................  3071
MRC_H_3647bp  ............................................................  3071
MDT_h2_3647bp ............................................................  3071
DNC_h2_3647bp ............................................................  3071
GRP_h2_3647bp ............................................................  3071
DNC_h1_3647bp ............................................................  3071
KNG_h1_3647bp ............................................................  3071
PNK_h1_3647bp ............................................................  3071
SWT_h1_3647bp ............................................................  3071
IYK_h1_3647bp ............................................................  3071
CLM_3647bp    ............................................................  3071

Consensus     AGGCCAGTTACCAAATCTTCACGTACTTCATCTAGATGAAAATCACCTCAATGGCTCAAT  3166
OVL_h1_3647bp ............................................................  3131
CTR_H_3648bp  ....................................C.......................  3131
LMN_h2_3648bp ....................................C.......................  3131
HYG_H_3647bp  ...............................T............................  3131
MDT_h1_3647bp ...............................T............................  3131
GRP_h1_3647bp ............................................................  3131
IYK_h2_3647bp ............................................................  3131
HSS_h2_3657bp ................................................G...........  3131
SWT_h2_3657bp ................................................G...........  3131
KNN_h2_3647bp ............................................................  3131
STS_h1_3647bp ............................................................  3131
PMM_H_3647bp  ............................................................  3131
KSH_h2_3647bp ............................................................  3131
MKK_h2_3647bp ............................................................  3131
PNK_h2_3647bp G...........................................................  3131
KNN_h1_3647bp ............................................................  3131
STS_h2_3647bp ............................................................  3131
MKK_h1_3647bp ............................................................  3131
LMN_h1_3647bp ............................................................  3131
HSS_h1_3647bp ............................................................  3131
KSH_h1_3647bp ............................................................  3131
KNG_h2_3647bp ............................................................  3131
MRC_H_3647bp  ............................................................  3131
MDT_h2_3647bp ............................................................  3131
DNC_h2_3647bp ............................................................  3131
GRP_h2_3647bp ...............................T............................  3131
DNC_h1_3647bp ...............................T............................  3131
KNG_h1_3647bp ...............................T............................  3131
PNK_h1_3647bp ...............................T............................  3131
SWT_h1_3647bp ...............................T............................  3131
IYK_h1_3647bp ...............................T............................  3131
CLM_3647bp    ...............................T............................  3131

Consensus     TCCCAACAGTTTCAAAAACTTGAAGCATGTTAGTGAATTAAGACTAAACAATAATCAATT  3226
OVL_h1_3647bp ...............G.......................................GG...  3191
CTR_H_3648bp  C....G....................................G.................  3191
LMN_h2_3648bp C....G....................................G.................  3191
HYG_H_3647bp  .....G......................................................  3191
MDT_h1_3647bp .....G......................................................  3191
GRP_h1_3647bp .......................................................GG...  3191
IYK_h2_3647bp .......................................................GG...  3191
HSS_h2_3657bp ...............G.......................................GG...  3191
SWT_h2_3657bp ...............G.......................................GG...  3191
KNN_h2_3647bp ...T...........G.......................................GG...  3191
STS_h1_3647bp ...T...........G.......................................GG...  3191
PMM_H_3647bp  ...T...........G.......................................GG...  3191
KSH_h2_3647bp ............................................................  3191
MKK_h2_3647bp ............................................................  3191
PNK_h2_3647bp ...............G.......................................GG...  3191
KNN_h1_3647bp ............................................................  3191
STS_h2_3647bp ............................................................  3191
MKK_h1_3647bp ............................................................  3191
LMN_h1_3647bp ............................................................  3191
HSS_h1_3647bp ............................................................  3191
KSH_h1_3647bp ............................................................  3191
```

```
KNG_h2_3647bp ...............G.......................................GG...  3191
MRC_H_3647bp  ...............G.......................................GG...  3191
MDT_h2_3647bp ...............G.......................................GG...  3191
DNC_h2_3647bp ...............G.......................................GG...  3191
GRP_h2_3647bp .....G......................................................  3191
DNC_h1_3647bp .....G......................................................  3191
KNG_h1_3647bp .....G......................................................  3191
PNK_h1_3647bp .....G......................................................  3191
SWT_h1_3647bp .....G......................................................  3191
IYK_h1_3647bp .....G......................................................  3191
CLM_3647bp    .....G......................................................  3191

Consensus     AACAGGGCCACTTCCATTCGAGAGAGAAATGGTATGGAAGATGAAGAGCAAGCTTAGACT  3286
OVL_h1_3647bp ...............................................A............  3251
CTR_H_3648bp  ...............G......................................C...G.  3251
LMN_h2_3648bp ...............G......................................C...G.  3251
HYG_H_3647bp  ............................................................  3251
MDT_h1_3647bp ............................................................  3251
GRP_h1_3647bp ............................................................  3251
IYK_h2_3647bp ............................................................  3251
HSS_h2_3657bp ............................................................  3251
SWT_h2_3657bp ............................................................  3251
KNN_h2_3647bp ........................................................T...  3251
STS_h1_3647bp ........................................................T...  3251
PMM_H_3647bp  ........................................................T...  3251
KSH_h2_3647bp ...............G......................................C.....  3251
MKK_h2_3647bp ...............G......................................C.....  3251
PNK_h2_3647bp ............................................................  3251
KNN_h1_3647bp ...............G............................................  3251
STS_h2_3647bp ...............G............................................  3251
MKK_h1_3647bp ...............G............................................  3251
LMN_h1_3647bp ...............G............................................  3251
HSS_h1_3647bp ...............G............................................  3251
KSH_h1_3647bp ...............G............................................  3251
KNG_h2_3647bp ......................................................C.....  3251
MRC_H_3647bp  ......................................................C.....  3251
MDT_h2_3647bp ......................................................C.....  3251
DNC_h2_3647bp ......................................................C.....  3251
GRP_h2_3647bp ............................................................  3251
DNC_h1_3647bp ............................................................  3251
KNG_h1_3647bp ............................................................  3251
PNK_h1_3647bp ............................................................  3251
SWT_h1_3647bp ............................................................  3251
IYK_h1_3647bp ............................................................  3251
CLM_3647bp    ............................................................  3251

Consensus     TCATAACAACTCAGGTCTGTGTTACAATGCAGGTAGCGGTTTCGAAGACGGTTTAGATTC  3346
OVL_h1_3647bp ............................................................  3311
CTR_H_3648bp  ............................................................  3311
LMN_h2_3648bp ............................................................  3311
HYG_H_3647bp  .A..........................................................  3311
MDT_h1_3647bp .A..........................................................  3311
GRP_h1_3647bp ............................................................  3311
IYK_h2_3647bp ............................................................  3311
HSS_h2_3657bp ..............................A.............................  3311
SWT_h2_3657bp ..............................A.............................  3311
KNN_h2_3647bp ............................................................  3311
STS_h1_3647bp ............................................................  3311
PMM_H_3647bp  ............................................................  3311
KSH_h2_3647bp .A........C.................................................  3311
MKK_h2_3647bp .A........C.................................................  3311
PNK_h2_3647bp ............................................................  3311
KNN_h1_3647bp ............................................................  3311
STS_h2_3647bp ............................................................  3311
MKK_h1_3647bp ............................................................  3311
LMN_h1_3647bp ............................................................  3311
HSS_h1_3647bp ............................................................  3311
KSH_h1_3647bp ............................................................  3311
```

```
KNG_h2_3647bp   ............................................................  3311
MRC_H_3647bp    ............................................................  3311
MDT_h2_3647bp   ............................................................  3311
DNC_h2_3647bp   ............................................................  3311
GRP_h2_3647bp   .A..........................................................  3311
DNC_h1_3647bp   .A..........................................................  3311
KNG_h1_3647bp   .A..........................................................  3311
PNK_h1_3647bp   .A..........................................................  3311
SWT_h1_3647bp   .A..........................................................  3311
IYK_h1_3647bp   .A..........................................................  3311
CLM_3647bp      .A..........................................................  3311

Consensus       ATCGATTGATTCGGGTATCGGTTTATGTGACTCGGGTAAACCGGGTTCAGCAAGATCAGT  3406
OVL_h1_3647bp   ........................G.............GG..T.................  3371
CTR_H_3648bp    ..............................G.............................  3371
LMN_h2_3648bp   ..............................G.............................  3371
HYG_H_3647bp    ..............................G.............................  3371
MDT_h1_3647bp   ..............................G.............................  3371
GRP_h1_3647bp   ..............................G.............................  3371
IYK_h2_3647bp   ..............................G.............................  3371
HSS_h2_3657bp   ..............................G......................AT.....  3371
SWT_h2_3657bp   ..............................G......................AT.....  3371
KNN_h2_3647bp   ..............................G.............................  3371
STS_h1_3647bp   ..............................G.............................  3371
PMM_H_3647bp    ..............................G.............................  3371
KSH_h2_3647bp   ........................G.............GG....................  3371
MKK_h2_3647bp   ........................G.............GG....................  3371
PNK_h2_3647bp   ........................G.....A.............................  3371
KNN_h1_3647bp   ............................................................  3371
STS_h2_3647bp   ............................................................  3371
MKK_h1_3647bp   ............................................................  3371
LMN_h1_3647bp   ............................................................  3371
HSS_h1_3647bp   ............................................................  3371
KSH_h1_3647bp   ............................................................  3371
KNG_h2_3647bp   ............................................................  3371
MRC_H_3647bp    ............................................................  3371
MDT_h2_3647bp   ............................................................  3371
DNC_h2_3647bp   ............................................................  3371
GRP_h2_3647bp   ............................................................  3371
DNC_h1_3647bp   ............................................................  3371
KNG_h1_3647bp   ............................................................  3371
PNK_h1_3647bp   ............................................................  3371
SWT_h1_3647bp   ............................................................  3371
IYK_h1_3647bp   ............................................................  3371
CLM_3647bp      ............................................................  3371

Consensus       GCAACATCTCGGCACCCTGGAAGAAAATATAACGGGAACAATAAATACATCAGTTGGTAC  3466
OVL_h1_3647bp   ............................................................  3431
CTR_H_3648bp    .....................................................G......  3431
LMN_h2_3648bp   .....................................................G......  3431
HYG_H_3647bp    A...........................................................  3431
MDT_h1_3647bp   A...........................................................  3431
GRP_h1_3647bp   ............................................................  3431
IYK_h2_3647bp   ............................................................  3431
HSS_h2_3657bp   .............................................C..............  3431
SWT_h2_3657bp   .............................................C..............  3431
KNN_h2_3647bp   .............................................C..............  3431
STS_h1_3647bp   .............................................C..............  3431
PMM_H_3647bp    .............................................C..............  3431
KSH_h2_3647bp   ............................................................  3431
MKK_h2_3647bp   ............................................................  3431
PNK_h2_3647bp   ............................................................  3431
KNN_h1_3647bp   ............................................................  3431
STS_h2_3647bp   ............................................................  3431
MKK_h1_3647bp   ............................................................  3431
LMN_h1_3647bp   ............................................................  3431
HSS_h1_3647bp   ............................................................  3431
KSH_h1_3647bp   ............................................................  3431
```

```
KNG_h2_3647bp  ............................................................  3431
MRC_H_3647bp   ............................................................  3431
MDT_h2_3647bp  ............................................................  3431
DNC_h2_3647bp  ............................................................  3431
GRP_h2_3647bp  ............................................................  3431
DNC_h1_3647bp  ............................................................  3431
KNG_h1_3647bp  ............................................................  3431
PNK_h1_3647bp  ............................................................  3431
SWT_h1_3647bp  ............................................................  3431
IYK_h1_3647bp  ............................................................  3431
CLM_3647bp     ............................................................  3431

Consensus      AGTGCATGATGCTTCTAATTTTGTTAGGTCGCTTCAGTTAATATCAGTTGTTTTGATGAT  3526
OVL_h1_3647bp  ..C..........................T..............................  3491
CTR_H_3648bp   .............................T..........GC..................  3491
LMN_h2_3648bp  .............................T..........GC..................  3491
HYG_H_3647bp   ........................C....T...................C..........  3491
MDT_h1_3647bp  ........................C....T...................C..........  3491
GRP_h1_3647bp  .......A.......C.............T.......C......................  3491
IYK_h2_3647bp  .......A.......C.............T.......C......................  3491
HSS_h2_3657bp  ...............C.............T.......C...........A..........  3491
SWT_h2_3657bp  ...............C.............T.......C...........A..........  3491
KNN_h2_3647bp  ...............C.............T...................A..........  3491
STS_h1_3647bp  ...............C.............T...................A..........  3491
PMM_H_3647bp   ...............C.............T...................A..........  3491
KSH_h2_3647bp  .A.....A.......C.............T..............................  3491
MKK_h2_3647bp  .A.....A.......C.............T..............................  3491
PNK_h2_3647bp  .....................................C......................  3491
KNN_h1_3647bp  ............................................................  3491
STS_h2_3647bp  ............................................................  3491
MKK_h1_3647bp  ............................................................  3491
LMN_h1_3647bp  ............................................................  3491
HSS_h1_3647bp  ............................................................  3491
KSH_h1_3647bp  ............................................................  3491
KNG_h2_3647bp  ............................................................  3491
MRC_H_3647bp   ............................................................  3491
MDT_h2_3647bp  ............................................................  3491
DNC_h2_3647bp  ............................................................  3491
GRP_h2_3647bp  ..........A.................................................  3491
DNC_h1_3647bp  ..........A.................................................  3491
KNG_h1_3647bp  ..........A.................................................  3491
PNK_h1_3647bp  ..........A.................................................  3491
SWT_h1_3647bp  ..........A.................................................  3491
IYK_h1_3647bp  ..........A.................................................  3491
CLM_3647bp     ..........A.................................................  3491

Consensus      TTTATTTTCGTTGTAAGAAGCTAAATGAAGGGGTGTAAAGTAGTATAAAGTATAGTGCAA  3586
OVL_h1_3647bp  ............................................................  3551
CTR_H_3648bp   ....................T.....AG....A.......................A...  3551
LMN_h2_3648bp  ....................T.....AG....A.......................A...  3551
HYG_H_3647bp   .........A.........A......A.....AC.....A....................  3551
MDT_h1_3647bp  .........A.........A......A.....AC.....A....................  3551
GRP_h1_3647bp  .........A..................................................  3551
IYK_h2_3647bp  .........A..................................................  3551
HSS_h2_3657bp  .........A..................................................  3551
SWT_h2_3657bp  .........A..................................................  3551
KNN_h2_3647bp  .........A........................T.........................  3551
STS_h1_3647bp  .........A........................T.........................  3551
PMM_H_3647bp   .........A........................T.........................  3551
KSH_h2_3647bp  ............................................................  3551
MKK_h2_3647bp  ............................................................  3551
PNK_h2_3647bp  ............................................................  3551
KNN_h1_3647bp  ............................................................  3551
STS_h2_3647bp  ............................................................  3551
MKK_h1_3647bp  ............................................................  3551
LMN_h1_3647bp  ............................................................  3551
HSS_h1_3647bp  ............................................................  3551
KSH_h1_3647bp  ............................................................  3551
```

```
KNG_h2_3647bp  ............................................................  3551
MRC_H_3647bp   ............................................................  3551
MDT_h2_3647bp  ............................................................  3551
DNC_h2_3647bp  ............................................................  3551
GRP_h2_3647bp  ............................................................  3551
DNC_h1_3647bp  ............................................................  3551
KNG_h1_3647bp  ............................................................  3551
PNK_h1_3647bp  ............................................................  3551
SWT_h1_3647bp  ............................................................  3551
IYK_h1_3647bp  ............................................................  3551
CLM_3647bp     ............................................................  3551

Consensus      TACTAC-GTCTTATAAATTTGTTCTTTCAAAGTAA----------TTTATAACAAGCACC  3635
OVL_h1_3647bp  ......-............................----------...............  3600
CTR_H_3648bp   ......C...........A.............C..----------..............A  3601
LMN_h2_3648bp  ......C...........A.............C..----------..............A  3601
HYG_H_3647bp   .....T-................T.....C.....----------...............  3600
MDT_h1_3647bp  .....T-................T.....C.....----------...............  3600
GRP_h1_3647bp  ......-............................----------...............  3600
IYK_h2_3647bp  ......-............................----------...............  3600
HSS_h2_3657bp  ......-............................TTTATAGTAA..............A  3610
SWT_h2_3657bp  ......-............................TTTATAGTAA..............A  3610
KNN_h2_3647bp  ......-............................----------..............T  3600
STS_h1_3647bp  ......-............................----------..............T  3600
PMM_H_3647bp   ......-............................----------..............T  3600
KSH_h2_3647bp  ......-............................----------.............T.  3600
MKK_h2_3647bp  ......-............................----------.............T.  3600
PNK_h2_3647bp  ......-............................----------..C............  3600
KNN_h1_3647bp  ......-............................----------...............  3600
STS_h2_3647bp  ......-............................----------...............  3600
MKK_h1_3647bp  ......-............................----------...............  3600
LMN_h1_3647bp  ......-............................----------...............  3600
HSS_h1_3647bp  ......-............................----------...............  3600
KSH_h1_3647bp  ......-............................----------...............  3600
KNG_h2_3647bp  ......-............................----------...............  3600
MRC_H_3647bp   ......-............................----------...............  3600
MDT_h2_3647bp  ......-............................----------...............  3600
DNC_h2_3647bp  ......-............................----------...............  3600
GRP_h2_3647bp  ......-............................----------...............  3600
DNC_h1_3647bp  ......-............................----------...............  3600
KNG_h1_3647bp  ......-............................----------...............  3600
PNK_h1_3647bp  ......-............................----------...............  3600
SWT_h1_3647bp  ......-............................----------...............  3600
IYK_h1_3647bp  ......-............................----------...............  3600
CLM_3647bp     ......-............................----------...............  3600

Consensus      TGTAACGTTTTAAAGTAATTAAGATGAACTTATTGGGATTCTSTCGG               3682
OVL_h1_3647bp  ...............................GG.........TG.AA               3647
CTR_H_3648bp   ................................G.........CG.AA               3648
LMN_h2_3648bp  ................................G.........CG.AA               3648
HYG_H_3647bp   ..................................A.TGGGA.TCTC.               3647
MDT_h1_3647bp  ..................................A.TGGGA.TCTC.               3647
GRP_h1_3647bp  ..................................A.TGGGA.TCTC.               3647
IYK_h2_3647bp  ..................................A.TGGGA.TCTC.               3647
HSS_h2_3657bp  ..................................A.TGGGA.TCTC.               3657
SWT_h2_3657bp  ..................................A.TGGGA.TCTC.               3657
KNN_h2_3647bp  ..................................A.TGGGA.TCTC.               3647
STS_h1_3647bp  ..................................A.TGGGA.TCTC.               3647
PMM_H_3647bp   ..................................A.TGGGA.TCTC.               3647
KSH_h2_3647bp  ................................G.........CA.AA               3647
MKK_h2_3647bp  ................................G.........CA.AA               3647
PNK_h2_3647bp  ...............................GG.........CG..A               3647
KNN_h1_3647bp  ...............................GG.........CG.AA               3647
STS_h2_3647bp  ...............................GG.........CG.AA               3647
MKK_h1_3647bp  ...............................GG.........CG.AA               3647
LMN_h1_3647bp  ...............................GG.........CG.AA               3647
HSS_h1_3647bp  ...............................GG.........CG.AA               3647
KSH_h1_3647bp  ...............................GG.........CG.AA               3647
```

```
KNG_h2_3647bp ...........................G.CAC..C..CC.TGG....                3647
MRC_H_3647bp  ...........................G.CAC..C..CC.TGG....                3647
MDT_h2_3647bp ...........................G.CAC..C..CC.TGG....                3647
DNC_h2_3647bp ...........................G.CAC..C..CC.TGG....                3647
GRP_h2_3647bp ...........................G.CAC..C..CC.TGG....                3647
DNC_h1_3647bp ...........................G.CAC..C..CC.TGG....                3647
KNG_h1_3647bp ...........................G.CAC..C..CC.TGG....                3647
PNK_h1_3647bp ...........................G.CAC..C..CC.TGG....                3647
SWT_h1_3647bp ...........................G.CAC..C..CC.TGG....                3647
IYK_h1_3647bp ...........................G.CAC..C..CC.TGG....                3647
CLM_3647bp    ...........................G.CAC..C..CC.TGG....                3647
```
