# Supplementary material for: AlleleMiner: a long-read pipeline for gene-wise de novo allele phasing and variant detection in diploid citrus cultivars
Source: DNA Res. 2026 Mar 3;33(2):dsag004. doi: 10.1093/dnares/dsag004 (PMC13011809; doi:10.1093/dnares/dsag004)
Supplement: dsag004_Supplementary_Data [file dsag004_supplementary_data.zip › Kiryu_Supplementary_Table_S1_260216.pdf]

Supplementary Table S1. Basic read statistics. The table presents the basic statistics of the long-read data used for the analysis.

Sample	Mukaku kishu	Lemon	Citron	Oval kumquat
Mean read length (bp)	21,106.0	20,123.5	21,752.6	22,642.5
Mean read quality	26.5	26.5	25.8	25.6
Median read length (bp)	20,456.0	19,419.0	20,874.0	21,699.0
Median read quality	28.9	28.9	27.7	27.3
Number of reads	322,989	388,053	394,590	314,963
Read length N50 (bp)	21,254	20,447	22,202	23,238
STDEV read length (bp)	4,068.9	4,348.1	5,196.9	5,726.7
Total bases (bp)	6,817,011,601	7,809,001,423	8,583,375,905	7,131,545,949
