# Supplementary material for: AlleleMiner: a long-read pipeline for gene-wise de novo allele phasing and variant detection in diploid citrus cultivars
Source: DNA Res. 2026 Mar 3;33(2):dsag004. doi: 10.1093/dnares/dsag004 (PMC13011809; doi:10.1093/dnares/dsag004)
Supplement: dsag004_Supplementary_Data [file dsag004_supplementary_data.zip › Kiryu_Supplementary_Table_S2_260216.pdf]

Supplementary Table S2. Number of failures per cultivar at each step of the AllerMiner workflow and coverage

Failure step of workflow in Fig. 1 / Cultivar	Number of failures																		Average	Rate of failure (%)
	STS	KSH	KNN	CTR	MDT	LMN	PNK	OVL	IYK	MKK	HYG	KNG	HSS	SWT	GRP	MRC	PMM	DNC		
Mapping	0	0	0	2	0	1	1	2	1	0	1	0	3	0	0	1	1	1	0.8	0.6
Read Selection	6	11	10	51	4	21	7	28	11	13	13	18	13	8	12	14	14	13	14.8	12.4
HiFi de novo Assembly	14	19	31	29	49	59	47	83	74	57	100	76	128	119	238	149	192	236	94.4	78.8
Allele Extraction	1	7	2	24	6	22	6	28	5	8	8	7	8	4	14	7	17	3	9.8	8.2
Coverage	42.9	30.9	26.3	23.8	23.0	21.7	21.5	19.8	19.2	18.9	18.2	17.9	16.7	16.0	13.5	13.3	13.2	11.3		
