# Supplementary material for: AlleleMiner: a long-read pipeline for gene-wise de novo allele phasing and variant detection in diploid citrus cultivars
Source: DNA Res. 2026 Mar 3;33(2):dsag004. doi: 10.1093/dnares/dsag004 (PMC13011809; doi:10.1093/dnares/dsag004)
Supplement: dsag004_Supplementary_Data [file dsag004_supplementary_data.zip › Kiryu_Supplementary_Table_S3_260216.pdf]

Supplementary Table S3. Example of a summary list output from AlleleMiner.

Allele sequence length, Jaccard coefficients, and MinHash values were used to quantitatively evaluate heterozygosity and allele transmission inferred by AlleleMiner. Differences in allele length indicate potential structural variation, while Jaccard coefficients effect sequence similarity between heteroallelic pairs. MinHash values are used to detect allele sharing between different varieties when AlleleMiner is applied to multiple samples.

seq_name	zygosity	length	MinHash_info	hash_string	jaccard_similarity
MDT_CUN1G201600.t1_H	homo	2529	<datasketch.minhash.MinHash object at 0x7d71dcddc8e0>	000000000036aeb9000000000013ee6000000000001cdd8f000000000019fb8b000000000011d8b20000000000453b580000000000806f18000000000014c2220000000000016180f00000000000c8163 00000000005473d0000000000009fa400000000000037809c00000000024328f00000000001ae4df000000000010fc1e00000000000c8cee0000000000069ac8000000000006c4a300000000001272f10 0000000001ce3380000000000001d3e5500000000002a167a00000000004df70600000000001afe9a0000000000057f0d00000000000f0dcd0000000000072d680000000001100b7000000000019a3fa0 000000000141fce00000000002c7f62000000000000087be6000000000004fecc0000000008e5c7000000000003100d00000000000597420000000000031faee00000000001a7eca00000000000022ddf800 0000000011b26f000000000000152dc8000000000000847e700000000000019a5460000000000000f18a2000000000006b5360000000000029150b00000000002881c000000000005d7b40000000000127bf700 00000000180be700000000000a17e500000000000121934000000000000020cb32000000000001d11fc000000000000070bab00000000000034c1500000000000d36b1000000000008c52f00000000000292ea70 0000000001271000000000000008f00100000000000795840000000000001a34f50000000000134aff00000000019579e000000000031a7fe00000000000d44d20000000000156710000000000000a0c200 000000002c814800000000000381c580000000000002dc3f00000000000175ef800000000000011637b0000000000148f6e00000000003ec362000000000009189200000000000087710000000000007407f000 00000002011b30000000000000c05da000000000016c31f00000000007dd2900000000000c175d00000000000077c440000000000287ae70000000000660c400000000000064dfb0000000000154a20000 000000003dc6c000000000010f98a00000000000b245a00000000000bd4e00000000001f500a00000000000bda6900000000002e86eb00000000000824b10000000002e47c800000000003b815100 0000000006533e00000000000c99a400000000001baefe000000000011784500000000000f271000000000003b8e4e000000000003994800000000000006048a00000000004aa04d00000000000000b300 00000000006d67f00000000001ad941000000000017c2e300000000000139e500000000000cf4f700000000023b490000000000003a620000000000000bab3000000000000f904300000000000ab5050 000000000147c2100000000000011e806000000000010667800000000000039c104000000000401c390000000000044fc900000000000080c72000000000014888f	-
MDT_CUN2G259500.t1_h1	hetero	7314	<datasketch.minhash.MinHash object at 0x7d71dcddcac0>	000000000000e16500000000000000ef2600000000000004260f00000000000856ce00000000000210c200000000000a2e3300000000000011be8c00000000000a840a000000000002c5da000000000002ad87 00000000000aba4d000000000001637360000000000000bc24b0000000000024e4e00000000000157110000000000000989b50000000000009575000000000000943260000000000010f8000000000000ce8b 20000000000141598000000000000864260000000000015265b000000000003d6e7000000000016495d00000000000477b2000000000004a04e00000000000754280000000000000e4bd7000000000000100 958000000000026d779000000000000782f800000000000090d8000000000076c9e000000000001037000000000006da96000000000001c74400000000000008e8b0000000000008846200000000000000d 9ad800000000000f7fc90000000000015a3a9000000000045c9800000000004ebd6000000000027f8d000000000000ba2030000000000000888fb000000000317cf6000000000004d72c000000000001f c2d00000000000051b550000000000000270dd00000000000004f955000000000000621010000000000000630db0000000000133b3000000000001279700000000000008e146000000000008388e000000000000 659a000000000013a351000000000000245fd0000000001270af00000000000f2e90000000000004254900000000002c3f9c000000000014017b000000000008943b0000000000117e3a000000000003 978100000000000d9cf7000000000000cc1ff00000000000a41840000000000003760100000000000007d0c5000000000000007b800000000000410b00000000000b7be800000000003741d0000000000001 bf73000000000000065d7500000000000005f8f40000000000000c4d3000000000001459470000000000006fede00000000000b9fed0000000000cde2e0000000000059c45000000000000496550000000000000097 18e00000000000116f9f000000000008885700000000000644c300000000001a48300000000000053c170000000000001ab1d00000000000409cf0000000000795470000000000000011cd000000000006fe cf000000000001cefec000000000003f92a00000000000084ce7000000000014b1870000000000000bad0b000000000001064800000000000006e4de00000000000005804b0000000000008d836000000000002044 bf000000000000363e0000000000002ff3800000000000153e6100000000000000cbcb00000000001eb1290000000000003ace20000000000000f49a40000000000008f30700000000000375b1000000000001b55 a0000000000007492d0000000000046322000000000000238760000000000007055b0000000000070ac20000000000005581100000000000071b30000000000108147	vs MDT_CUN2G259500.t1_h2 = 0.5546875
MDT_CUN2G259500.t1_h2	hetero	12418	<datasketch.minhash.MinHash object at 0x7d71dcddc610>	000000000000e165000000000000ef26000000000004260f000000000009d7a50000000000000210c200000000000008a830000000000062ce00000000000a840a000000000002c5da000000000002ad87 00000000000aba4d000000000000b0fdb00000000000bc24b0000000000024e4e00000000000157110000000000000989b50000000000009575000000000013c75e000000000010f800000000000e6d6 200000000006b8680000000000000864260000000000015265b0000000000009970000000000015b63f000000000003c16f000000000004a04e00000000000754280000000000002172e00000000000010095 80000000000018e810000000000000782f800000000000090d80000000000010808000000000001037000000000000135140000000000001c74400000000000008e8b00000000000884620000000000000336 890000000000001ad00000000000000a47420000000000001a1f2500000000000004ebd60000000000027f8d00000000000ba20300000000000012939e000000000317cf6000000000004d72c000000000001fc 2d000000000001716500000000000000270dd000000000000069d0800000000000fdef700000000000006f160000000000000133b3000000000001279700000000000008e1460000000000008388e00000000000065 9a000000000000874bd000000000000245fd0000000001270af00000000000ffce8000000000004254900000000000018f2200000000000014017b000000000001f44a0000000000035b4200000000000036f0 000000000000031f9e0000000000c00ae000000000000e4c400000000000376010000000000007d0c500000000000007b800000000000410b00000000000392140000000000003741d0000000000001bf7 30000000000065d750000000000005f8f40000000000000c4d30000000000145947000000000000004215b0000000000003d4d1000000000000a1a5000000000000218f6000000000006aabc000000000000387d c0000000000f7ef3000000000000888570000000000000644c30000000000000a32300000000000009b27000000000001ab1d000000000078d2b000000000007954700000000000011cd000000000006fec f00000000000392bf0000000000001353000000000000002fed9000000000007245b00000000000091370000000000001064800000000000010b8ec0000000000005804b000000000008d8360000000000008cbd f0000000000068b6f000000000012c3ca000000000003a6e7000000000014d200000000000001eb1290000000000003ace200000000000e7e9000000000008f307000000000000375b1000000000001b55 a0000000000007492d000000000004632200000000000023876000000000007055b000000000001c2380000000000055811000000000000071b3000000000000145dc	vs MDT_CUN2G259500.t1_h1 = 0.5546875
MDT_CUN3G447600.t1_H	homo	10593	<datasketch.minhash.MinHash object at 0x7d71dcddc3a0>	00000000001e62c40000000000007e4d20000000000000c56980000000000079e64000000000001461300000000000002284a000000000001a73200000000000033bd8000000000000e5b50000000000003a5d 90000000000210f700000000000877af00000000000c02960000000000000955fb00000000003456c000000000007514200000000000000508c000000000000343a00000000000a8a86000000000000a94e 90000000000035f530000000000c215000000000001038810000000000001b99600000000000005a13e000000000005dfbe000000000000a390000000000000022d800000000000585cc000000000002daf 0000000000000fdc50000000000000909b000000000006dea600000000000be424000000000000054a350000000000004c6200000000000b3d50000000000001e7e300000000000003b3b00000000000969 040000000000556eb0000000000a48d3000000000000068f3a000000000002fe0800000000000183790000000000000c0990000000000046bfa000000000000070c7000000000008ed660000000000000281 730000000000c172000000000000cf67000000000000277320000000000000050b2000000000015440000000000000343880000000000000024800000000000014f72000000000000eddf0000000000000680 d2000000000049e560000000000138aa2000000000000051ee4000000000000c1b2b00000000004e36f000000000002cac000000000000241c90000000000017a220000000000000681c1000000000000117 93b000000000051c6800000000000cef87000000000000584fc00000000002dd4b0000000000000ae94000000000002c6ee00000000000a3e000000000008d751000000000000785550000000000000012 97e000000000003320d00000000000003fbe00000000001f4020000000000000016b670000000000000f45e000000000009084200000000000005dc9b00000000000e361e00000000000226ac000000000000e1 09e000000000004063b000000000001f0740000000000005756700000000000000dd000000000000aa42600000000000001f644000000000000007628a0000000000058983000000000000008a850000000000000 05b200000000000002b164000000000000187a8000000000002fc490000000000005a327000000000000082f24000000000000c70c00000000002ec1000000000000614e3000000000000cd2c000000000000002 a65400000000000bf1840000000000000016f7e0000000000003b366000000000000e0f1a000000000009deea000000000003ce2c00000000000c7757000000000000cd83f0000000000030e71000000000000003 e0aa000000000002b784000000000000b47a9000000000002cf6000000000000040f3800000000001304830000000000008979000000000001e8e000000000000a4709	-
MDT_CUN7G148100.t1_h1	hetero	1647	<datasketch.minhash.MinHash object at 0x7d71dcddcb80>	00000000008b14900000000000445e06000000000000c5a0e000000000000241c660000000000006afd80000000000062cb7000000000000a28ae0000000000003c135c00000000000965f10000000000002b23c6 0000000000456cc700000000003c30b000000000000023a4b60000000000013cfbc000000000000717d100000000004601910000000000000789ff0000000000006f4e0700000000007fc97a00000000006b43e0 00000000003912c00000000000443d1100000000000cffa000000000002313bd00000000000acd0400000000000031bfd0000000000247334000000000000005a71e00000000000013770000000000003473cb0 0000000001efd8100000000000027dd46000000000000048b93f00000000000025e3c0000000000004ffc3900000000000001a2156000000000007500bb000000000000ca0a6000000000009862740000000000006f38600 000000003444d600000000000230c600000000000061958000000000002a72000000000000017abb200000000000029da9200000000000027720b0000000000260de800000000000210980000000000004de589 0000000000377fca00000000001a4fe200000000003365e200000000003e70f1000000000000249c330000000000001a67b700000000000002485aa0000000000078013500000000000005a831000000000000037c275 000000000071fe76000000000015e64a0000000000028b37d000000000000539c40000000000014e06b000000000003ee6bd000000000006fe1a000000000000f1f040000000000000d28f000000000000016289 00000000002c39a900000000000012bf2250000000000016889f0000000000143622000000000000b3d2e400000000000007c971500000000000000ad270000000000016ea6a000000000002ebca500000000000278b 100000000000840a0000000000008a882000000000000060c23a000000000000ad631800000000000003470dd0000000000000374f700000000000009b300000000000004020bb00000000000038e82000000000000001ad 04000000000004ab920000000000004cbe3000000000001047f000000000000012bfaa0000000000002e6a30000000000001e99680000000000002c1998000000000000af42b0000000000105ce900000000000003c8 3100000000002471a60000000000001405bd00000000000d63260000000000000014d370000000000005075d000000000003cc4d3000000000000f8a1f00000000000003807f00000000000b74ef0000000000014aa 940000000000fc70f0000000000045b09b00000000003509e700000000000043744000000000000050ceaa00000000000634a400000000001b466e000000000012d6260000000000016004800000000001226 af00000000002f3ffc000000000006697da00000000000037193e00000000000035c9210000000000035107700000000000dc975000000000003e5398000000000000007fd06	vs MDT_CUN7G148100.t1_h2 = 0.6015625
MDT_CUN7G148100.t1_h2	hetero	1647	<datasketch.minhash.MinHash object at 0x7d71dcddcac0>	00000000008b1490000000000000586e3000000000000e284a0000000000241c66000000000008b3cc9000000000000001bf6000000000000a28ae0000000000003c135c00000000000965f10000000000002b23c6 000000000079a7950000000000003c30b000000000000023a4b6000000000000be2b100000000000001906b0000000000046019100000000000000789ff0000000000006f4e0700000000000795be5000000000031daf9 0000000000003912c00000000000443d1100000000000cffa000000000002cd51500000000000acd04000000000000031bfd0000000000247334000000000003a3deb0000000000013770000000000000493a8c 00000000001efd8100000000003c5b70000000000002a506d000000000000550d07000000000006a3024000000000001a21560000000000003b58c0000000000000ca0a600000000000031188000000000000496d0 9000000000003444d600000000000027b353000000000000619580000000000003ca44400000000000017abb200000000000029da92000000000000027720b000000000004407fb00000000000210980000000000004de5 890000000000377fca00000000001a4fe200000000003365e2000000000009c02900000000000249c330000000000000213e200000000002485aa000000000780135000000000000019ba12000000000000037c2 75000000000002d0b750000000000015e64a00000000001b6c70000000000000539c4000000000000014e06b00000000003ee6bd00000000000968610000000000000f1f04000000000000d28f0000000000000162 89000000000002c39a900000000000010d32d3000000000001b5afd000000000000f65fe000000000000a2d6400000000000007c9715000000000000ad27000000000000016ea6a00000000002ebca50000000000000278 b10000000000000840a00000000000332e1000000000003304e0000000000000ad631800000000000003470dd0000000000000374f7000000000003c6342000000000000004020bb0000000000037b272000000000000001a d04000000000004ab920000000000004cbe3000000000001047f0000000000012bfaa000000000002e6a300000000000001e99680000000000002c19980000000000000163c05000000000000105ce9000000000000003c 83100000000002471a600000000000001405bd00000000000d632600000000000000351de00000000000005075d00000000000968376000000000000f8a1f00000000000034fac00000000000b74ef000000000014a a9400000000000fc70f00000000000045b09b00000000003509e700000000000051ba790000000000000885d20000000000000634a40000000000059babe00000000000005e92c00000000003c98ad00000000000003a 51ff000000000084e0100000000006a923b0000000000187b7900000000000627a30000000003e1b9300000000000dc975000000000003e539800000000000007fd06	vs MDT_CUN7G148100.t1_h1 = 0.6015625
